# Supplementary material for: Amination of 5-Spiro-Substituted 3-Hydroxy-1,5-dihydro-2H-pyrrol-2-ones
Source: Molecules. 2021 Nov 26;26(23):7179. doi: 10.3390/molecules26237179 (PMC8658906; doi:10.3390/molecules26237179)
Supplement: Supplementary file 1 [file molecules-26-07179-s001.zip › molecules-1486942supp.pdf]

Supporting information

for

Amination of 5-Spiro-substituted 3-Hydroxy-1,5-dihydro-2*H*-pyrrol-2-ones

Ekaterina E. Khramtsova*, Ekaterina A. Lystsova, Evgeniya V. Khokhlova, Maksim V. Dmitriev, Andrey N. Maslivets

Department of Chemistry

Perm State University

ul. Bukireva 15, Perm 614990 (Russian Federation)

*E-mail: caterina.stepanova@psu.ru

Table of contents

General information…………………………………………………………..S3
General procedure to compounds **1a-g**…………………………………………S4
NMR charts of compounds **1a-g**…………………………………………………S7
General procedure to compounds **4a-n**…………………………………………S21
NMR charts of compounds **4a-n**……………………………….……………...S27
General procedure to compounds **3a-n**…………………………………………S55
NMR charts of compounds **3a-n**….………………………………………………S61
Antimicrobial assay by CO-ADD (ESKAPE pathogens)………………………..S88

General information

^{1}H and ^{13}C NMR spectra were acquired on a Bruker Avance III 400 HD spectrometer (at 400 and 100 MHz, respectively) in $CDCl_3$ (stab. with Ag) or DMSO-d_6 using the HMDS signal (in ^{1}H NMR) or solvent residual signals (in ^{13}C NMR, 77.00 for $CDCl_3$, 39.51 for DMSO-d_6; in ^{1}H NMR, 7.26 for $CDCl_3$, 2.50 for DMSO-d_6) as internal standards. IR spectra were recorded on a Perkin–Elmer Spectrum Two spectrometer from mulls in mineral oil. Melting points were measured on a Khimlabpribor PTP apparatus or a Mettler Toledo MP70 apparatus. Elemental analyses were carried out on a Vario MICRO Cube analyzer. The reaction conditions were optimized using UPLC-UV-MS [Waters ACQUITY UPLC I-Class system; Acquity UPLC BEH C18 column, grain size of 1.7 μm; acetonitrile–water as eluents; flow rate of 0.6 mL/min; ACQUITY UPLC PDA eλ Detector (wavelength range of 230–780 nm); Xevo TQD mass detector; electrospray ionization; positive and negative ion detection; ion source temperature of 150 °C; capillary voltage of 3500–4000 V; cone voltage of 20–70 V; vaporizer temperature of 200 °C]. Thin-layer chromatography (TLC) was performed on Merck silica gel 60 F_{254} plates using EtOAc/toluene, 1 : 5 v/v, toluene, EtOAc as eluents.
Starting compounds **2a–g** were obtained according to reported procedures [31] from oxalyl chloride (purchased from commercial vendors) and heterocyclic enamines (obtained according to reported procedures [31] from commercially available reagents).
Toluene for procedures involving compounds **2a–g** was dried over Na before the use. All other solvents and reagents were purchased from commercial vendors and used as received.
Procedures involving compounds **2a–g** were carried out in oven-dried glassware.

General procedure to compounds 1a-g

A suspension of the corresponding compound **2** (3.1 mmol) and dicyclohexylurea (3.1 mmol) in 20 mL of toluene was refluxed for 2 h (until the disappearance of the dark violet color of compound **2**). Then the resulting white precipitate was filtered off to afford the desired compound **1**.

9-Benzoyl-1,3-dicyclohexyl-8-hydroxy-6-(2-hydroxyphenyl)-1,3,6-triazaspiro[4.4]non-8-ene-2,4,7-trione (1a)
Yield: 1.58 g (94%); white solid; mp 285–287 °C.
^{1}H NMR (400 MHz, DMSO-d_6): δ = 9.90 (s, 1 H), 7.75 (m, 2 H), 7.62 (m, 1 H), 7.51 (m, 2 H), 7.24 (m, 1 H), 7.00 (m, 1 H), 6.91 (m, 1 H), 6.81(m, 1 H), 3.84 (m, 1 H), 3.08 (m, 1 H), 2.16-1.96 (m, 2 H), 1.87-1.55 (m, 8 H), 1.46 (m, 2 H), 1.36-1.10 (m, 7 H), 0.95 (m, 1 H) ppm.
^{13}C NMR (100 MHz, DMSO-d_6): δ = 188.5, 169.2, 163.1, 155.5, 154.0, 153.9, 137.3, 132.8, 130.0, 128.6 (2 C), 128.2 (2 C), 126.7, 120.0, 119.0, 116.7, 113.1, 80.6, 52.1, 51.0, 30.0, 29.5, 28.6 (2 C), 25.7, 25.2, 25.2, 25.0, 24.8 (2 C) ppm.
IR (mineral oil): 3354, 3151, 1779, 1723, 1708, 1678 cm^{-1}.
Anal. Calcd (%) for $C_{31}H_{33}N_3O_6$: C 68.49; H 6.12; N 7.73. Found: C 68.23; H 6.13; N 7.76.

1,3-Dicyclohexyl-9-(4-ethoxybenzoyl)-8-hydroxy-6-(2-hydroxyphenyl)-1,3,6-triazaspiro[4.4]non-8-ene-2,4,7-trione (1b)
Yield: 1.78 g (98%); white solid; mp 274–276 °C.
^{1}H NMR (400 MHz, DMSO-d_6): δ = 9.88 (s, 1 H), 7.75 (m, 2 H), 7.23 (m, 1 H), 7.03-6.98 (m, 3 H), 6.90 (m, 1 H), 6.81 (m, 1 H), 4.15 (q, *J* 7.0 Hz, 2 H), 3.84 (m, 1 H), 3.05 (m, 1 H), 2.15-1.97 (m, 2 H),1.83-1.56 (m, 8 H), 1.49-1.41 (m, 2 H), 1.37-1.24 (m, 5 H), 1.21-1.07 (m, 5 H), 0.94 (m, 1 H) ppm.
^{13}C NMR (100 MHz, DMSO-d_6): δ = 186.9, 169.2, 163.2, 162.5, 154.1, 153.9, 131.1 (2 C), 130.0, 129.6, 128.1, 126.7, 120.1, 118.9, 116.6, 113.9 (2 C), 111.9, 80.7, 63.5, 52.1, 51.0, 30.0, 29.5, 28.7 (2 C), 28.6, 25.7, 25.2, 25.0, 24.8 (2 C), 14.4 ppm.
IR (mineral oil): 3386, 3173, 1777, 1727, 1714, 1683 cm^{-1}.
Anal. Calcd (%) for $C_{33}H_{37}N_3O_7$: C 67.45; H 6.35; N 7.15. Found: C 67.63; H 6.39; N 7.21.

1,3-Dicyclohexyl-8-hydroxy-6-(2-hydroxyphenyl)-9-(4-methoxybenzoyl)-1,3,6-triazaspiro[4.4]non-8-ene-2,4,7-trione (1c)
Yield: 1.60 g (90%); white solid; mp 274–276 °C.

^{1}H NMR (400 MHz, DMSO-d_6): δ = 9.88 (s, 1 H), 7.77 (m, 2 H), 7.24 (m, 1 H), 7.05-6.98 (m, 3 H), 6.91 (m, 1 H), 6.81 (m, 1 H), 3.86-3.81 (m, 4 H), 3.06 (m, 1 H), 2.15-1.97 (m, 2 H), 1.83-1.70 (m, 5 H), 1.66-1.54 (m, 3 H), 1.49-1.41 (m, 2 H), 1.35-1.25 (m, 2 H), 1.21-1.08 (m, 5 H), 0.94 (m, 1 H) ppm.
^{13}C NMR (100 MHz, DMSO-d_6): δ = 186.9, 169.2, 163.2, 154.1, 153.9, 131.1 (2 C), 130.0, 129.8, 128.8, 128.1, 126.7, 120.1, 118.9, 116.6, 113.8, 113.5 (2 C), 80.7, 55.4, 52.1, 51.0, 30.0, 29.5, 28.7 (2 C), 28.6, 25.7, 25.2, 25.0, 24.8 (2 C) ppm.
IR (mineral oil): 3385, 3159, 1778, 1727, 1716, 1683 cm^{-1}.
Anal. Calcd (%) for $C_{32}H_{35}N_3O_7$: C 67.00; H 6.15; N 7.33. Found: C 66.78; H 6.50; N 7.36.

9-Benzoyl-6-(5-chloro-2-hydroxyphenyl)-1,3-dicyclohexyl-8-hydroxy-1,3,6-triazaspiro[4.4]non-8-ene-2,4,7-trione (1d)
Yield: 1.56 g (87%); white solid; mp 294–296 °C.
^{1}H NMR (400 MHz, DMSO-d_6): δ = 9.88 (s, 1 H), 7.74-7.68 (m, 4 H), 7.24 (m, 1 H), 6.99 (m, 1 H), 6.90 (m, 1 H), 6.81 (m, 1 H), 3.84 (m, 1 H), 3.09 (m, 1 H), 2.12-1.98 (m, 2 H), 1.84-1.58 (m, 8 H), 1.47 (m, 2 H), 1.36-1.05 (m, 7 H), 0.95 (m, 1 H) ppm.
^{13}C NMR (100 MHz, DMSO-d_6): δ = 188.5, 169.4, 163.3, 155.7, 153.9, 153.4, 137.3, 132.9, 129.9, 128.7 (2 C), 128.3 (2 C), 126.3, 121.9, 121.2, 118.2, 113.0, 80.6, 52.2, 51.1, 30.1, 29.5, 29.0, 28.7 (2 C), 25.7, 25.3, 25.0, 24.9 (2 C) ppm.
IR (mineral oil): 3340, 3190, 1782, 1726, 1706, 1672 cm^{-1}.
Anal. Calcd (%) for $C_{31}H_{32}ClN_3O_6$: C 64.41; H 5.58; N 7.27. Found: C 64.67; H 5.72; N 7.24.

1,3-Dicyclohexyl-8-hydroxy-6-(2-hydroxyphenyl)-9-(4-methylbenzoyl)-1,3,6-triazaspiro[4.4]non-8-ene-2,4,7-trione (1e)
Yield: 1.52 g (88%); white solid; mp 291–293 °C.
^{1}H NMR (400 MHz, DMSO-d_6): δ = 9.90 (s, 1 H), 7.66 (m, 2 H), 7.32 (m, 2 H), 7.24 (m, 1H), 7.00 (m, 1 H), 6.90 (m, 1 H), 6.81 (m, 1 H), 3.84 (m, 1 H), 3.07 (m, 1 H), 2.39 (s, 3 H), 2.15-1.96 (m, 2 H), 1.83-1.54 (m, 8 H), 1.47 (m, 2 H), 1.36-1.10 (m, 7 H), 0.94 (m, 1 H) ppm.
^{13}C NMR (100 MHz, DMSO-d_6): δ = 188.1, 169.3, 163.2, 154.1, 153.9, 143.4, 134.7, 130.0 (2 C), 128.8 (2 C), 128.1, 126.7, 120.1, 119.0, 116.7, 113.4, 80.7, 52.1, 51.0, 30.0, 29.5, 28.7 (2 C), 28.6, 25.7, 25.3, 25.2, 25.0, 24.8 (2 C), 21.1 ppm.
IR (mineral oil): 3382, 3182, 1779, 1727, 1715, 1682 cm^{-1}.
Anal. Calcd (%) for $C_{32}H_{35}N_3O_6$: C 68.92; H 6.33; N 7.54. Found: C 69.11; H 6.38; N 7.57.

1,3-Dicyclohexyl-8-hydroxy-6-(2-hydroxyphenyl)-9-(4-nitrobenzoyl)-1,3,6-triazaspiro[4.4]non-8-ene-2,4,7-trione (1f)
Yield: 1.70 g (93%); white solid; mp 279–281 °C.
^{1}H NMR (400 MHz, DMSO-d_6): δ = 9.91 (s, 1 H), 8.31 (m, 2 H), 7.96 (m, 2 H), 7.24 (m, 1H), 7.00 (m, 1 H), 6.91 (m, 1 H), 6.81 (m, 1 H), 3.84 (m, 1 H), 3.12 (m, 1 H), 2.16-1.95 (m, 2 H), 1.89-1.59 (m, 8 H), 1.49 (m, 2 H), 1.36-1.10 (m, 7 H), 0.95 (m, 1 H) ppm.
^{13}C NMR (100 MHz, DMSO-d_6): δ = 186.5, 169.5, 163.2, 158.8, 153.9, 149.4, 142.9, 129.8 (2 C), 128.8, 128.1, 126.7, 123.3 (2 C), 120.2, 118.9, 116.6, 111.4, 80.4, 52.0, 51.0, 30.0, 29.5, 28.7 (2 C), 25.7, 25.2, 25.2, 24.9 (2 C), 24.9 ppm.
IR (mineral oil): 3381, 3126, 1782, 1727, 1704, 1673 cm^{-1}.
Anal. Calcd (%) for $C_{31}H_{32}N_4O_8$: C 63.26; H 5.48; N 9.52. Found: C 63.29; H 5.50; N 9.53.

9-(4-Chlorobenzoyl)-1,3-dicyclohexyl-8-hydroxy-6-(2-hydroxyphenyl)-1,3,6-triazaspiro[4.4]non-8-ene-2,4,7-trione (1g)
Yield: 1.54 g (86%); white solid; mp 284–286 °C.
^{1}H NMR (400 MHz, DMSO-d_6): δ = 9.89 (s, 1 H), 7.77 (m, 2 H), 7.58 (m, 2 H), 7.24 (m, 1 H), 6.99 (m, 1 H), 6.90 (m, 1 H), 6.81 (m, 1 H), 3.84 (m, 1 H), 3.08 (m, 1 H), 2.15-1.95 (m, 2 H), 1.87-1.58 (m, 8 H), 1.47 (m, 2 H), 1.35-1.05 (m, 7 H), 0.94 (m, 1 H) ppm.
^{13}C NMR (100 MHz, DMSO-d_6): δ = 187.2, 169.2, 163.1, 156.3, 154.0, 153.8, 137.6, 136.1, 130.5 (2 C), 130.0, 128.4 (2 C), 126.7, 120.0, 118.9, 116.6, 112.6, 80.5, 52.0, 51.0, 30.0, 29.5, 28.7 (2 C), 25.7, 25.2, 25.2, 24.9, 24.8 (2 C) ppm.
IR (mineral oil): 3369, 3135, 1781, 1729, 1709, 1658 cm^{-1}.
Anal. Calcd (%) for $C_{31}H_{32}ClN_3O_6$: C 64.41; H 5.58; N 7.27. Found: C 64.52; H 5.64; N 7.39.

NMR charts of compounds 1a-g

MAN6046.001.esp

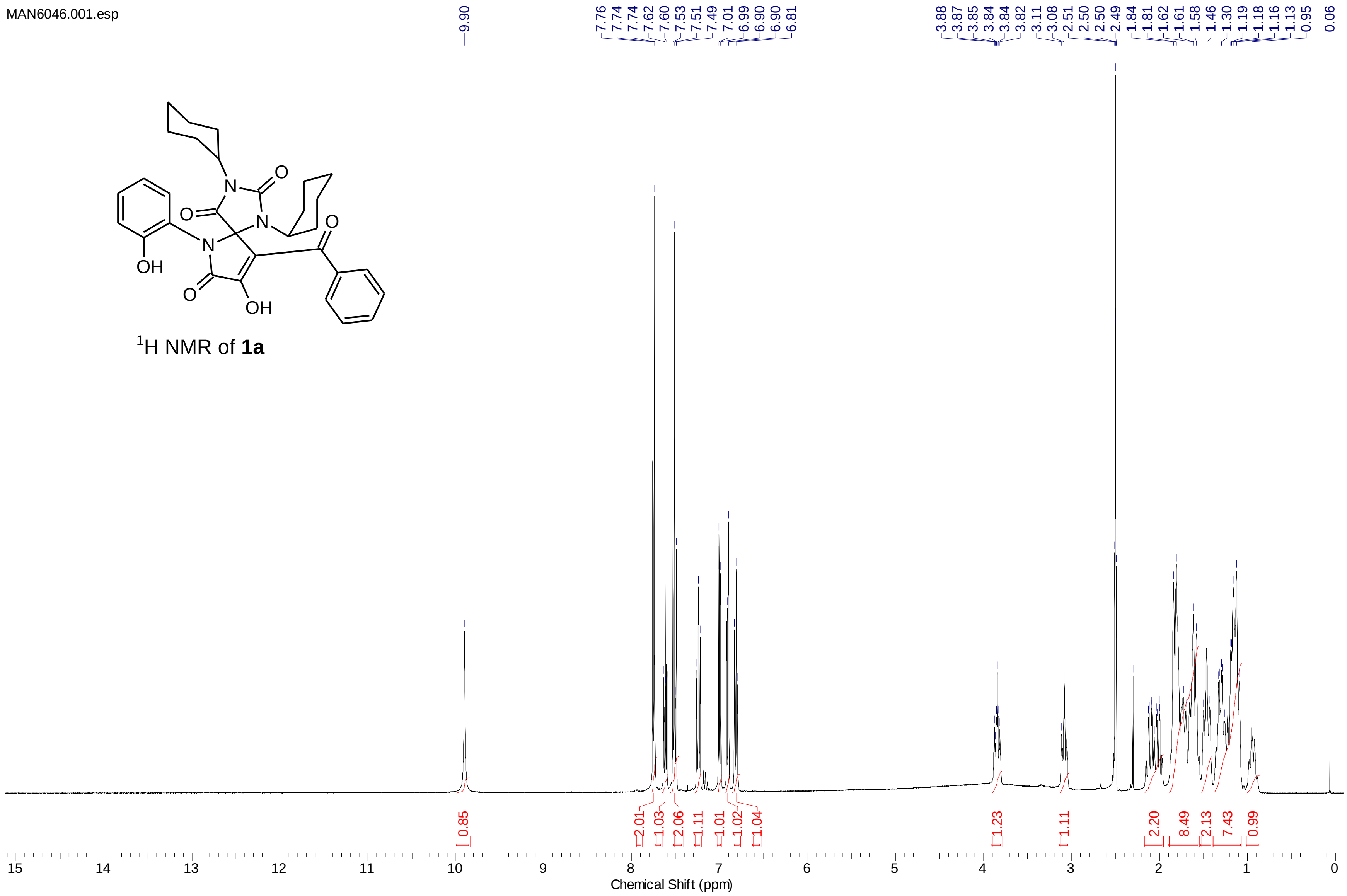

^{1}H NMR of **1a**

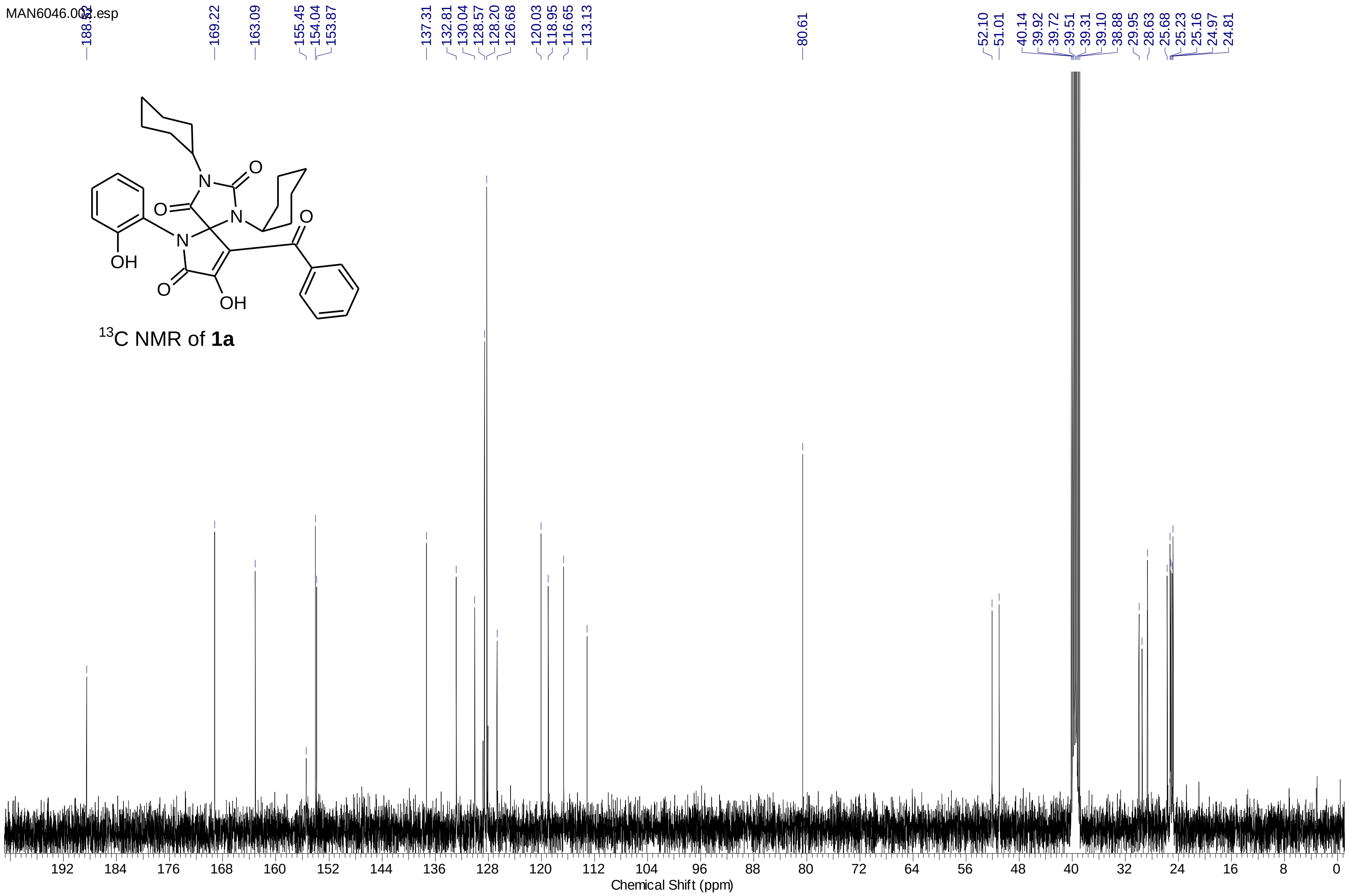
MAN6046.002.esp
169.22
163.09
155.45
154.04
153.87
137.31
132.81
130.04
128.57
128.20
126.68
120.03
118.95
116.65
113.13
80.61
52.10
51.01
40.14
39.92
39.72
39.51
39.31
39.10
38.88
29.95
28.63
25.68
25.23
25.16
24.97
24.81
O
N
N
O
N
OH
O
OH
O
192 184 176 168 160 152 144 136 128 120 112 104 96 88 80 72 64 56 48 40 32 24 16 8 0
Chemical Shift (ppm)

^{13}C NMR of **1a**

MAN6048.001.esp

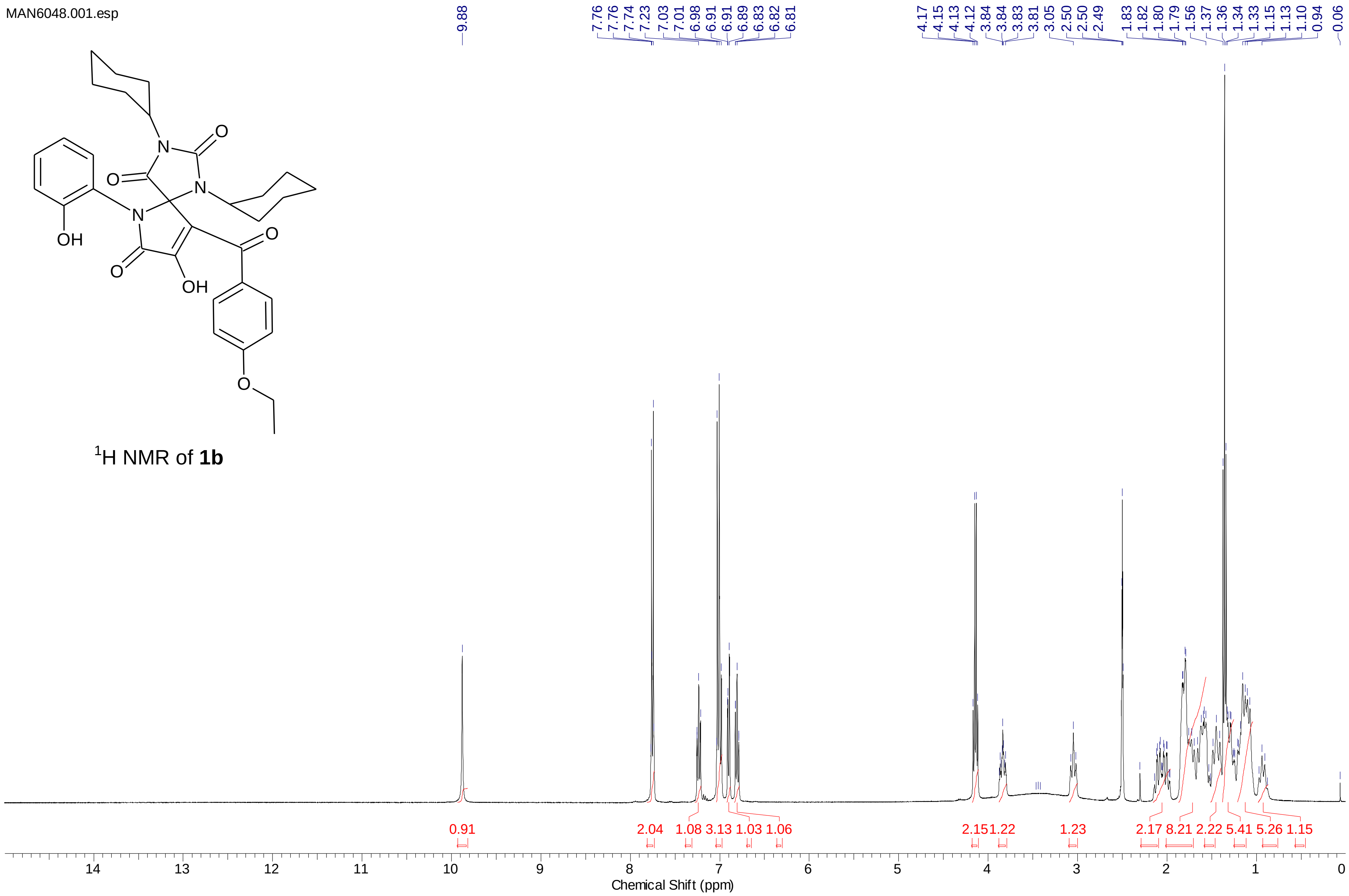

^{1}H NMR of **1b**

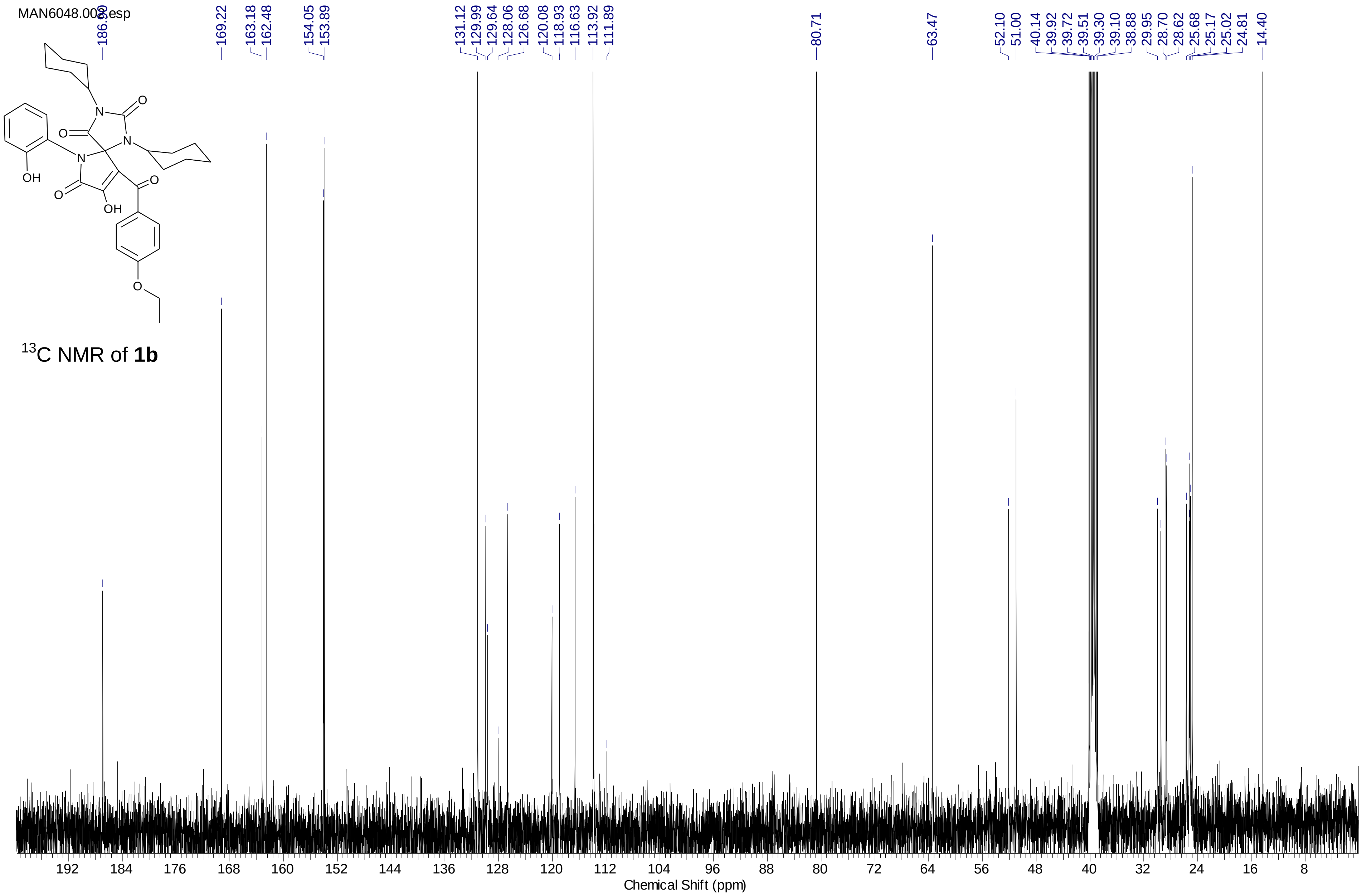
MAN6048.002.esp
186.90
169.22
163.18
162.48
154.05
153.89
131.12
129.99
129.64
128.06
126.68
120.08
118.93
116.63
113.92
111.89
80.71
63.47
52.10
51.00
40.14
39.92
39.72
39.51
39.30
39.10
38.88
29.95
28.70
28.62
25.68
25.17
25.02
24.81
14.40
N
O
O
N
N
OH
O
OH
O
O
192
184
176
168
160
152
144
136
128
120
112
104
96
88
80
72
64
56
48
40
32
24
16
8
Chemical Shift (ppm)

^{13}C NMR of **1b**

MAN6767.001.esp

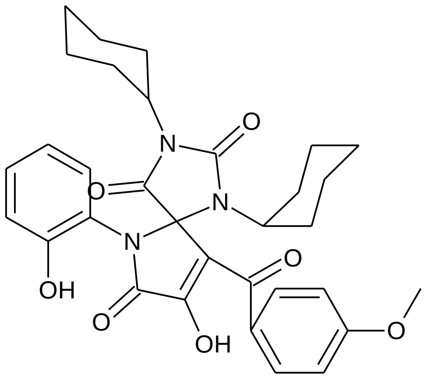

^{1}H NMR of **1c**

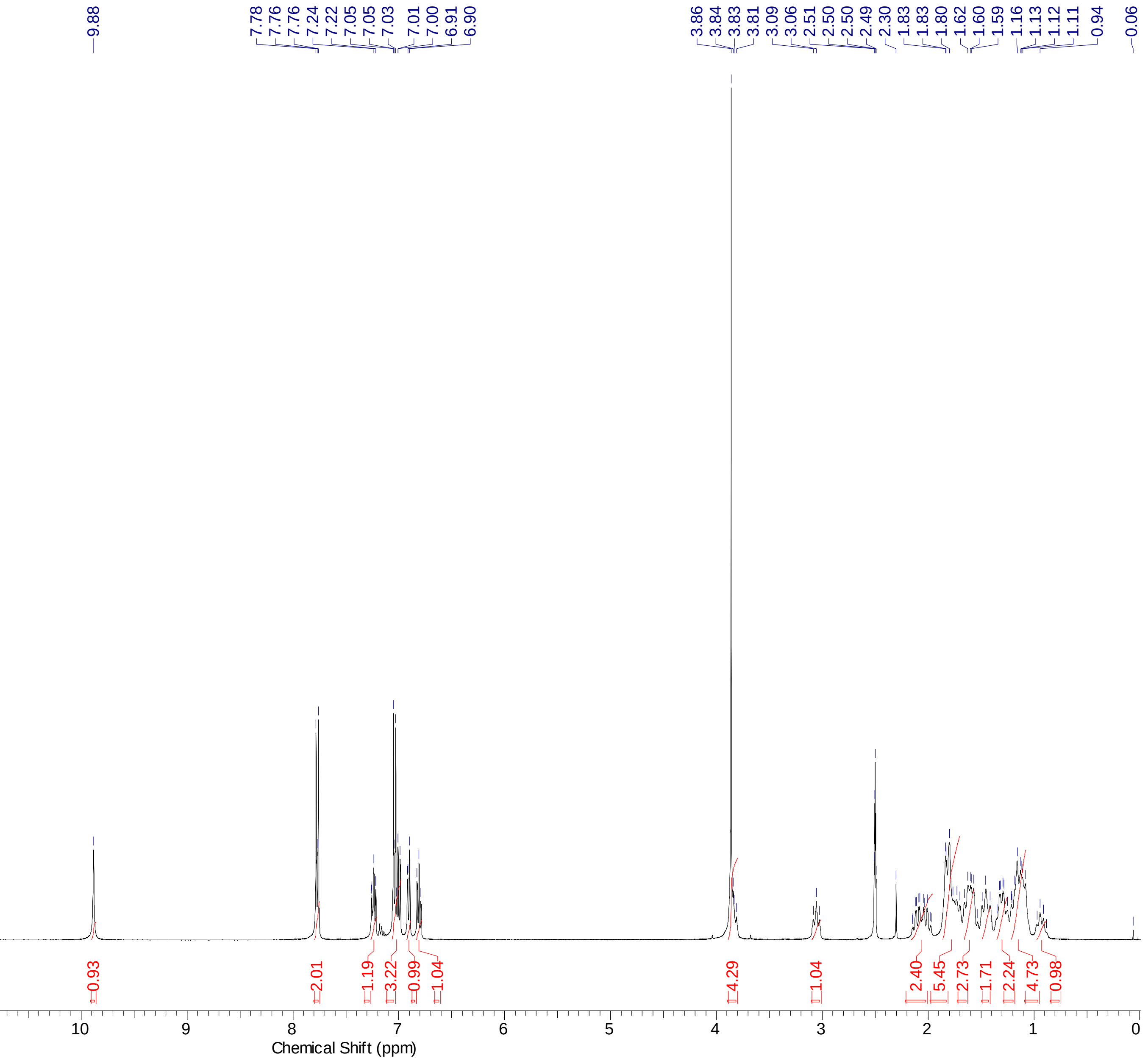

MAN6767.002.esp

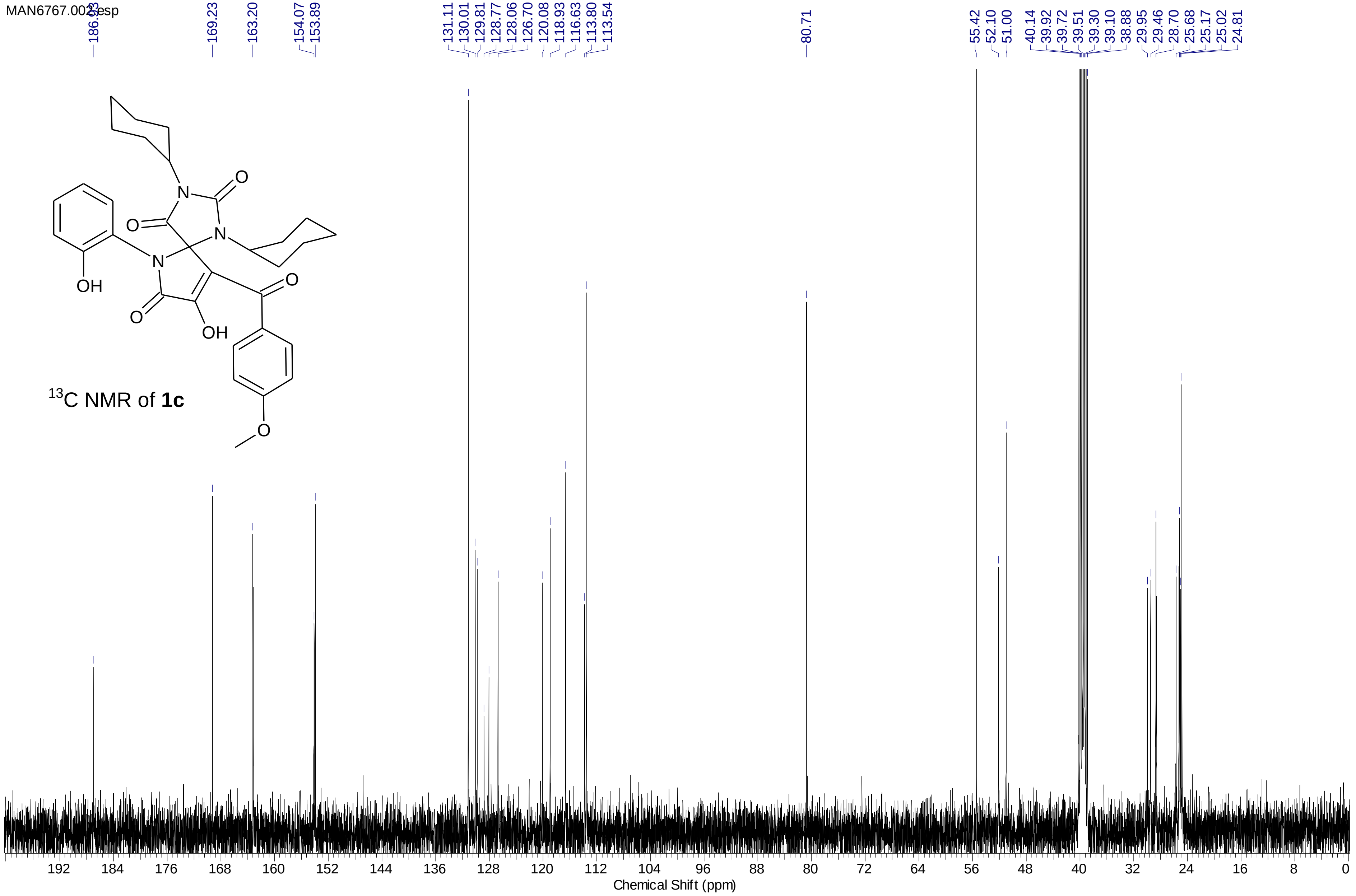

^{13}C NMR of **1c**

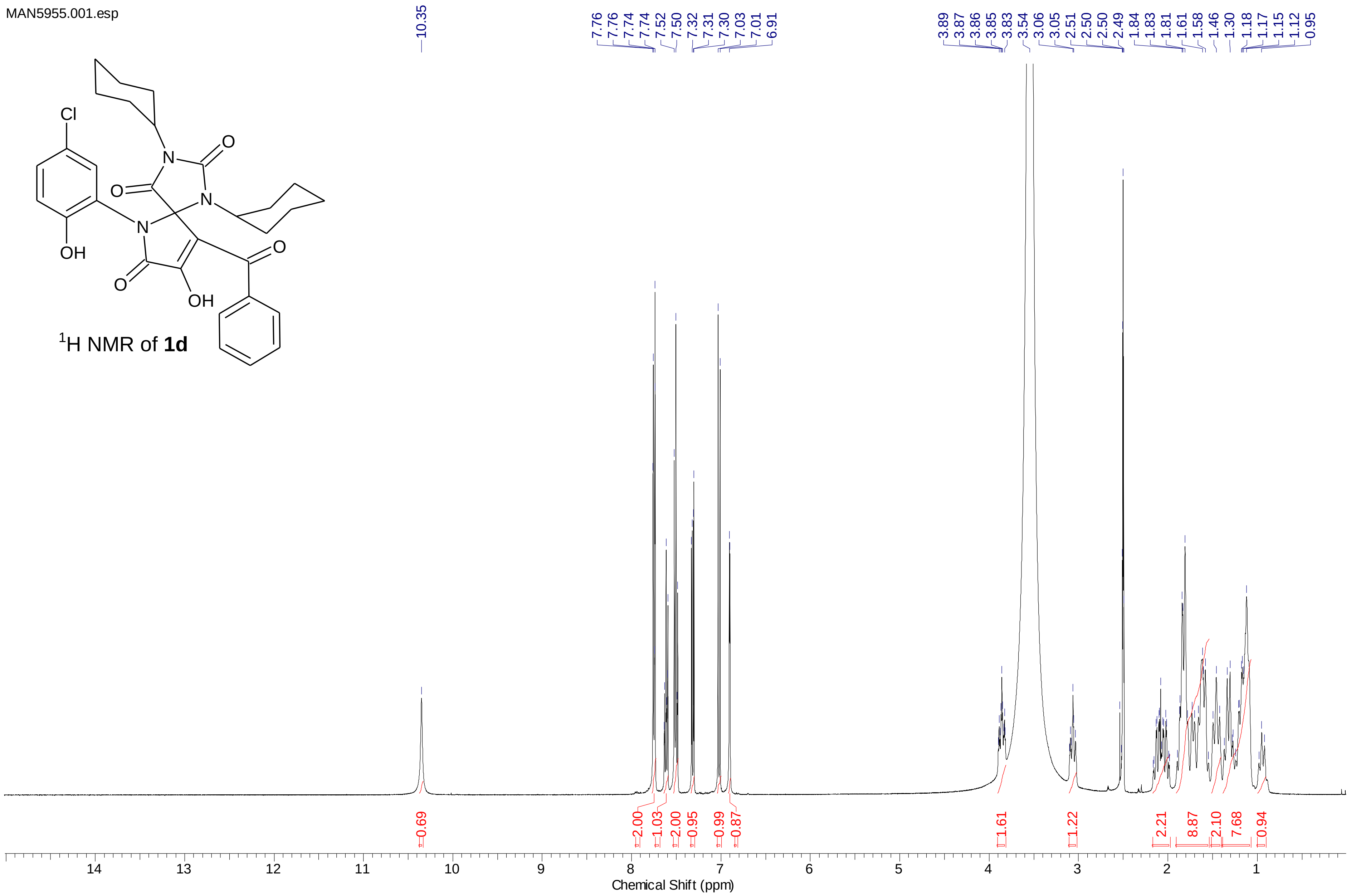
MAN5955.001.esp
10.35
7.76 7.76 7.74 7.74 7.52 7.50 7.32 7.31 7.30 7.03 7.01 6.91
3.89 3.87 3.86 3.85 3.83 3.54 3.06 3.05 2.51 2.50 2.50 2.49 1.84 1.83 1.81 1.61 1.58 1.46 1.30 1.18 1.17 1.15 1.12 0.95
Cl
O
N
N
O
OH
N
O
O
OH
0.69
2.00 1.03 2.00 0.95 0.99 0.87
1.61
1.22
2.21 8.87 2.10 7.68 0.94
14 13 12 11 10 9 8 7 6 5 4 3 2 1
Chemical Shift (ppm)

^{1}H NMR of **1d**

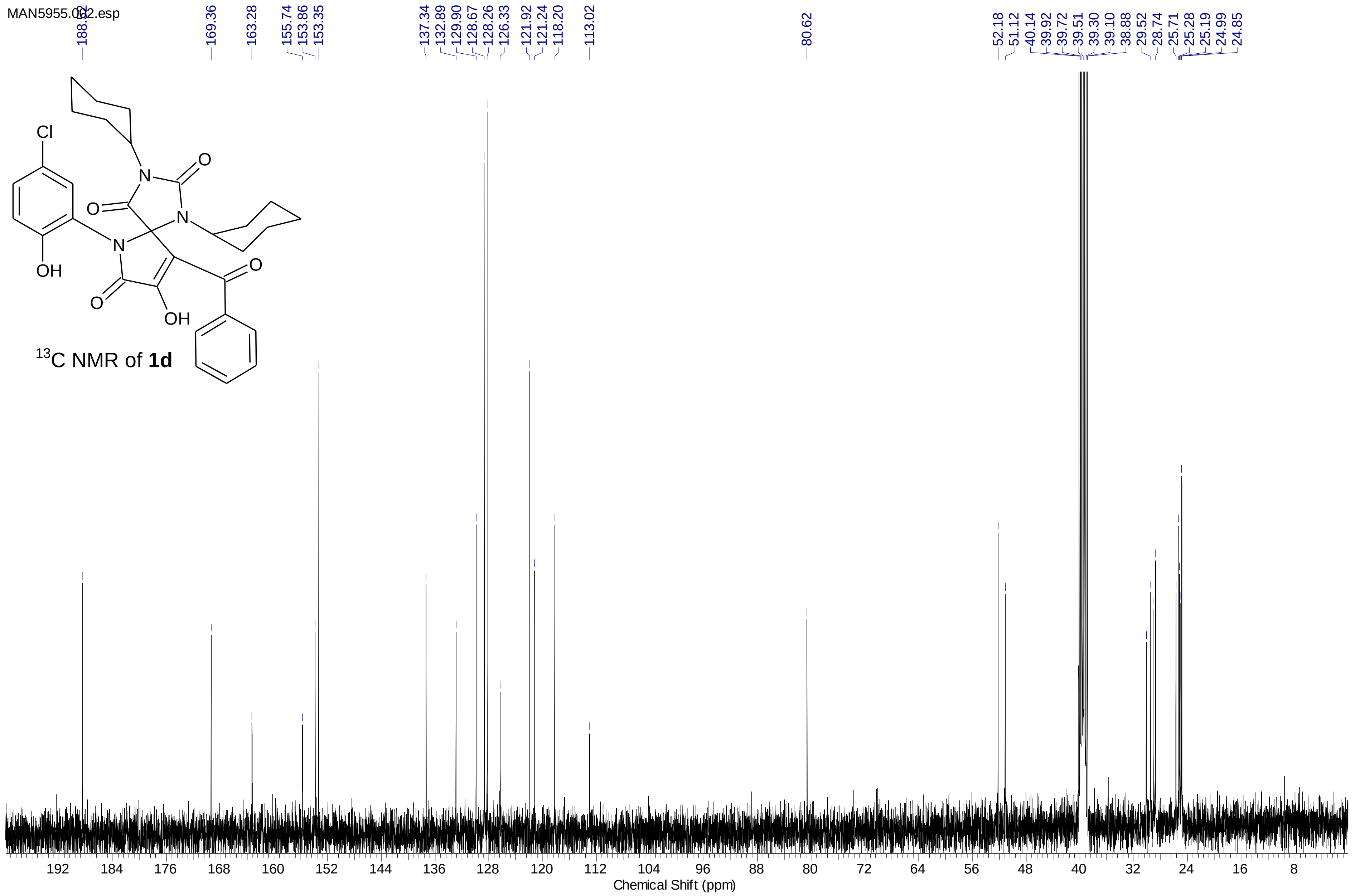
MAN5955.002.esp
188.52
169.36
163.28
155.74
153.86
153.35
137.34
132.89
129.90
128.67
128.26
126.33
121.92
121.24
118.20
113.02
80.62
52.18
51.12
40.14
39.92
39.72
39.51
39.30
39.10
38.88
29.52
28.74
25.71
25.28
25.19
24.99
24.85
Cl
O
N
N
O
N
OH
O
O
OH
^{13}C NMR of **1d**
192
184
176
168
160
152
144
136
128
120
112
104
96
88
80
72
64
56
48
40
32
24
16
8
Chemical Shift (ppm)

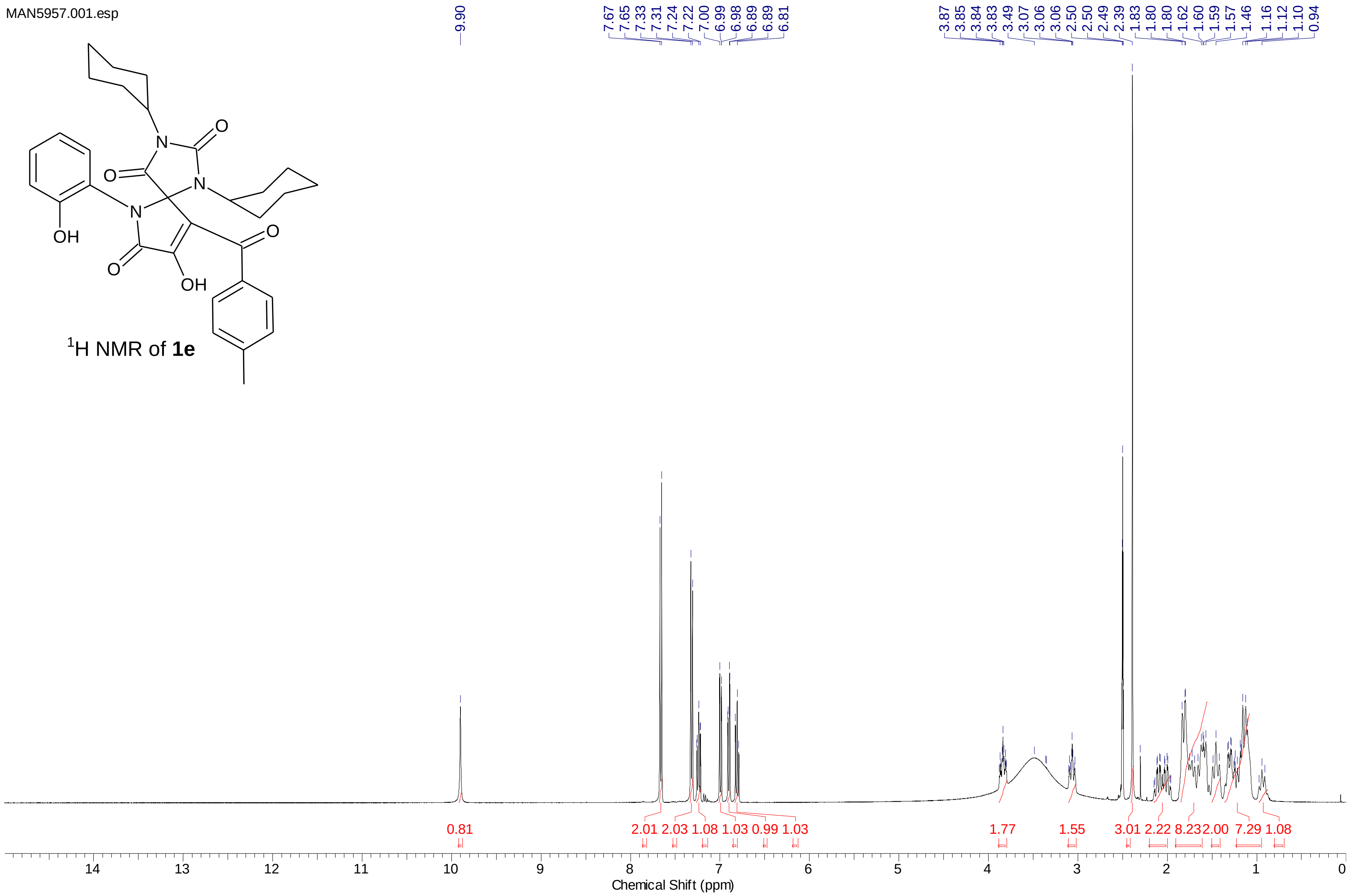
MAN5957.001.esp
9.90
7.67 7.65 7.33 7.31 7.24 7.22 7.00 6.99 6.98 6.89 6.89 6.81
3.87 3.85 3.84 3.83 3.49 3.07 3.06 3.06 2.50 2.50 2.49 2.39 1.83 1.80 1.80 1.62 1.60 1.59 1.57 1.46 1.16 1.12 1.10 0.94
0.81 2.01 2.03 1.08 1.03 0.99 1.03 1.77 1.55 3.01 2.22 8.23 2.00 7.29 1.08
14 13 12 11 10 9 8 7 6 5 4 3 2 1 0
Chemical Shift (ppm)
O
N
N
N
OH
OH
O
O
O

^{1}H NMR of **1e**

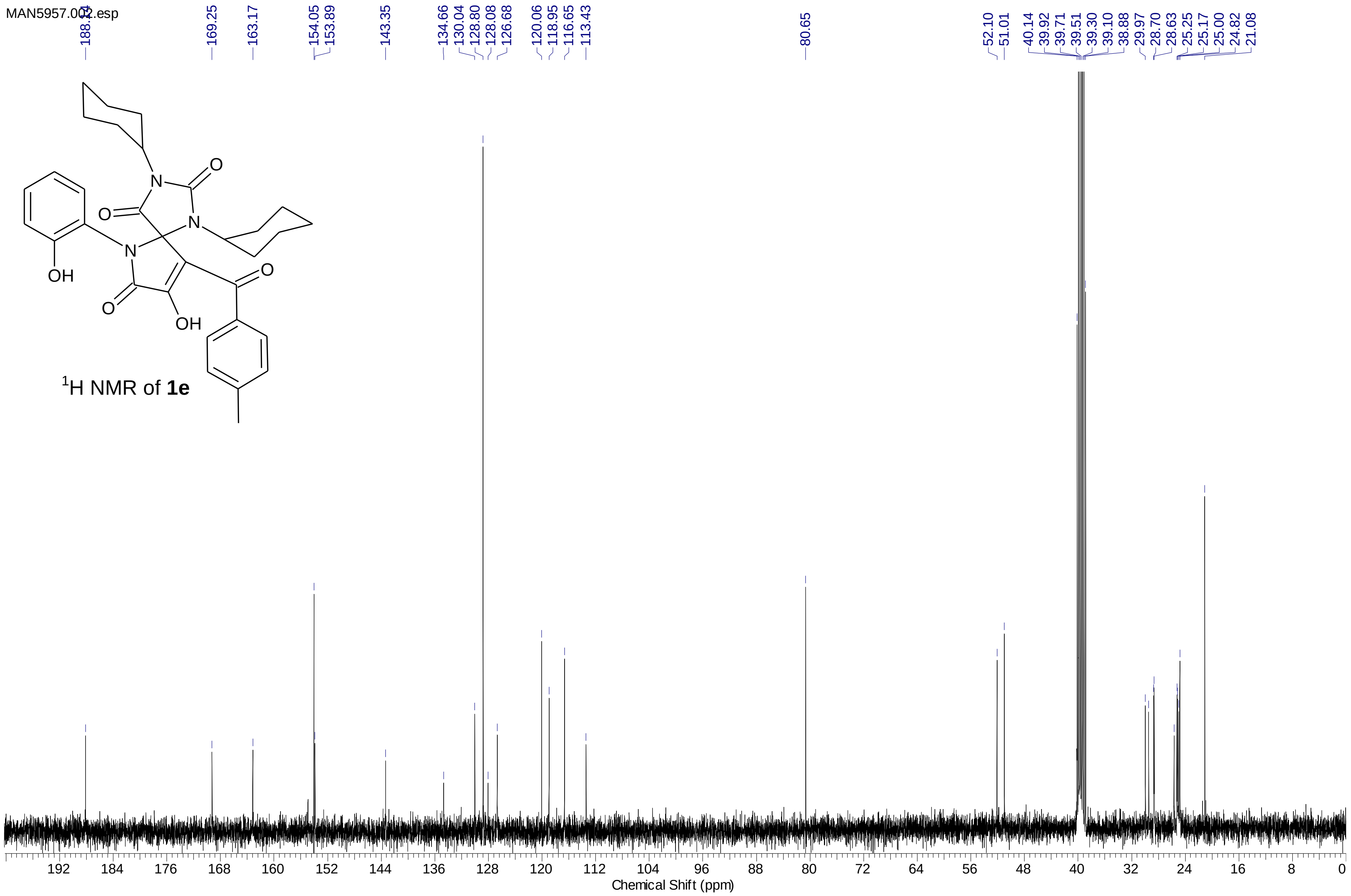
MAN5957.002.esp
188.14
169.25
163.17
154.05
153.89
143.35
134.66
130.04
128.80
128.08
126.68
120.06
118.95
116.65
113.43
80.65
52.10
51.01
40.14
39.92
39.71
39.51
39.30
39.10
38.88
29.97
28.70
28.63
25.25
25.17
25.00
24.82
21.08
O
N
O
N
N
OH
O
OH
O
192
184
176
168
160
152
144
136
128
120
112
104
96
88
80
72
64
56
48
40
32
24
16
8
0
Chemical Shift (ppm)

^{1}H NMR of **1e**

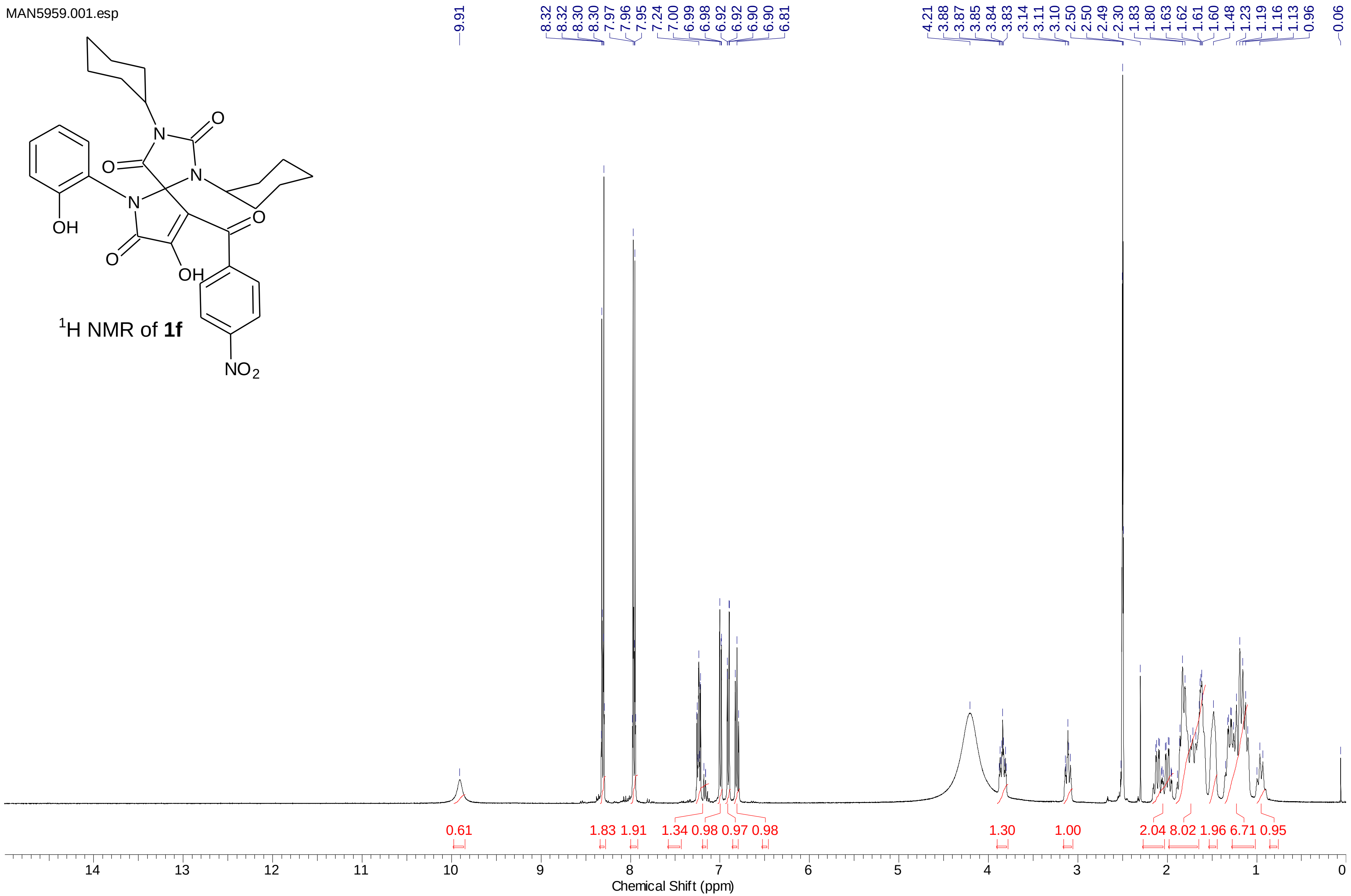
MAN5959.001.esp
9.91
8.32 8.32 8.30 8.30 7.97 7.96 7.95 7.24 7.00 6.99 6.98 6.92 6.92 6.90 6.81
4.21 3.88 3.87 3.85 3.84 3.83 3.14 3.11 3.10 2.50 2.50 2.49 2.30 1.83 1.80 1.63 1.62 1.61 1.60 1.48 1.23 1.19 1.16 1.13 0.96
0.06
OH
OH
O
O
O
O
O
N
N
N
NO2
0.61
1.83 1.91
1.34 0.98 0.97 0.98
1.30
1.00
2.04 8.02 1.96 6.71 0.95
14 13 12 11 10 9 8 7 6 5 4 3 2 1 0
Chemical Shift (ppm)

^{1}H NMR of **1f**

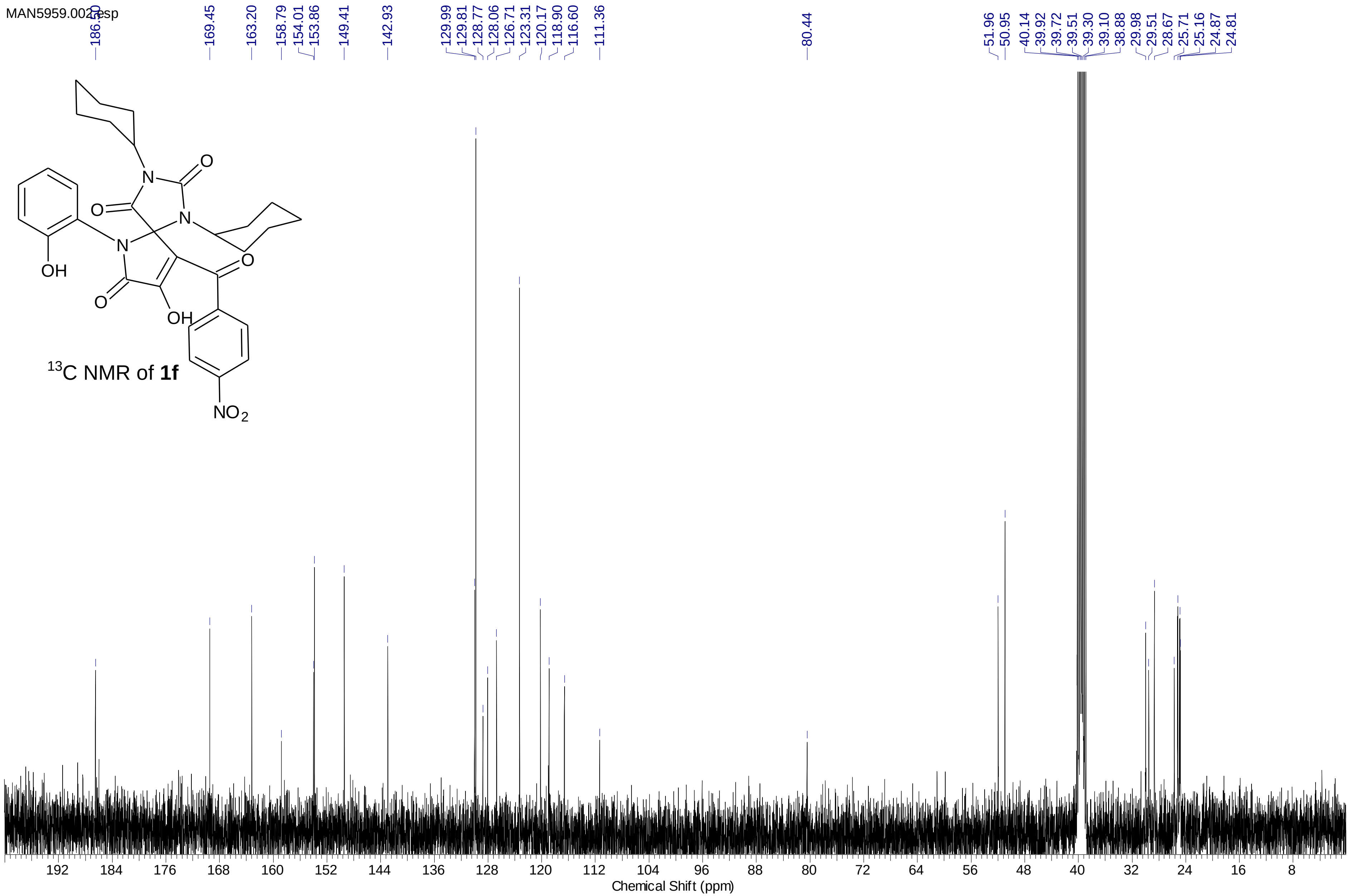
MAN5959.002.esp
186.50
169.45
163.20
158.79
154.01
153.86
149.41
142.93
129.99
129.81
128.77
128.06
126.71
123.31
120.17
118.90
116.60
111.36
80.44
51.96
50.95
40.14
39.92
39.72
39.51
39.30
39.10
38.88
29.98
29.51
28.67
25.71
25.16
24.87
24.81
N
O
OH
NO2
Chemical Shift (ppm)

^{13}C NMR of **1f**

MAN6047.001.esp

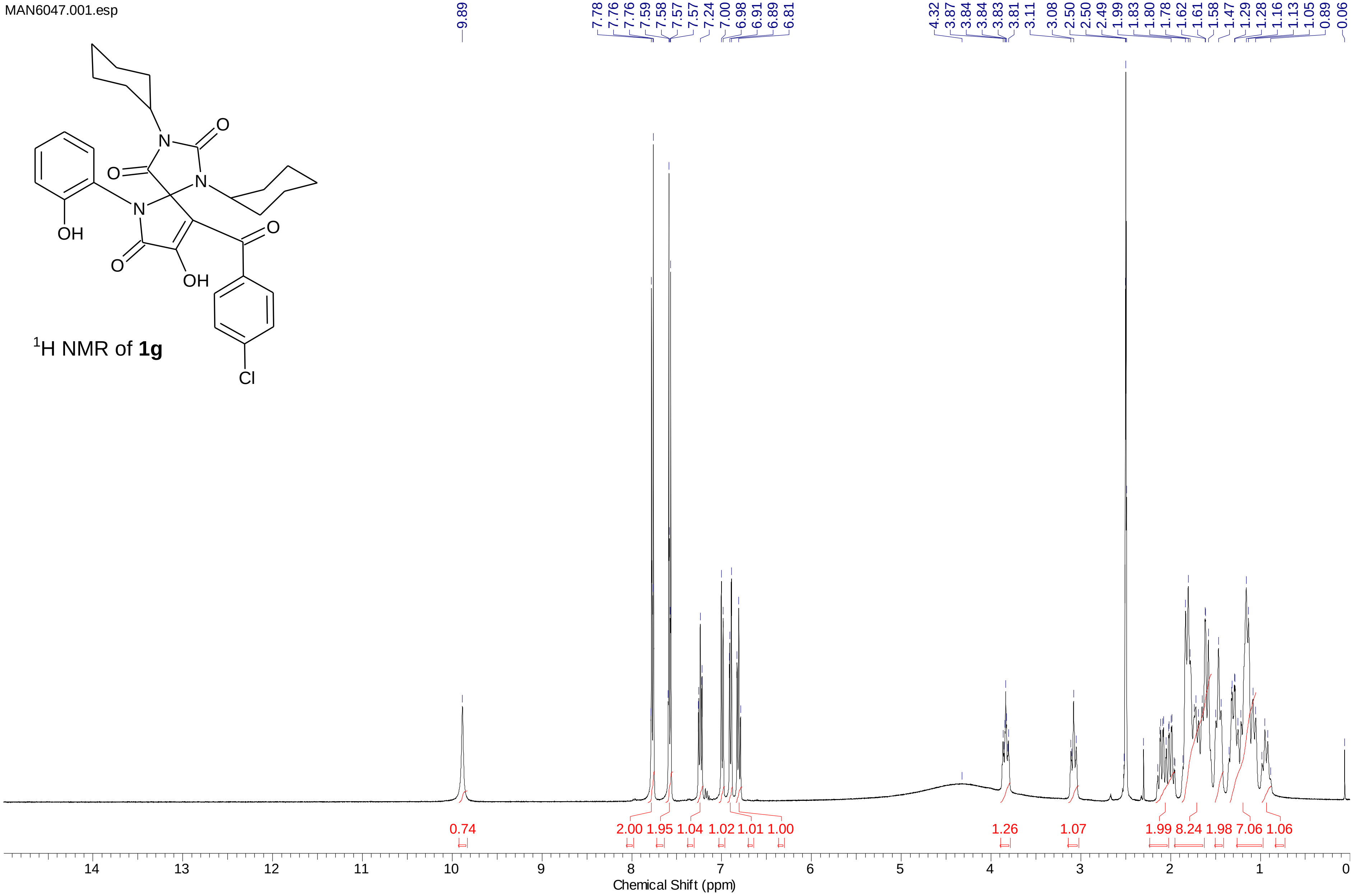

^{1}H NMR of **1g**

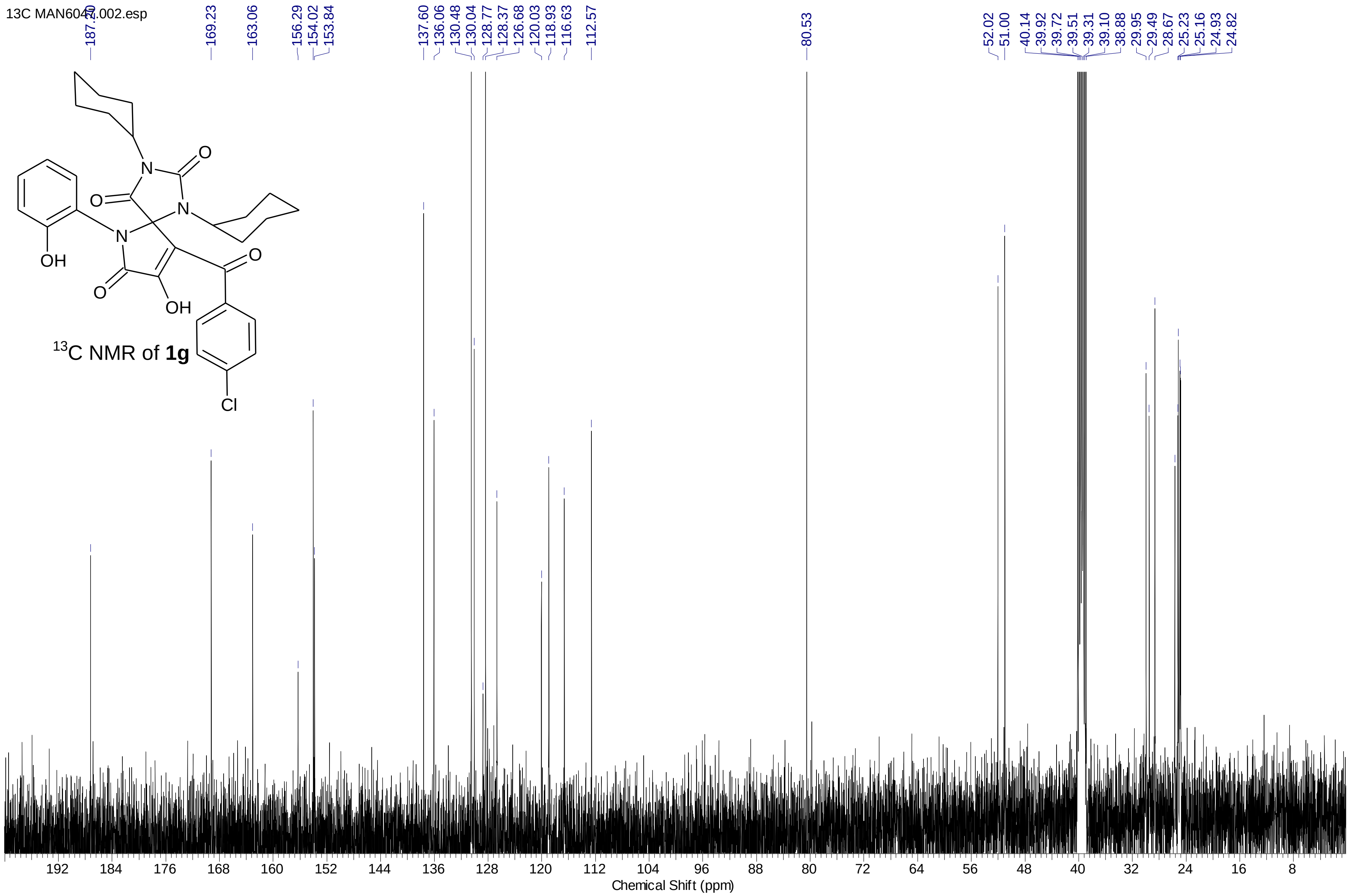

13C MAN6047.002.esp
187.70
169.23
163.06
156.29
154.02
153.84
137.60
136.06
130.48
130.04
128.77
128.37
126.68
120.03
118.93
116.63
112.57
80.53
52.02
51.00
40.14
39.92
39.72
39.51
39.31
39.10
38.88
29.95
29.49
28.67
25.23
25.16
24.93
24.82
^{13}C NMR of **1g**
Chemical Shift (ppm)

General procedure to compounds 4a-n

A suspension of the corresponding compound **1** (1.8 mmol) and the corresponding carbodiimide **5** (1.8 mmol for **5a**,**c**,**d**; 3.6 mmol for DIC **5b**) in 20 mL of toluene was refluxed for 2 h. Then the resulting precipitate was filtered off to afford the desired compound **4**.

9-Benzoyl-1,3-dicyclohexyl-1-(1,3-dicyclohexyl-6-(2-hydroxyphenyl)-2,4,7-trioxo-1,3,6-triazaspiro[4.4]non-8-en-8-yl)urea (4a)

Yield: 1.26 g (93%); white solid; mp 273–275 °C.

^{1}H NMR (400 MHz, $CDCl_3$): δ = 7.85 (m, 2 H), 7.62 (m, 1 H), 7.47 (m, 2 H), 7.19 (m, 1 H), 7.01 (m, 2 H), 6.88 (m, 1 H), 6.73 (br. s, 1 H), 4.85 (d, 1 H, *J* = 4.0 Hz), 4.03 (m, 1 H), 3.74 (m, 1 H), 3.46 (m, 1 H), 2.99 (m, 1 H), 2.21 (m, 2 H), 2.00-1.50 (m, 22 H), 1.44-0.97 (m, 16 H) ppm.

^{13}C NMR (100 MHz, $CDCl_3$): δ = 189.4, 168.7, 165.8, 154.5, 154.4, 152.7, 142.5, 137.9, 135.8, 134.2, 130.2, 129.0 (2 C), 128.7 (2 C), 125.6, 121.7, 121.1, 119.4, 82.1, 59.7, 54.6, 52.8, 49.9, 33.3, 33.0, 31.9, 30.4, 30.4, 30.1, 29.1, 29.0, 26.3, 26.1, 26.1, 29.9, 25.8, 25.7, 25.6, 25.3, 25.2, 25.0, 24.9, 24.7 ppm.

IR (mineral oil): 3411, 3132, 1781, 1730, 1652 cm^{-1}.

Anal. Calcd (%) for $C_{44}H_{55}N_5O_6$: C 70.47; H 7.39; N 9.34. Found: C 70.63; H 7.39; N 9.50.

1-(9-Benzoyl-1,3-dicyclohexyl-6-(2-hydroxyphenyl)-2,4,7-trioxo-1,3,6-triazaspiro[4.4]non-8-en-8-yl)-1,3-diisopropylurea (4b)

Yield: 1.06 g (88%); white solid; mp 231–232 °C.

^{1}H NMR (400 MHz, DMSO-d_6): δ = 9.96 (s, 1 H), 7.85 (m, 2 H), 7.68 (m, 1 H), 7.52 (m, 2 H), 7.23 (m, 1 H), 6.99 (m, 1 H) 6.91 (m, 1 H), 6.82 (m, 1 H), 5.78 (d, 1 H, *J* = 4.0 Hz), 4.08 (m, 1 H), 3.88 (m, 1 H), 3.63 (m, 1 H), 3.26 (s, 1 H), 3.05 (m, 1 H), 2.15-2.03 (m, 2 H), 1.81-1.57 (m, 7 H), 1.46 (m, 1 H), 1.36-1.28 (m, 3 H), 1.21-1.11 (m, 5 H), 1.06-0.85 (m, 13 H) ppm.

^{13}C NMR (100 MHz, DMSO-d_6): δ = 189.6, 168.4, 163.9, 154.2, 153.8, 153.6, 143.0, 136.6, 135.6, 134.1, 129.9, 128.6 (4 C), 126.6, 120.5, 118.9, 116.6, 81.3, 52.9, 51.5, 49.7, 42.1, 29.8, 29.6, 28.7, 28.5, 25.6, 25.2, 25.1, 25.0, 24.7, 24.5, 22.4, 22.3, 21.4, 19.8 ppm.

IR (mineral oil): 3404, 3171, 1771, 1747, 1711, 1671 cm^{-1}.

Anal. Calcd (%) for $C_{38}H_{47}N_5O_6$: C 68.14; H 7.07; N 10.46. Found: C 67.97; H 7.26; N 10.22.

1,3-Dicyclohexyl-1-(1,3-dicyclohexyl-9-(4-ethoxybenzoyl)-6-(2-hydroxyphenyl)-2,4,7-trioxo-1,3,6-triazaspiro[4.4]non-8-en-8-yl)urea (4c)

Yield: 1.30 g (91%); white solid; mp 192–194 °C.
^{1}H NMR (400 MHz, DMSO-d_6): δ = 9.99 (s, 1 H), 7.86 (m, 2 H), 7.22 (m, 1 H), 6.98 (m, 3 H), 6.88 (m, 1 H), 6.80 (m, 1 H) 5.91 (d, 1 H, *J* = 4.0 Hz), 4.20-4.08 (m, 2 H), 3.86 (m, 1 H), 3.67 (m, 1 H), 3.42 (s, 1 H), 2.99 (m, 1 H), 2.13-2.00 (m, 2 H), 1.84-1.45 (m, 18 H), 1.38-0.88 (m, 23 H) ppm.
^{13}C NMR (100 MHz, DMSO-d_6): δ = 187.8, 168.3, 164.2, 163.4, 154.3 (2 C), 153.6, 142.1, 139.2, 131.5 (2 C), 129.9, 128.5, 126.7, 120.7, 118.9, 116.6, 114.3 (2 C), 81.4, 63.7, 57.0, 53.0, 51.4, 49.2, 32.6 (2 C), 31.3, 30.3, 29.9, 29.6, 28.7, 28.7, 25.6, 25.3, 25.2 (2 C), 25.2, 24.9, 24.8, 24.7 (2 C), 24.7 (2 C), 24.6, 14.33 ppm.
IR (mineral oil): 3357, 3175, 1775, 1731, 1716, 1646 cm^{-1}.
Anal. Calcd (%) for $C_{46}H_{59}N_5O_7$: C 69.58; H 7.49; N 8.82. Found: C 69.71; H 7.45; N 8.89.

1,3-Dicyclohexyl-1-(1,3-dicyclohexyl-6-(2-hydroxyphenyl)-9-(4-methoxybenzoyl)-2,4,7-trioxo-1,3,6-triazaspiro[4.4]non-8-en-8-yl)urea (4d)
Yield: 1.28 g (91%); white solid; mp 230–232 °C.
^{1}H NMR (400 MHz, DMSO-d_6): δ = 9.96 (s, 1 H), 7.88 (m, 2 H), 7.22 (m, 1 H), 6.98 (m, 3 H), 6.89 (m, 1 H), 6.81 (m, 1 H), 5.87 (d, 1 H, *J* = 8.0 Hz), 3.91-3.84 (m, 4 H), 3.67 (m, 1 H), 3.43 (m, 1 H), 2.99 (m, 1 H), 2.11-2.01 (m, 2 H), 1.85-1.45 (m, 18 H), 1.39-0.86 (m, 20 H) ppm.
^{13}C NMR (100 MHz, DMSO-d_6): δ = 187.8, 168.2, 164.1, 164.0, 154.2 (2 C), 153.6, 142.1, 139.1, 131.4 (2 C), 129.8, 128.6, 126.6, 120.7, 118.9, 116.5, 113.9 (2 C), 81.36, 57.0, 55.6, 53.0, 51.4, 49.2, 32.5 (2 C), 32.5, 31.2, 30.2, 29.8, 29.6, 28.7, 28.6, 25.6, 25.3, 25.2 (2 C), 25.1, 24.8, 24.7, 24.7, 24.6 (2 C), 24.5 ppm.
IR (mineral oil): 3341, 3176, 1779, 1732, 1716, 1645 cm^{-1}.
Anal. Calcd (%) for $C_{45}H_{57}N_5O_7$: C 69.30; H 7.37; N 8.98. Found: C 69.53; H 7.45; N 8.89.

1-(9-Benzoyl-6-(5-chloro-2-hydroxyphenyl)-1,3-dicyclohexyl-2,4,7-trioxo-1,3,6-triazaspiro[4.4]non-8-en-8-yl)-1,3-dicyclohexylurea (4e)
Yield: 1.33 g (94%); white solid; mp 258–260 °C.
^{1}H NMR (400 MHz, DMSO-d_6): δ = 10.47 (s, 1 H), 7.88 (m, 2 H), 7.69 (m, 1 H), 7.50 (m, 2 H), 7.31 (m, 1 H), 7.01 (m, 1 H) 6.91 (m, 1 H), 6.00 (d, 1 H, *J* = 8.0 Hz), 3.89 (m, 1 H), 3.63 (m, 1 H), 3.53 (m, 1 H), 3.01 (m, 1 H), 2.15-2.02 (m, 2 H), 1.84-1.45 (m, 18 H), 1.37-0.83 (m, 20 H) ppm.

^{13}C NMR (100 MHz, DMSO-d_6): δ = 189.7, 168.3, 164.1, 154.2, 153.6 (2 C), 143.1, 137.7, 135.7, 134.5, 129.8, 128.9 (2 C), 128.7 (2 C), 126.4, 121.9, 121.7, 118.2, 81.3, 57.5, 53.1, 51.6, 49.4, 32.6 (2 C), 32.5, 31.3, 30.3, 30.1, 29.7, 29.0, 28.7, 25.7, 25.4 (2 C), 25.3 (2 C), 25.2, 25.0, 24.9, 24.8 (2 C), 24.6 ppm.
IR (mineral oil): 3412, 3198, 1782, 1727, 1657 cm^{-1}.
Anal. Calcd (%) for $C_{44}H_{54}ClN_5O_6$: C 67.37; H 6.94; N 8.93. Found: C 67.43; H 6.88; N 9.02.

1,3-Dicyclohexyl-1-(1,3-dicyclohexyl-6-(2-hydroxyphenyl)-9-(4-methylbenzoyl)-2,4,7-trioxo-1,3,6-triazaspiro[4.4]non-8-en-8-yl)urea (4f)
Yield: 1.22 g (89%); white solid; mp 245–253 °C.
^{1}H NMR (400 MHz, DMSO-d_6): δ = 10.00 (s, 1 H), 7.77 (m, 2 H), 7.30 (m, 2 H), 7.22 (m, 1 H), 6.98 (m, 1 H), 6.89 (m, 1 H) 6.81 (m, 1 H), 5.84 (d, 1 H, J = 8.0 Hz), 3.87 (m, 1 H), 3.67 (m, 1 H), 3.38 (m, 1 H), 3.00 (m, 1 H), 2.39 (s, 3 H), 2.14-2.00 (m, 2 H), 1.84-1.45 (m, 18 H), 1.41-0.85 (m, 20 H) ppm.
^{13}C NMR (100 MHz, DMSO-d_6): δ = 189.2, 168.3, 164.0, 154.2, 154.1, 153.6, 145.0, 142.7, 138.3, 133.3 (2 C), 129.9, 129.2 (2 C), 128.9, 126.7, 120.6, 119.0, 116.6, 81.4, 59.6, 57.2, 53.0, 51.4, 49.2, 32.6, 32.5, 31.2, 30.2, 29.9, 29.6, 28.7, 28.6, 25.6, 25.4, 25.2 (2 C), 25.2, 25.1, 24.8, 24.8, 24.7, 24.6 (2 C), 21.3 ppm.
IR (mineral oil): 3336, 3164, 1778, 1734, 1715, 1650 cm^{-1}.
Anal. Calcd (%) for $C_{45}H_{57}N_5O_6$: C 70.75; H 7.52; N 9.17. Found: C 70.91; H 7.62; N 9.24.

1,3-Dicyclohexyl-1-(1,3-dicyclohexyl-6-(2-hydroxyphenyl)-9-(4-nitrobenzoyl)-2,4,7-trioxo-1,3,6-triazaspiro[4.4]non-8-en-8-yl)urea (4g)
Yield: 1.27 g (89%); white solid; mp 229–231 °C.
^{1}H NMR (400 MHz, DMSO-d_6): δ = 10.10 (s, 1 H), 8.33 (m, 2 H), 8.01 (m, 2 H), 7.24 (m, 1 H), 6.99 (m, 1 H), 6.90 (m, 1 H) 6.83 (m, 1 H), 5.99 (d, 1 H, J = 8.0 Hz), 3.87 (m, 1 H), 3.73 (m, 1 H), 3.49 (m, 1 H), 3.06 (m, 1 H), 2.14-1.97 (m, 2 H), 1.81-1.41 (m, 22 H), 1.36-0.88 (m, 16 H) ppm.
^{13}C NMR (100 MHz, DMSO-d_6): δ = 188.6, 168.4, 163.8, 154.2, 154.1, 153.6, 150.2, 144.9, 140.6, 138.3, 130.2, 129.9 (2 C), 126.8, 123.9 (2 C), 120.4, 119.1, 116.7, 81.1, 57.8, 52.9, 51.6, 49.5, 32.5, 32.3 (2 C), 29.9, 29.8, 28.7, 28.7, 25.7, 25.6, 25.4, 25.3, 25.2 (2 C), 25.0, 24.9 (2 C), 24.7, 24.7 (2 C), 24.6 ppm.
IR (mineral oil): 3336, 3194, 1779, 1731, 1716, 1656 cm^{-1}.

Anal. Calcd (%) for $C_{44}H_{54}N_6O_8$: C 66.48; H 6.85; N 10.57. Found: C 66.35; H 6.89; N 10.44.

1-(9-(4-Chlorobenzoyl)-1,3-dicyclohexyl-6-(2-hydroxyphenyl)-2,4,7-trioxo-1,3,6-triazaspiro[4.4]non-8-en-8-yl)-1,3-dicyclohexylurea (4h)

Yield: 1.31 g (93%); white solid; mp 203–204 °C.

^{1}H NMR (400 MHz, DMSO-d_6): δ = 9.98 (s, 1 H), 7.86 (m, 2 H), 7.58 (m, 2 H), 7.23 (m, 1 H), 6.99 (m, 1 H), 6.90 (m, 1 H), 6.81 (m, 1 H), 5.94 (d, 1 H, *J* = 4.0 Hz), 3.87 (m, 1 H), 3.73 (m, 1 H), 3.33 (m, 1 H), 3.03 (m, 1 H), 2.14-1.99 (m, 2 H), 1.82-1.40 (m, 20 H), 1.34-0.87 (m, 18 H) ppm.

^{13}C NMR (100 MHz, DMSO-d_6): δ = 188.5, 168.3, 163.8, 154.2, 154.0, 153.5, 143.7, 139.0, 134.4, 130.4, 129.9, 128.8 (2 C), 126.7, 120.5 (2 C), 118.9, 116.6, 81.1, 57.4, 52.9, 51.4, 49.3, 32.5, 32.3 (2 C), 31.2, 30.0, 29.8, 29.6, 28.6, 28.6 (2 C), 25.6, 25.4, 25.2 (2 C), 25.2, 25.1, 25.0, 24.8, 24.6 (2 C), 24.6 ppm.

IR (mineral oil): 3345, 3181, 1780, 1731, 1718, 1655 cm^{-1}.

Anal. Calcd (%) for $C_{44}H_{54}ClN_5O_6$: C 67.37; H 6.94; N 8.93. Found: C 67.76; H 7.11; N 9.02.

1-(1,3-Dicyclohexyl-6-(2-hydroxyphenyl)-9-(4-methylbenzoyl)-2,4,7-trioxo-1,3,6-triazaspiro[4.4]non-8-en-8-yl)-1,3-diisopropylurea (4i)

Yield: 1.05 g (85%); yellow solid; mp 158–160 °C.

^{1}H NMR (400 MHz, DMSO-d_6): δ = 9.96 (s, 1 H), 7.76 (m, 2 H), 7.32 (m, 2 H), 7.23 (m, 1 H), 6.99 (m, 1 H) 6.90 (m, 1 H), 6.82 (m, 1 H), 5.73 (d, 1 H, *J* = 8.0 Hz), 4.08 (m, 1 H), 3.87 (m, 1 H), 3.64 (m, 1 H), 3.26 (s, 1 H), 3.03 (m, 1 H), 2.39 (s, 3 H), 2.14-2.03 (m, 2 H), 1.85-1.53 (m, 7 H), 1.47 (m, 1 H), 1.37-1.28 (m, 3 H), 1.20-1.11 (m, 5 H), 1.04-0.86 (m, 13 H) ppm.

^{13}C NMR (100 MHz, DMSO-d_6): δ = 189.1, 168.4, 164.0, 154.1, 153.8, 153.6, 144.8, 142.5, 139.7, 133.1, 130.0, 129.2 (2 C), 128.8 (2 C), 126.5, 120.6, 118.9, 116.5, 81.3, 52.9, 51.5, 49.6, 42.1, 29.8, 29.6, 28.7, 28.5, 25.6, 25.2, 25.1, 25.0, 24.7, 24.5, 22.4, 22.3, 21.4, 21.17, 19.8 ppm.

IR (mineral oil): 3324, 3180, 1799, 1748, 1693, 1669, 1644 cm^{-1}.

Anal. Calcd (%) for $C_{39}H_{49}N_5O_6$: C 68.50; H 7.22; N 10.24. Found: C 68.65; H 7.26; N 10.30.

1-(1,3-Dicyclohexyl-6-(2-hydroxyphenyl)-9-(4-methoxybenzoyl)-2,4,7-trioxo-1,3,6-triazaspiro[4.4]non-8-en-8-yl)-1,3-diisopropylurea (4j)

Yield: 1.08 g (86%); white solid; mp 150–152 °C.

^{1}H NMR (400 MHz, $CDCl_3$): δ = 7.86 (m, 2 H), 7.18 (m, 1 H), 7.01 (m, 3 H), 6.94 (m, 2 H), 6.86 (m, 1 H), 4.88 (d, 1 H, J = 8.0 Hz), 4.22 (m, 1 H), 4.02 (m, 1 H), 3.88 (s, 3 H), 3.76 (m, 1 H), 2.97 (m, 1 H), 2.27-2.14 (m, 2 H), 2.01 (m, 1 H), 1.92-1.69 (m, 6 H), 1.60 (m, 1 H), 1.51-1.47 (m, 2 H), 1.44-0.88 (m, 20 H) ppm.

^{13}C NMR (100 MHz, $CDCl_3$): δ = 187.5, 168.8, 165.8, 164.7, 154.5, 154.4, 152.8, 141.5, 138.6, 131.6 (2 C), 130.2, 128.5, 125.8, 121.6, 120.9, 119.1, 114.1 (2 C), 82.3, 55.5, 54.6, 52.8, 51.2, 43.1, 30.4, 30.2, 29.2, 28.9, 26.1, 25.9, 25.9 (2 C), 25.2, 25.0, 23.0, 22.6, 22.0, 20.2 ppm.

IR (mineral oil): 3421, 3198, 1716, 1686, 1664, 1649 cm^{-1}.

Anal. Calcd (%) for $C_{39}H_{49}N_5O_7$: C 66.93; H 7.06; N 10.01. Found: C 67.02; H 7.00; N 10.05.

1-(9-(4-Chlorobenzoyl)-1,3-dicyclohexyl-6-(2-hydroxyphenyl)-2,4,7-trioxo-1,3,6-triazaspiro[4.4]non-8-en-8-yl)-1,3-diisopropylurea (4k)

Yield: 1.12 g (88%); white solid; mp 154–157 °C.

^{1}H NMR (400 MHz, $CDCl_3$): δ = 7.80 (m, 2 H), 7.46 (m, 2 H), 7.19 (m, 1 H), 6.99 (m, 2 H), 6.87 (m, 1 H), 4.87 (d, 1 H, *J* = 4.0 Hz), 4.20 (m, 1 H), 4.02 (m, 1 H), 3.72 (m, 1 H), 2.96 (m, 1 H), 2.20 (m, 2 H), 2.00 (m, 1 H), 1.92-1.70 (m, 6 H), 1.63-1.62 (m, 1 H), 1.51-1.49 (m, 2 H), 1.44-1.23 (m, 6 H), 1.18-1.00 (m, 14 H) ppm.

^{13}C NMR (100 MHz, $CDCl_3$): δ = 188.1, 168.7, 165.4, 154.5, 154.3, 152.8, 142.7, 141.0, 137.2, 134.0, 130.3 (3 C), 129.2 (2 C), 125.7, 121.3, 121.0, 119.0, 82.1, 54.6, 52.8, 51.3, 43.2, 30.4, 30.3, 29.1, 28.9, 26.1, 25.9, 25.8, 25.2, 25.0, 23.4, 23.0, 22.6, 21.9, 20.2 ppm.

IR (mineral oil): 3173, 3083, 1702, 1681, 1666 cm^{-1}.

Anal. Calcd (%) for $C_{38}H_{46}ClN_5O_6$: C 68.81; H 6.58; N 9.94. Found: C 68.85; H 6.63; N 9.89.

9-Benzoyl-1-(1,3-dicyclohexyl-6-(2-hydroxyphenyl)-2,4,7-trioxo-1,3,6-triazaspiro[4.4]non-8-en-8-yl)-1,3-diphenylurea (4l)

Yield: 1.14 g (86%); pale yellow solid; mp 148–150 °C.

^{1}H NMR (400 MHz, $CDCl_3$): δ = 7.69 (m, 2 H), 7.55 (m, 1 H), 7.39 (m, 2 H), 7.27–7.17 (m, 4 H), 7.16–7.03 (m, 7 H), 6.88 (m, 2 H), 6.80–6.67 (m, 3 H), 3.97 (m, 1 H), 3.12 (m, 1 H), 2.14 (m, 2 H), 2.96–0.95 (m, 18 H) ppm.

^{13}C NMR (100 MHz, $CDCl_3$): δ = 188.1, 168.1, 164.5, 154.4, 152.7, 152.3, 144.2, 140.4, 137.1, 136.6, 134.4, 134.1, 130.3, 129.9 (2 C), 129.0 (2 C), 128.9, 128.7 (2 C), 127.0, 125.8, 125.1 (2 C), 124.5, 121.9, 121.8, 121.2, 120.0 (2 C), 119.8, 82.0, 54.4, 52.7, 30.5, 30.2, 29.0 (2 C), 26.1, 26.0, 25.8, 25.7, 25.1, 25.1 ppm.

IR (mineral oil): 3169, 1778, 1721, 1647 cm^{-1}.
Anal. Calcd (%) for $C_{44}H_{43}N_5O_6$: C 71.62; H 5.87; N 9.49. Found: C 71.89; H 5.91; N 9.55.

1-(1,3-Dicyclohexyl-6-(2-hydroxyphenyl)-2,4,7-trioxo-1,3,6-triazaspiro[4.4]non-8-en-8-yl)-9-(4-ethoxybenzoyl)-1,3-diphenylurea (4m)

Yield: 1.21 g (86%); yellow solid; mp 161–163 °C.
^{1}H NMR (400 MHz, $CDCl_3$): δ = 7.72 (m, 2 H), 7.32–7.05 (m, 11 H), 6.94–6.85 (m, 3 H), 6.78 (m, 2 H), 6.71 (m, 1 H), 4.09 (q, *J* 6.8 Hz, 2 H), 3.98 (m, 1 H), 3.10 (m, 1 H), 2.16 (m, 2 H), 1.91–0.99 (m, 21 H) ppm.
^{13}C NMR (100 MHz, $CDCl_3$): δ = 186.2, 168.1, 164.6, 164.1, 154.4, 152.6, 152.3, 143.4, 140.4, 137.2, 135.8, 131.4 (2 C), 130.2, 129.8 (2 C), 129.3, 129.0 (2 C), 127.1, 125.7, 125.3 (2 C), 124.4, 122.0, 121.3, 120.0 (3 C), 114.5 (2 C), 82.0, 64.0, 54.5, 52.7, 30.5, 30.2, 29.0 (2 C), 26.1, 25.9, 25.8 (2 C), 25.1 (2 C), 14.5 ppm.
IR (mineral oil): 3327, 3183, 1778, 1721, 1644 cm^{-1}.
Anal. Calcd (%) for $C_{46}H_{47}N_5O_7$: C 70.66; H 6.06; N 8.96. Found: C 70.34; H 6.14; N 9.05.

1,3-Dibutyl-1-(1,3-dicyclohexyl-6-(2-hydroxyphenyl)-2,4,7-trioxo-1,3,6-triazaspiro[4.4]non-8-en-8-yl)-9-(4-ethoxybenzoyl)urea (4n)

To precipitate compound **4n**, hexane (50 mL) was added to the reaction mixture.
Yield: 1.19 g (89%); pale yellow solid; mp 138–140 °C.
^{1}H NMR (400 MHz, $CDCl_3$): δ = 7.71 (m, 2 H), 7.20 (m, 1 H), 7.03–6.85 (m, 5 H), 5.05 (m, 1 H), 4.11 (q, *J* 6.8 Hz, 2 H), 4.05–3.97 (m, 1 H), 3.71–3.64 (m, 1 H), 3.51–3.44 (m, 1 H), 3.05–2.97 (m, 1 H), 2.95–2.84 (m, 2 H), 2.19 (m, 2 H), 2.05–0.87 (m, 35 H) ppm.
^{13}C NMR (100 MHz, $CDCl_3$): δ = 187.0, 169.0, 165.4, 164.1, 155.1, 154.4, 152.6, 142.6, 132.4, 131.0 (2 C), 130.3, 128.0, 125.5, 121.8, 121.2, 119.5, 114.7 (2 C), 82.1, 63.9, 54.6, 52.8, 48.7, 40.7, 31.4 (2 C), 31.3, 30.4, 30.1, 29.1, 28.9, 26.1, 25.9, 25.8, 25.2, 25.0, 20.1, 20.0, 14.6, 13.8 (2 C) ppm.
IR (mineral oil): 3538, 3271, 1780, 1724, 1642 cm^{-1}.
Anal. Calcd (%) for $C_{42}H_{55}N_5O_7$: C 67.99; H 7.47; N 9.44. Found: C 67.64; H 7.53; N 9.31.

NMR charts of compounds 4a-n

MAN3761.001.esp

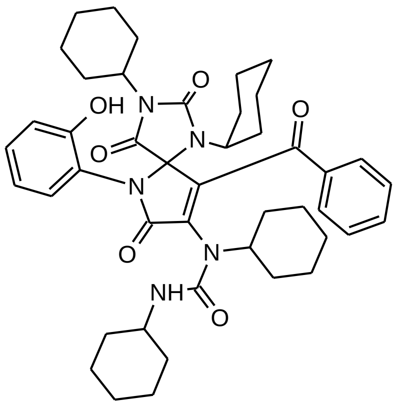

^{1}H NMR of **4a**

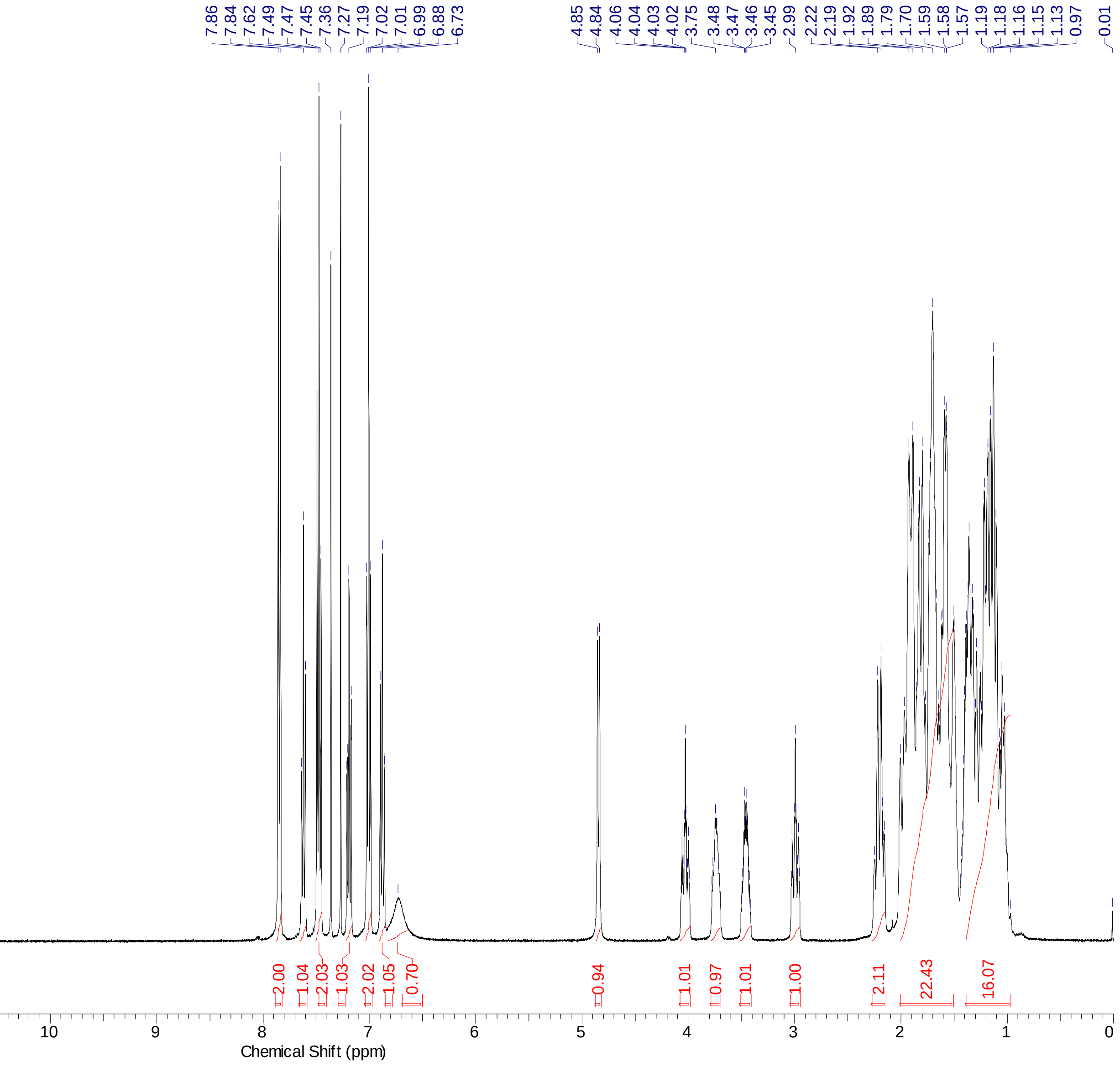

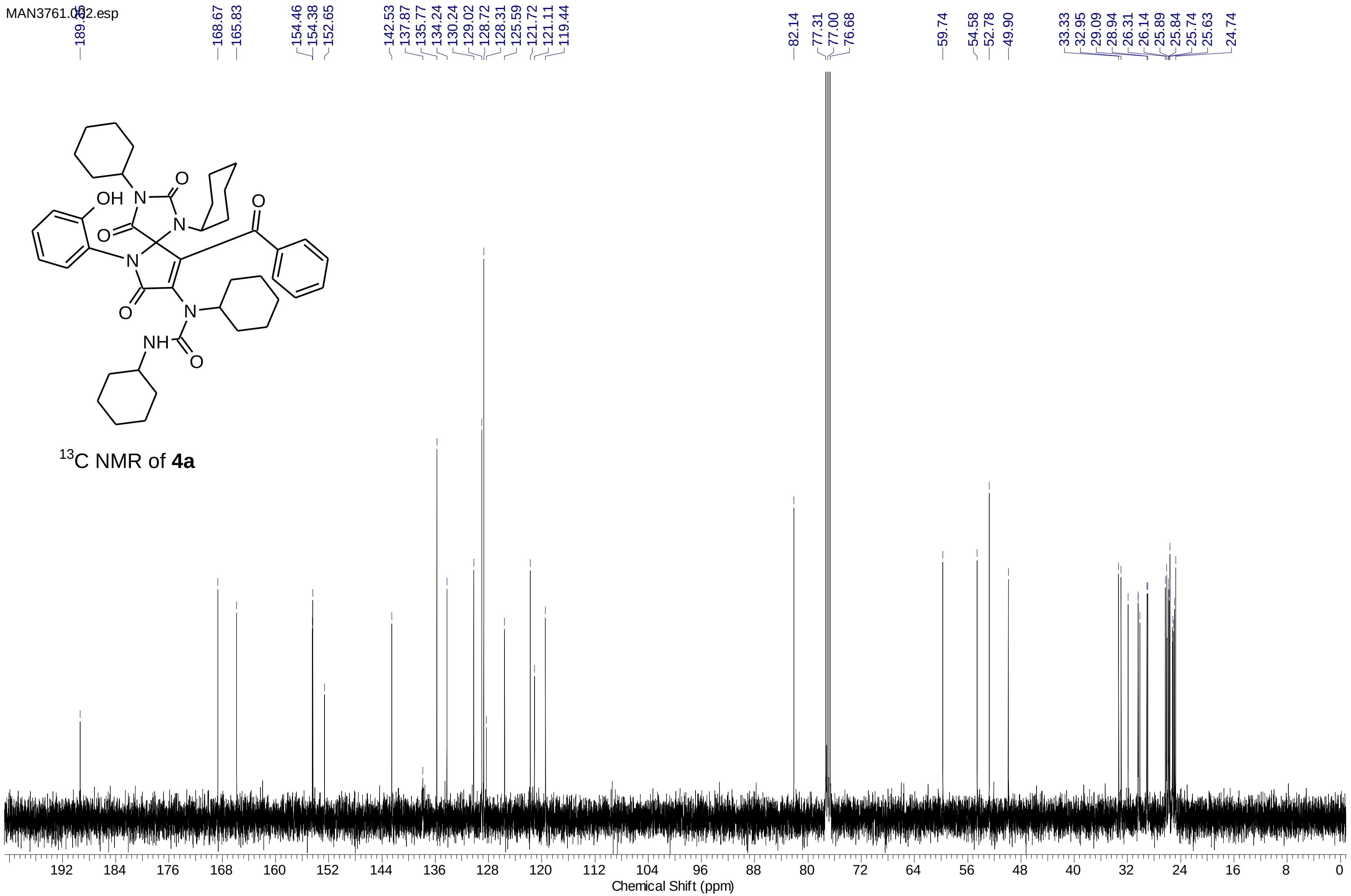

MAN3761.002.esp
189.35
168.67
165.83
154.46
154.38
152.65
142.53
137.87
135.77
134.24
130.24
129.02
128.72
128.31
125.59
121.72
121.11
119.44
82.14
77.31
77.00
76.68
59.74
54.58
52.78
49.90
33.33
32.95
29.09
28.94
26.31
26.14
25.89
25.84
25.74
25.63
24.74
OH
N
O
N
O
N
O
N
O
NH
O
192 184 176 168 160 152 144 136 128 120 112 104 96 88 80 72 64 56 48 40 32 24 16 8 0
Chemical Shift (ppm)

^{13}C NMR of **4a**

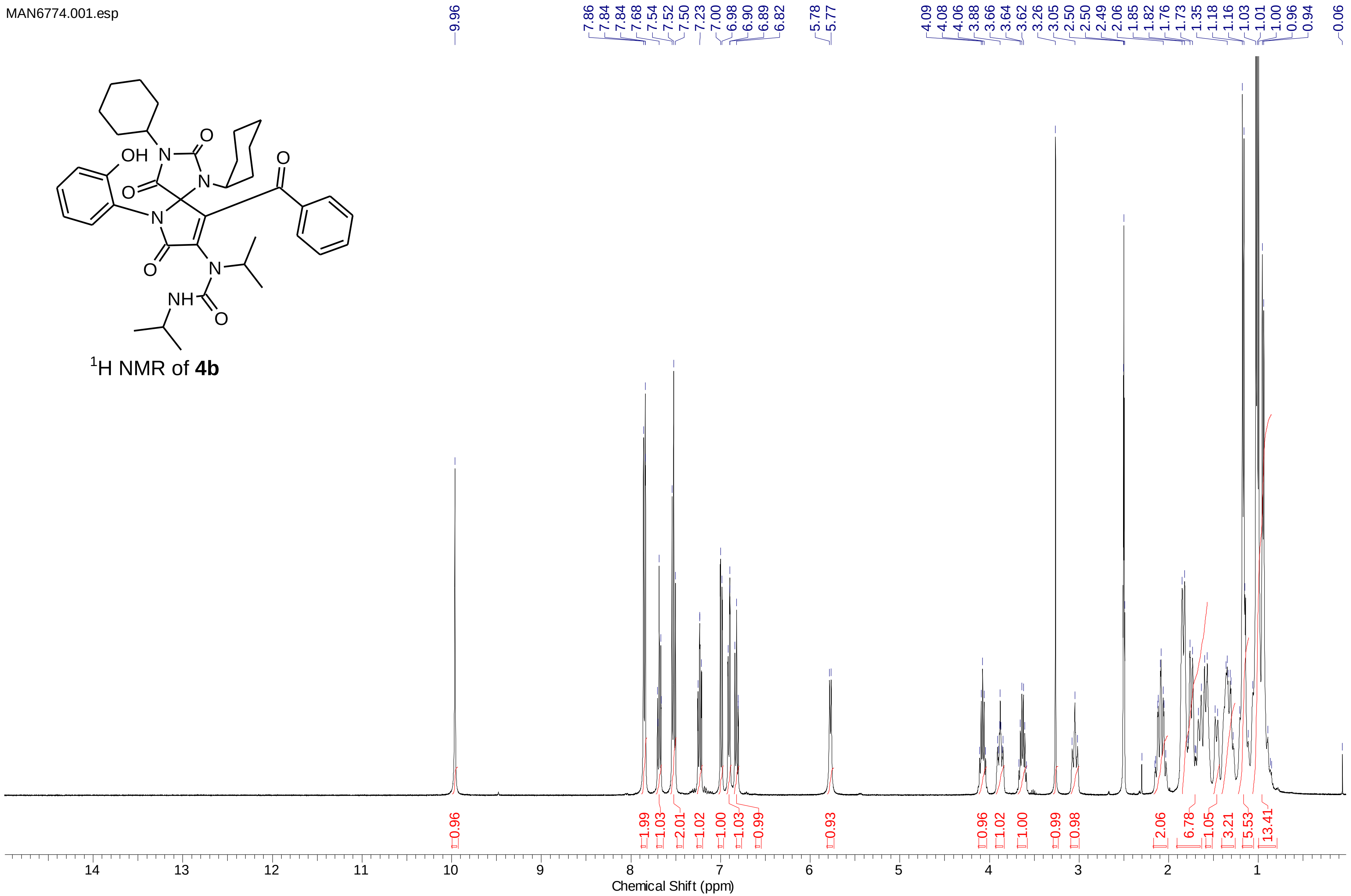
MAN6774.001.esp
9.96
7.86 7.84 7.84 7.68 7.54 7.52 7.50 7.23 7.00 6.98 6.90 6.89 6.82
5.78 5.77
4.09 4.08 4.06 3.88 3.66 3.64 3.62 3.26 3.05 2.50 2.49 2.06 1.85 1.82 1.76 1.73 1.35 1.18 1.16 1.03 1.01 1.00 0.96 0.94
0.06
0.96
1.99 1.03 2.01 1.02 1.00 1.03 0.99
0.93
0.96 1.02 1.00 0.99 0.98
2.06 6.78 1.05 3.21 5.53 13.41
14 13 12 11 10 9 8 7 6 5 4 3 2 1
Chemical Shift (ppm)
OH
N
O
N
O
O
N
O
N
NH
O

^{1}H NMR of **4b**

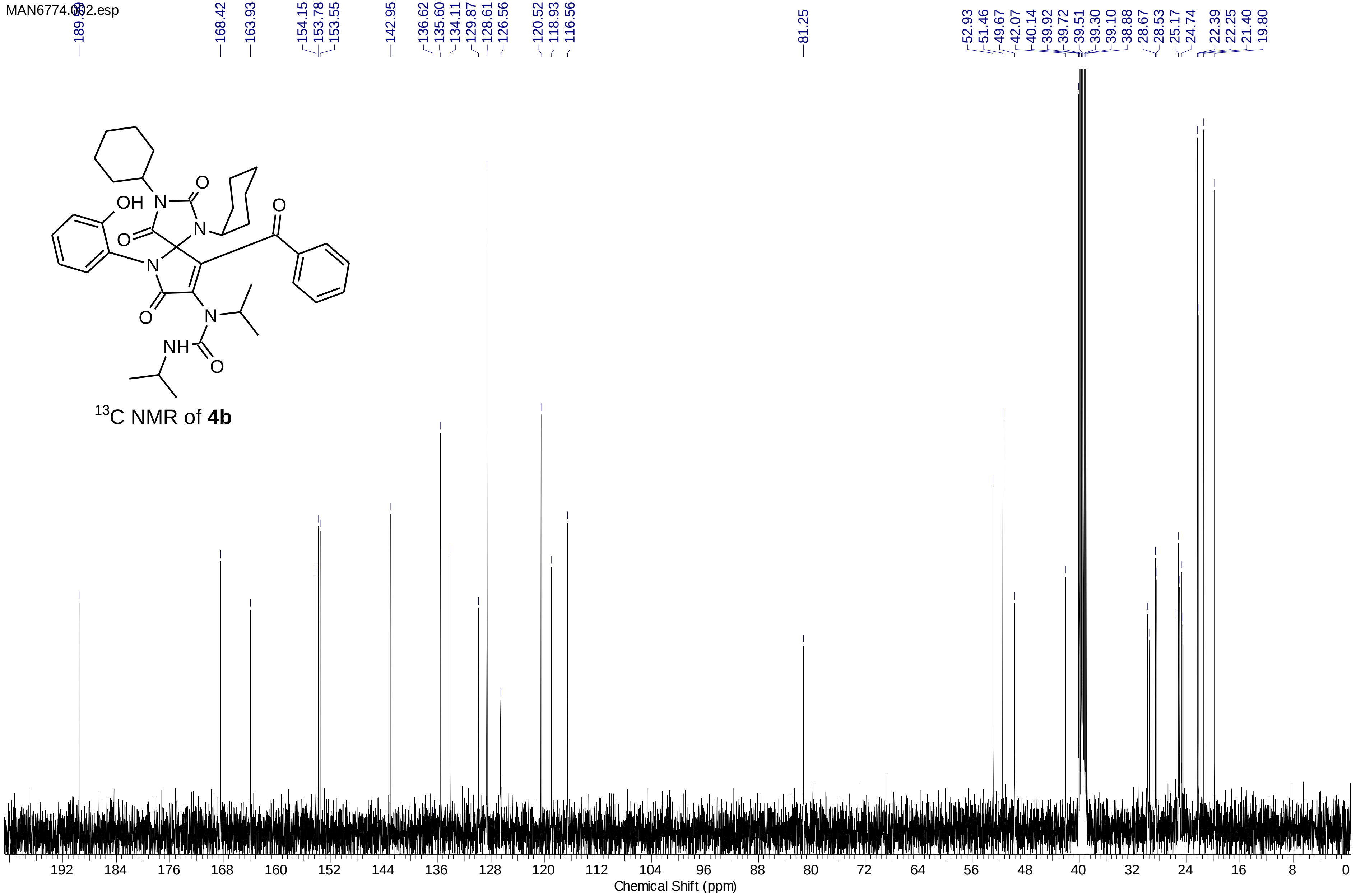
MAN6774.002.esp
189.59
168.42
163.93
154.15
153.78
153.55
142.95
136.62
135.60
134.11
129.87
128.61
126.56
120.52
118.93
116.56
81.25
52.93
51.46
49.67
42.07
40.14
39.92
39.72
39.51
39.30
39.10
38.88
28.67
28.53
25.17
24.74
22.39
22.25
21.40
19.80
OH
N
O
O
N
O
N
O
N
NH
O
192 184 176 168 160 152 144 136 128 120 112 104 96 88 80 72 64 56 48 40 32 24 16 8 0
Chemical Shift (ppm)

^{13}C NMR of **4b**

MAN6049.001.esp

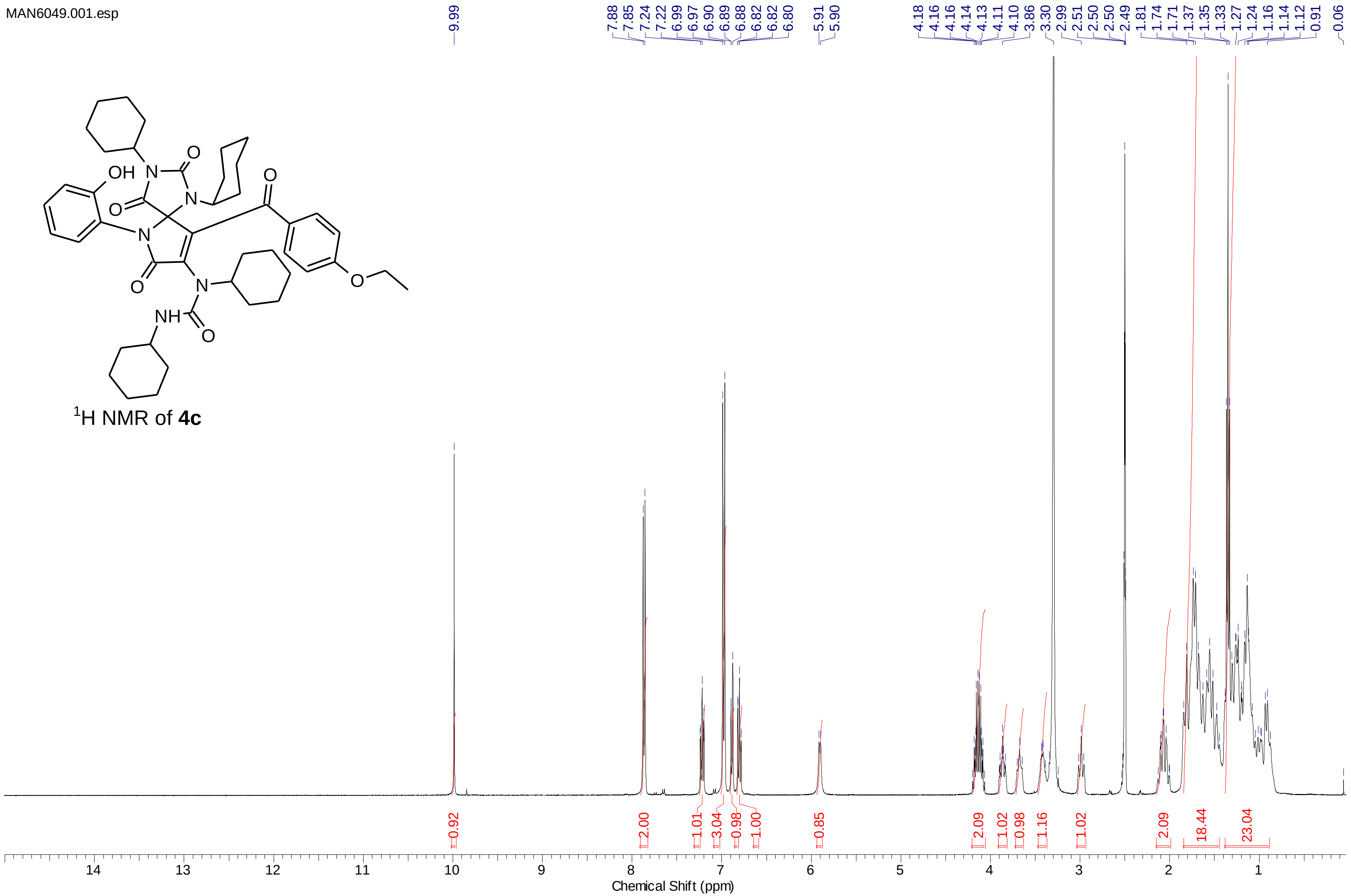

^{1}H NMR of **4c**

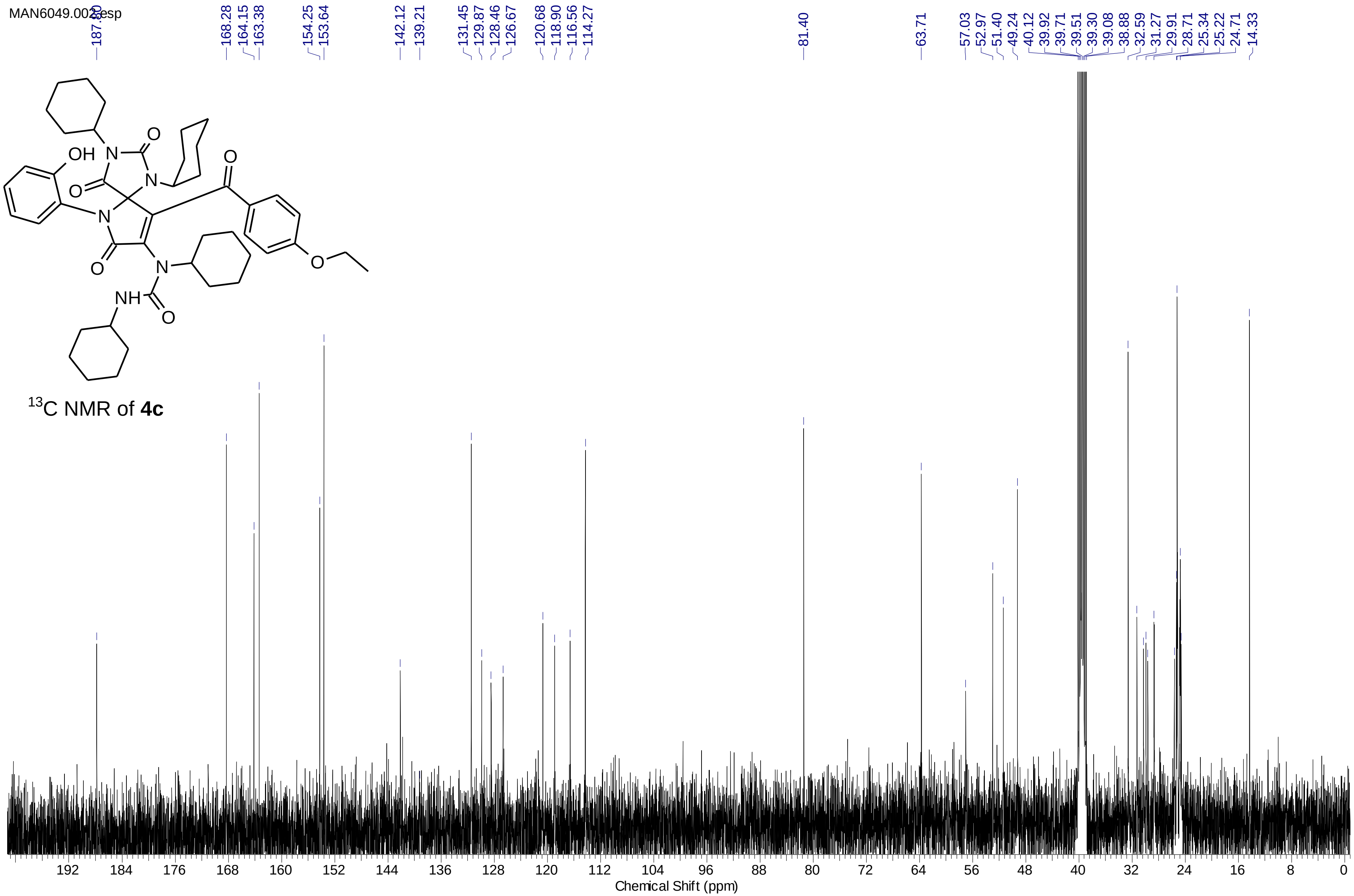
MAN6049.002.esp
187.80
168.28
164.15
163.38
154.25
153.64
142.12
139.21
131.45
129.87
128.46
126.67
120.68
118.90
116.56
114.27
81.40
63.71
57.03
52.97
51.40
49.24
40.12
39.92
39.71
39.51
39.30
39.08
38.88
32.59
31.27
29.91
28.71
25.34
25.22
24.71
14.33
OH
N
O
N
O
O
N
O
N
O
NH
O
192
184
176
168
160
152
144
136
128
120
112
104
96
88
80
72
64
56
48
40
32
24
16
8
0
Chemical Shift (ppm)

^{13}C NMR of **4c**

MAN5952.001.esp

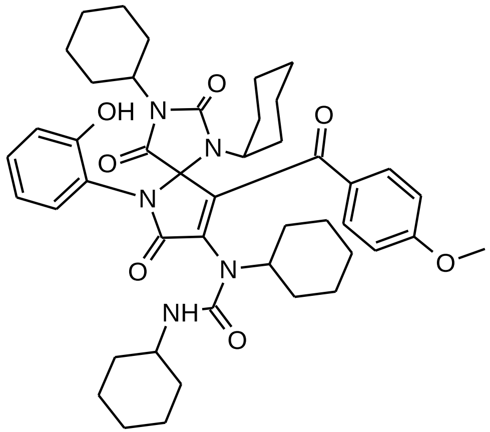

^{1}H NMR of **4d**

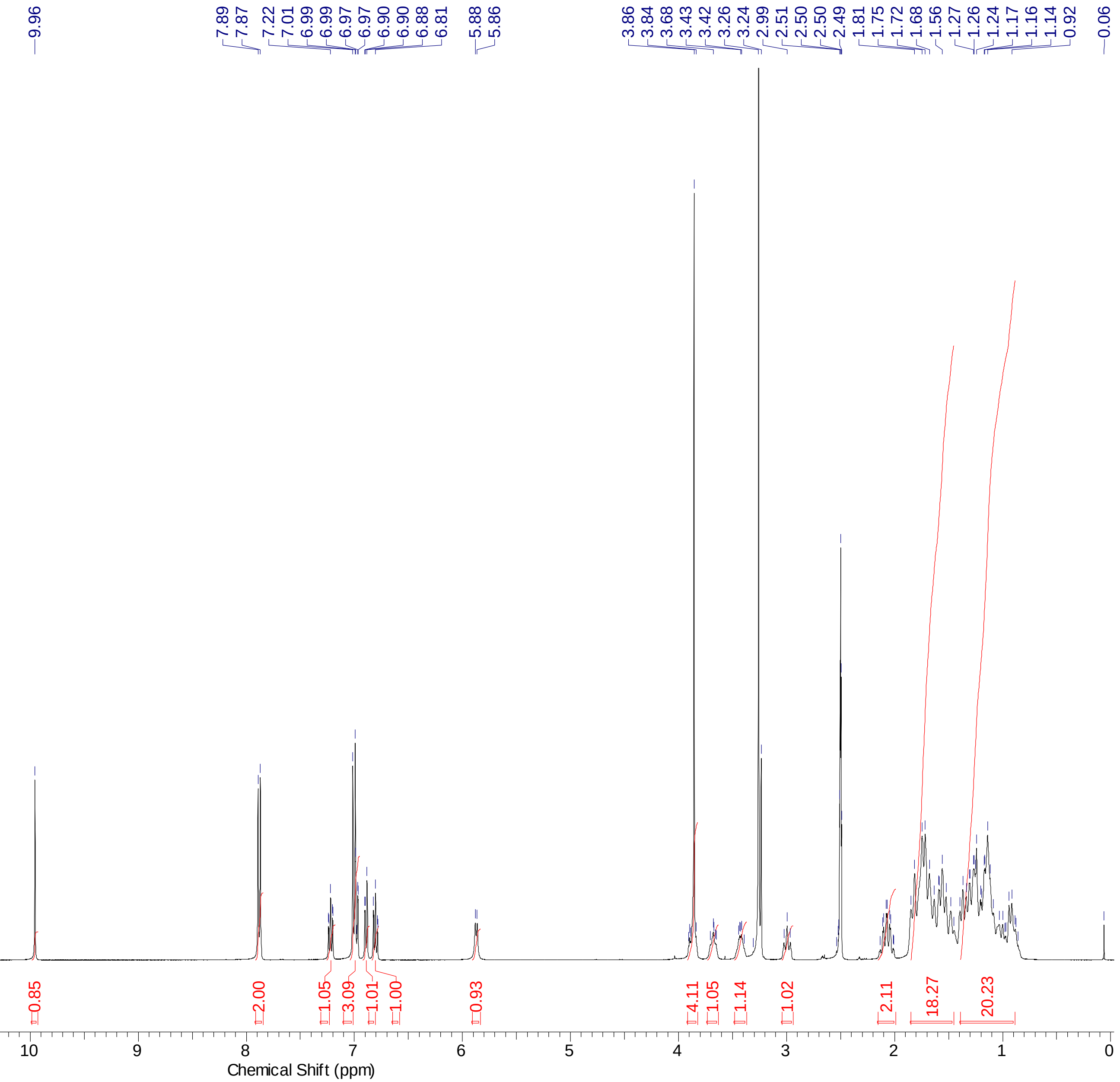

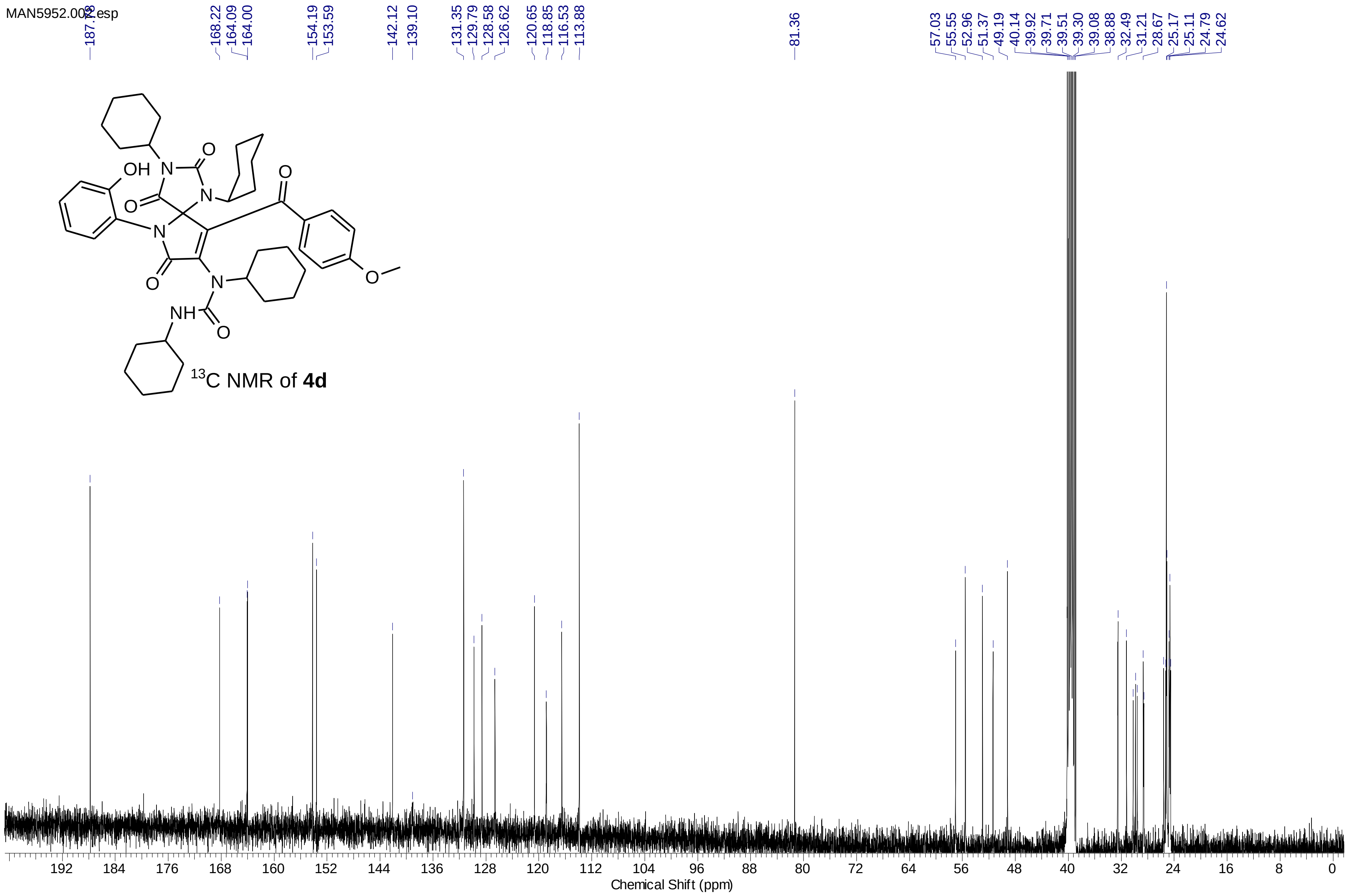

168.22
164.09
164.00
154.19
153.59
142.12
139.10
131.35
129.79
128.58
126.62
120.65
118.85
116.53
113.88
81.36
57.03
55.55
52.96
51.37
49.19
40.14
39.92
39.71
39.51
39.30
39.08
38.88
32.49
31.21
28.67
25.17
25.11
24.79
24.62
OH
O
N
NH
^{13}C NMR of **4d**
192 184 176 168 160 152 144 136 128 120 112 104 96 88 80 72 64 56 48 40 32 24 16 8 0
Chemical Shift (ppm)

MAN5956.001.esp

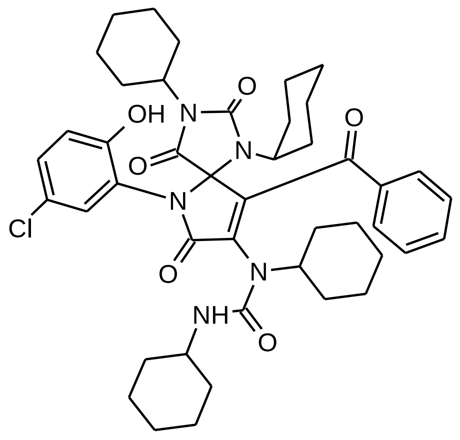

^{1}H NMR of **4e**

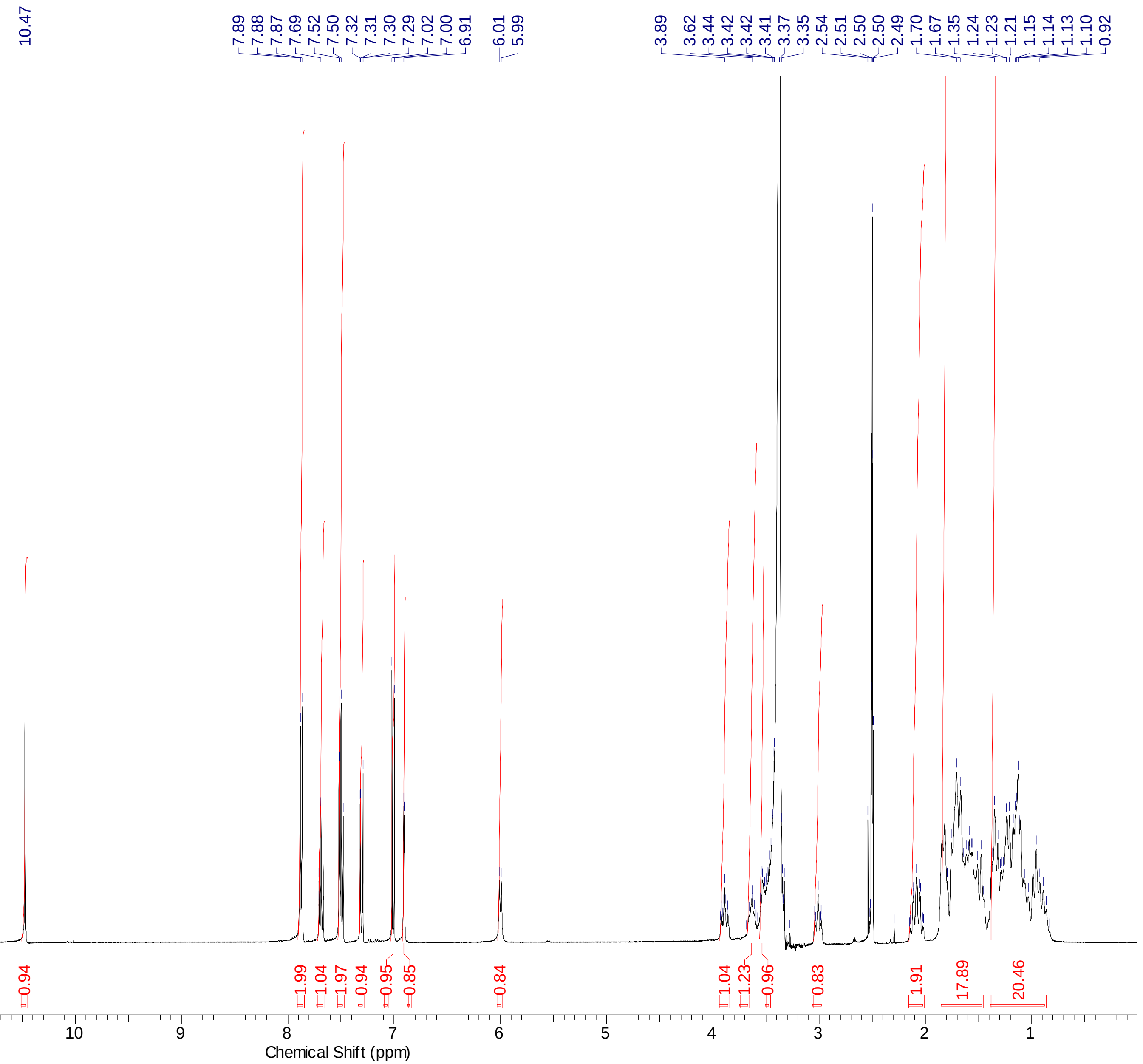

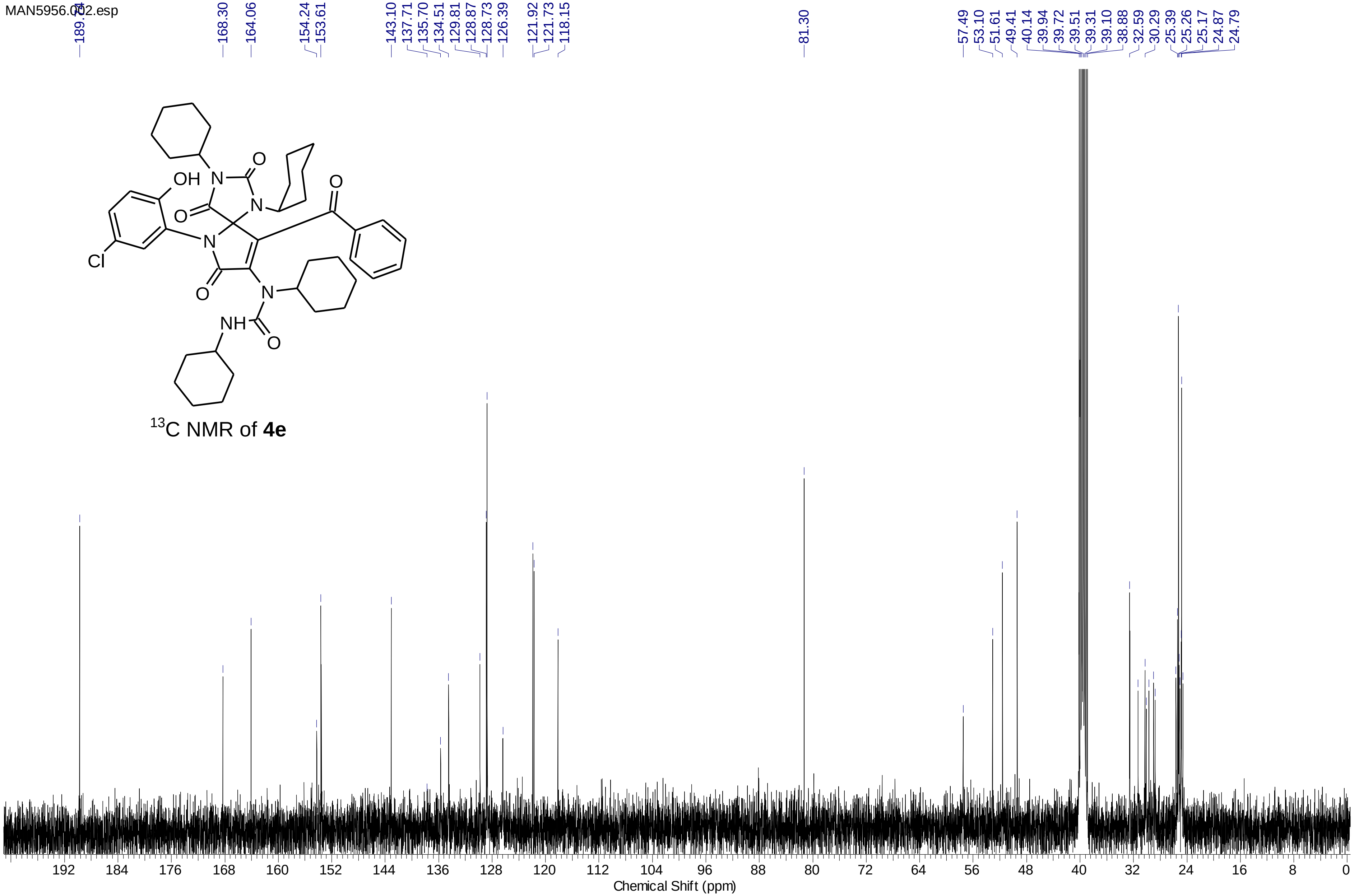
MAN5956.002.esp
189.74
168.30
164.06
154.24
153.61
143.10
137.71
135.70
134.51
129.81
128.87
128.73
126.39
121.92
121.73
118.15
81.30
57.49
53.10
51.61
49.41
40.14
39.94
39.72
39.51
39.31
39.10
38.88
32.59
30.29
25.39
25.26
25.17
24.87
24.79
OH
N
O
N
O
O
N
Cl
O
N
NH
O
192 184 176 168 160 152 144 136 128 120 112 104 96 88 80 72 64 56 48 40 32 24 16 8 0
Chemical Shift (ppm)

^{13}C NMR of **4e**

MAN5958.001.esp

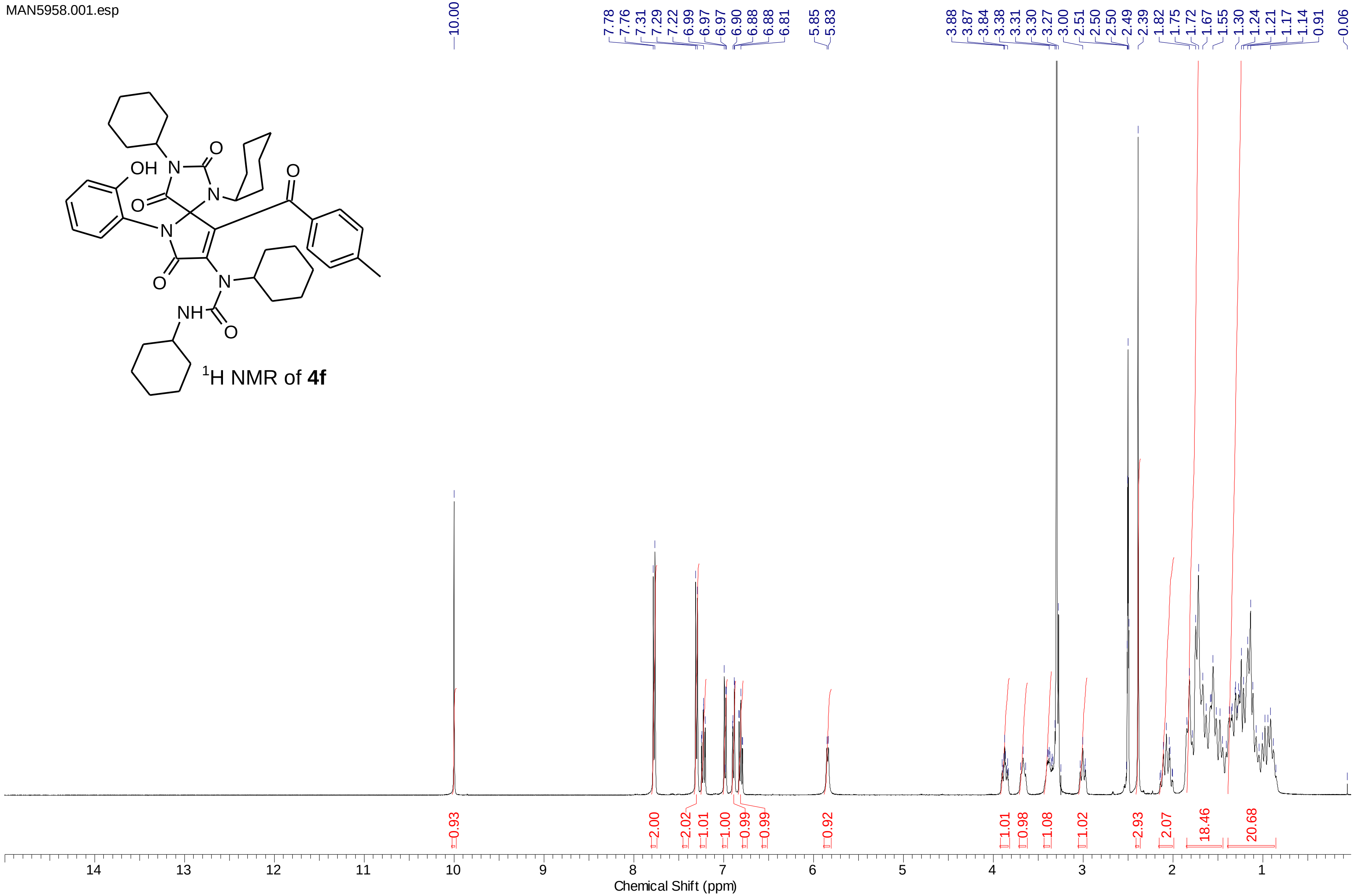

MAN5958.002.esp

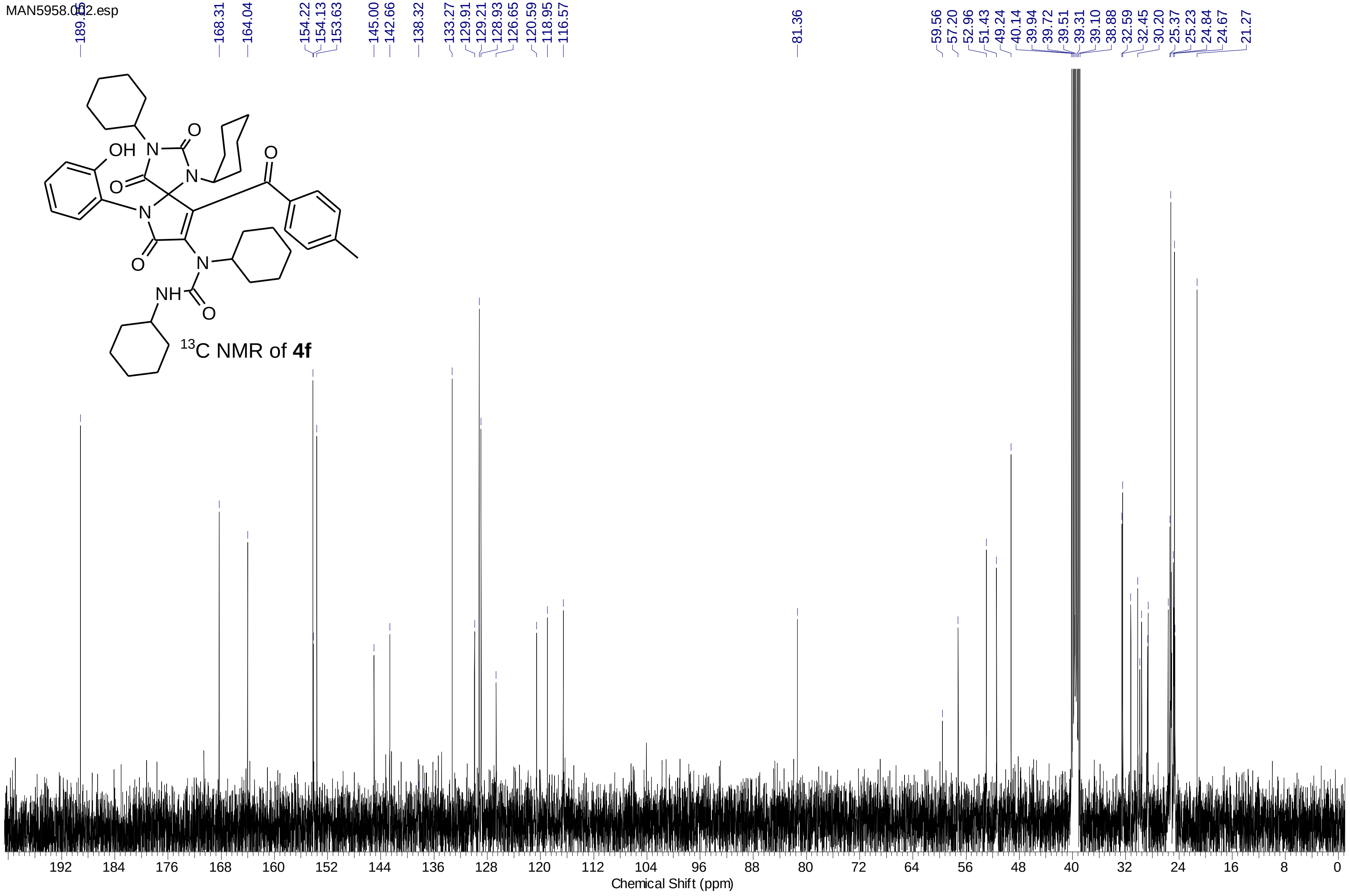

^{13}C NMR of **4f**

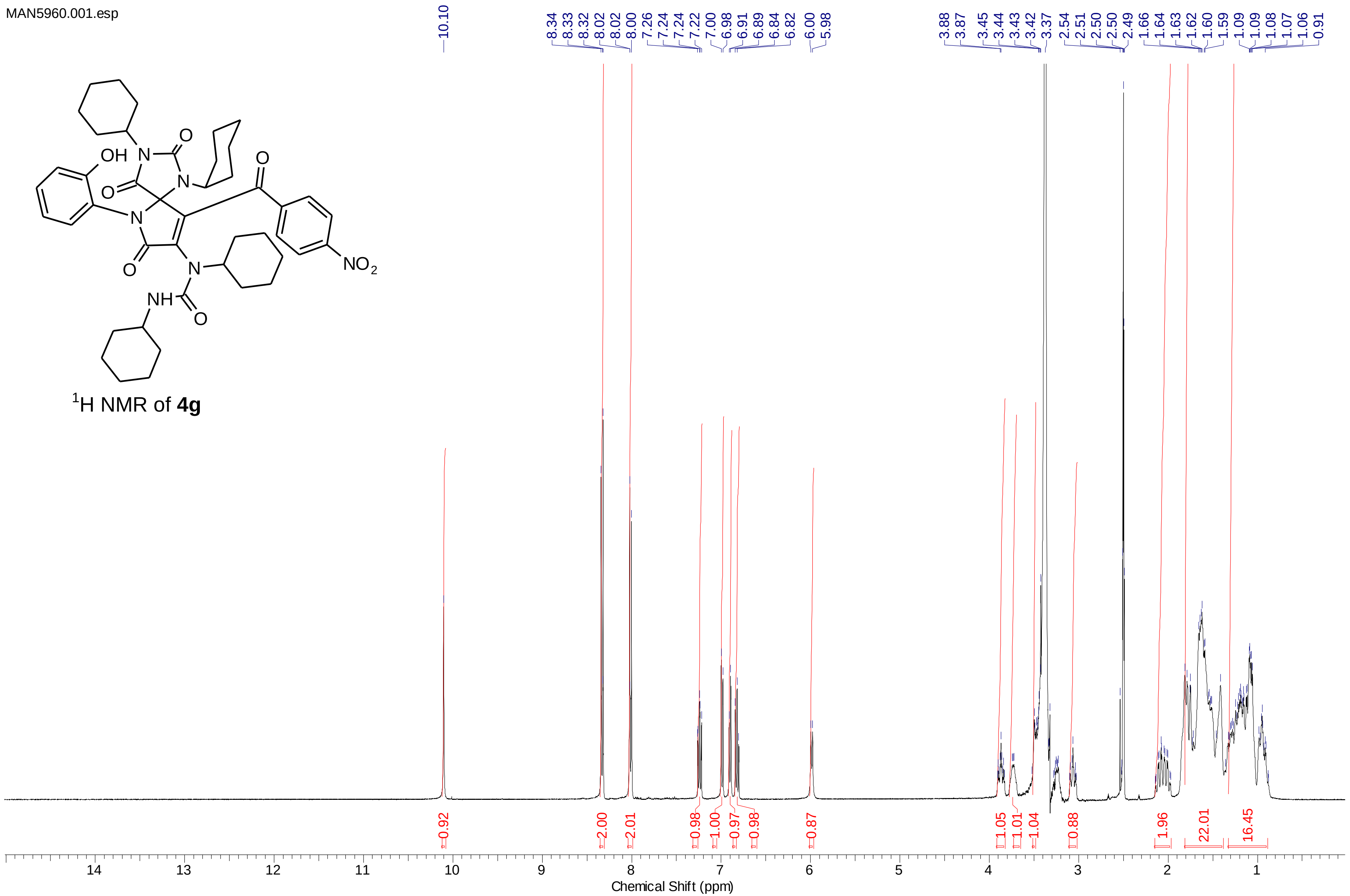

MAN5960.001.esp
10.10
8.34 8.33 8.32 8.02 8.02 8.00 7.26 7.24 7.24 7.22 7.00 6.98 6.91 6.89 6.84 6.82 6.00 5.98
3.88 3.87 3.45 3.44 3.43 3.42 3.37 2.54 2.51 2.50 2.50 2.49 1.66 1.64 1.63 1.62 1.60 1.59 1.09 1.09 1.08 1.07 1.06 0.91
0.92 2.00 2.01 0.98 1.00 0.97 0.98 0.87 1.05 1.01 1.04 0.88 1.96 22.01 16.45
14 13 12 11 10 9 8 7 6 5 4 3 2 1
Chemical Shift (ppm)

^{1}H NMR of **4g**

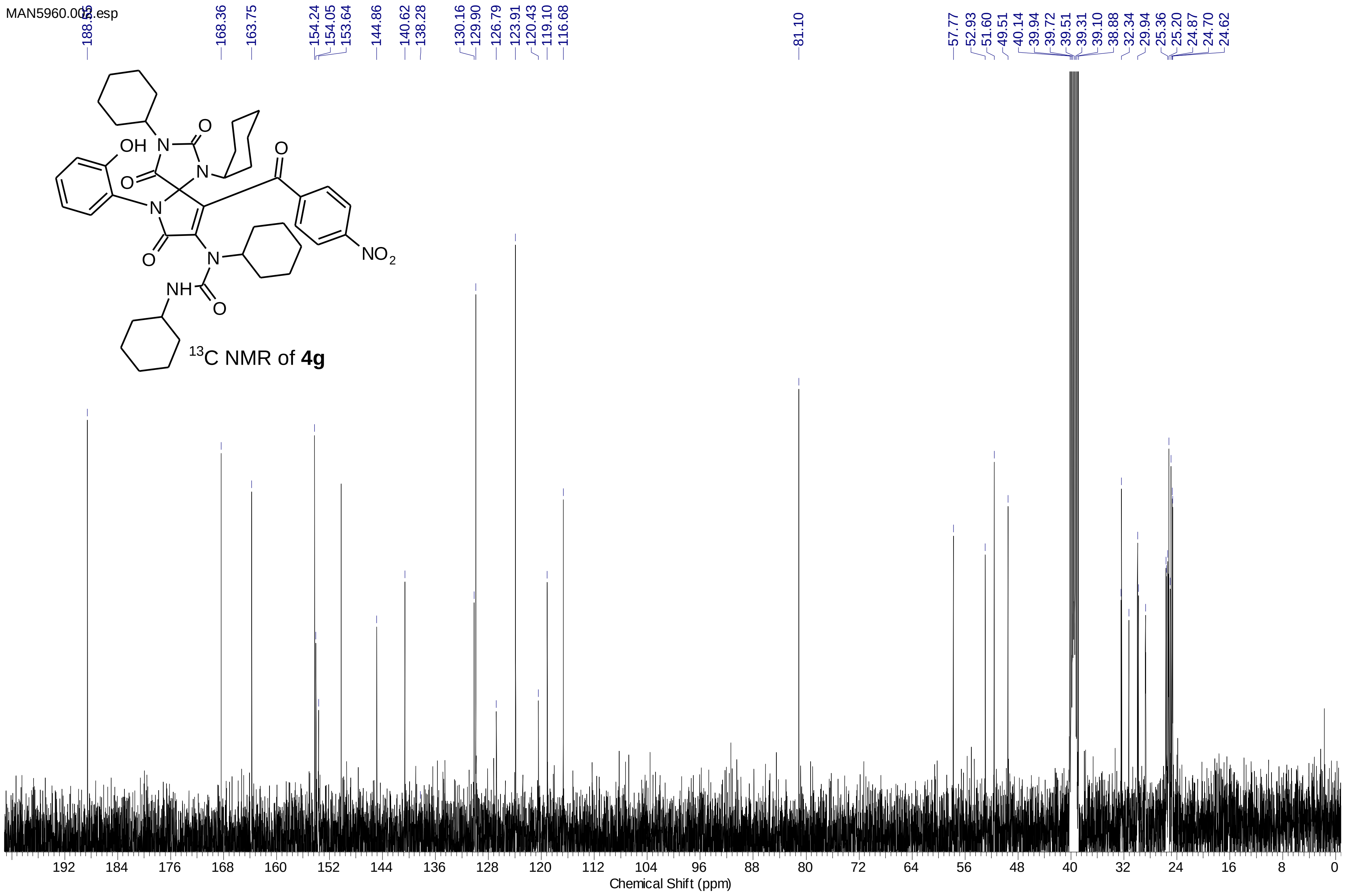

MAN5960.002.esp
168.36
163.75
154.24
154.05
153.64
144.86
140.62
138.28
130.16
129.90
126.79
123.91
120.43
119.10
116.68
81.10
57.77
52.93
51.60
49.51
40.14
39.94
39.72
39.51
39.31
39.10
38.88
32.34
29.94
25.36
25.20
24.87
24.70
24.62
OH
O
N
N
O
O
N
O
N
NO2
NH
O
13C NMR of 4g
192 184 176 168 160 152 144 136 128 120 112 104 96 88 80 72 64 56 48 40 32 24 16 8 0
Chemical Shift (ppm)

MAN6768.001.esp

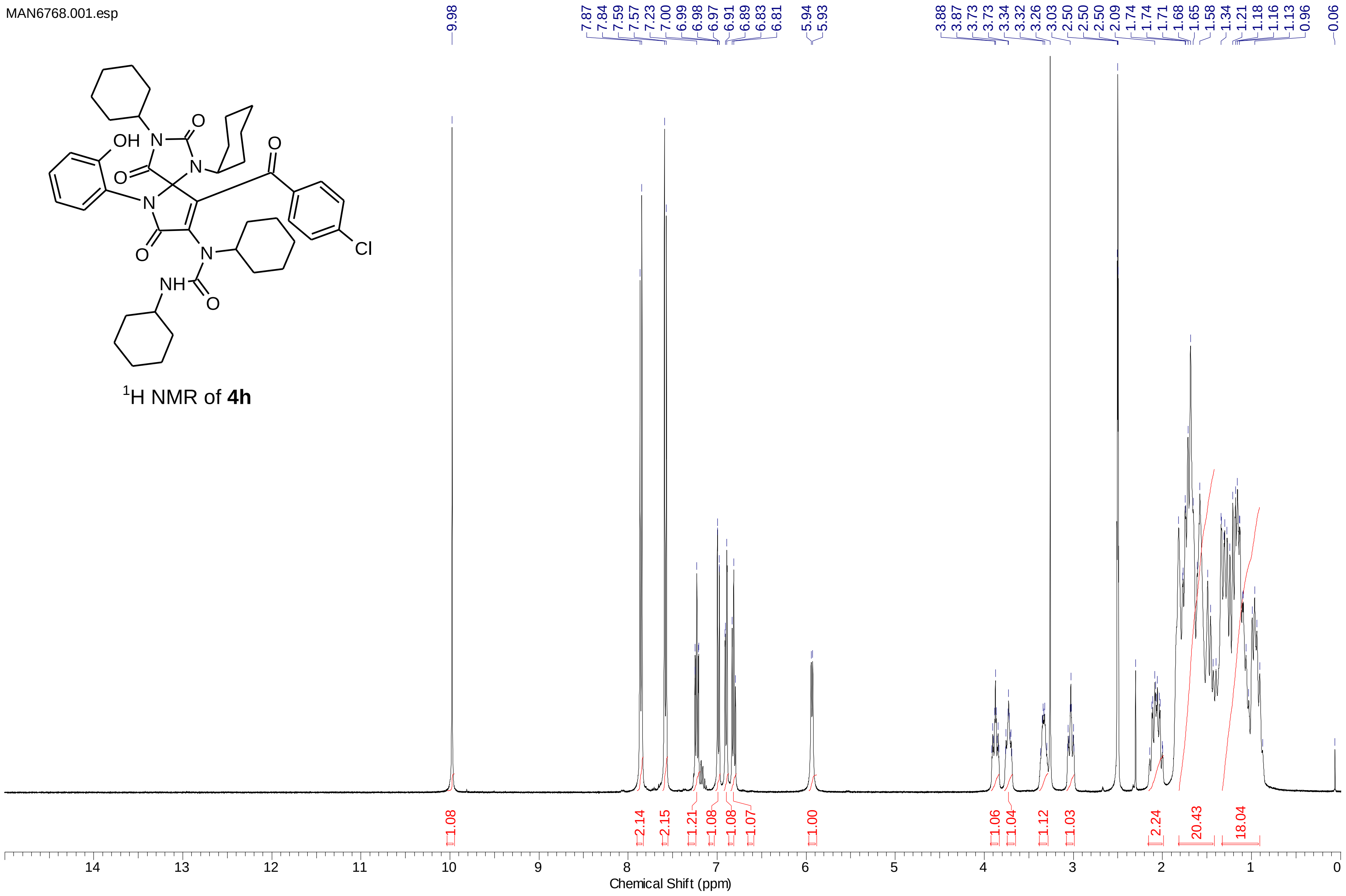

^{1}H NMR of **4h**

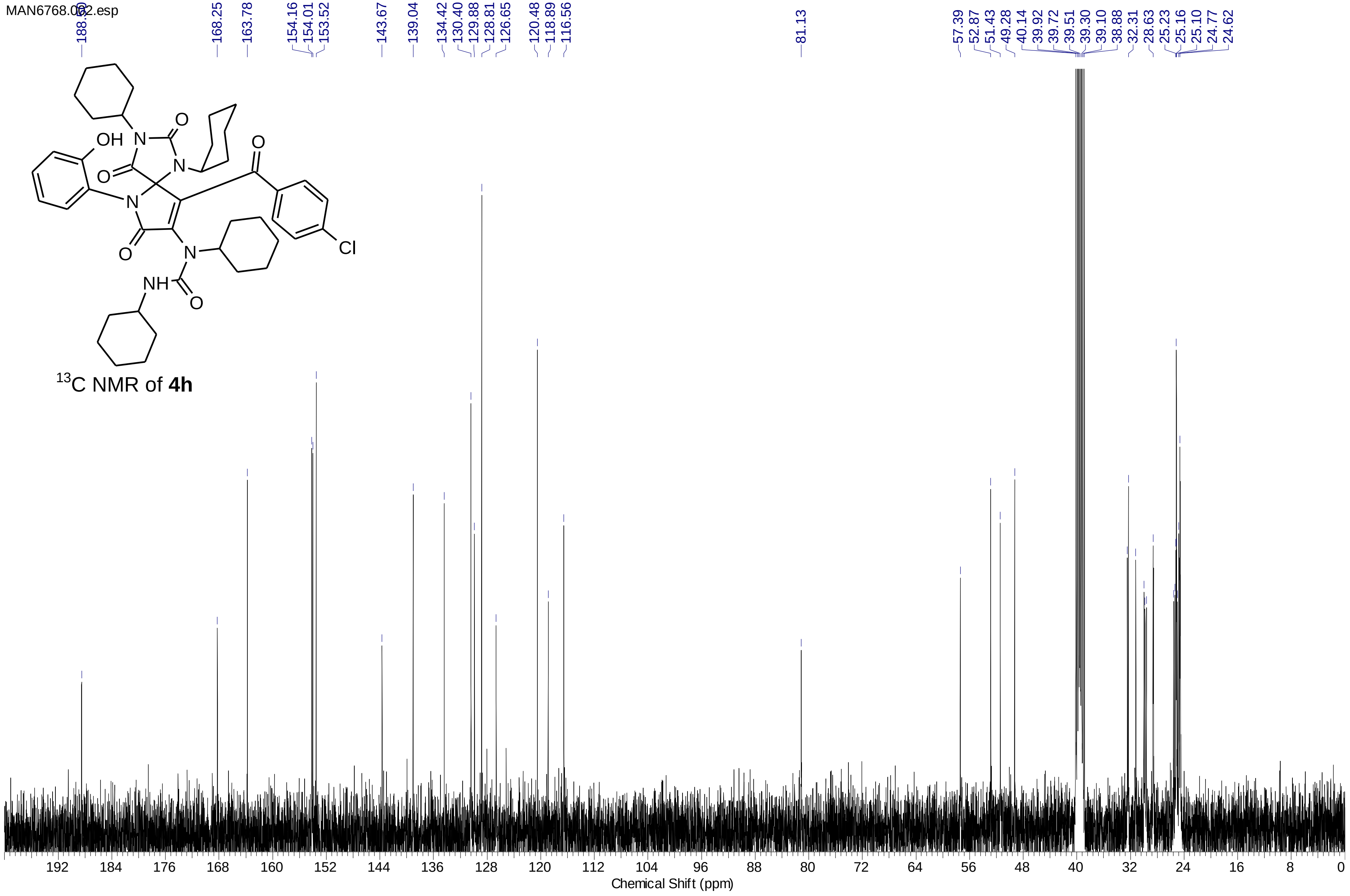
MAN6768.062.esp
188.60
168.25
163.78
154.16
154.01
153.52
143.67
139.04
134.42
130.40
129.88
128.81
126.65
120.48
118.89
116.56
81.13
57.39
52.87
51.43
49.28
40.14
39.92
39.72
39.51
39.30
39.10
38.88
32.31
28.63
25.23
25.16
25.10
24.77
24.62
OH
N
O
N
O
O
N
O
N
NH
O
Cl
192
184
176
168
160
152
144
136
128
120
112
104
96
88
80
72
64
56
48
40
32
24
16
8
0
Chemical Shift (ppm)

^{13}C NMR of **4h**

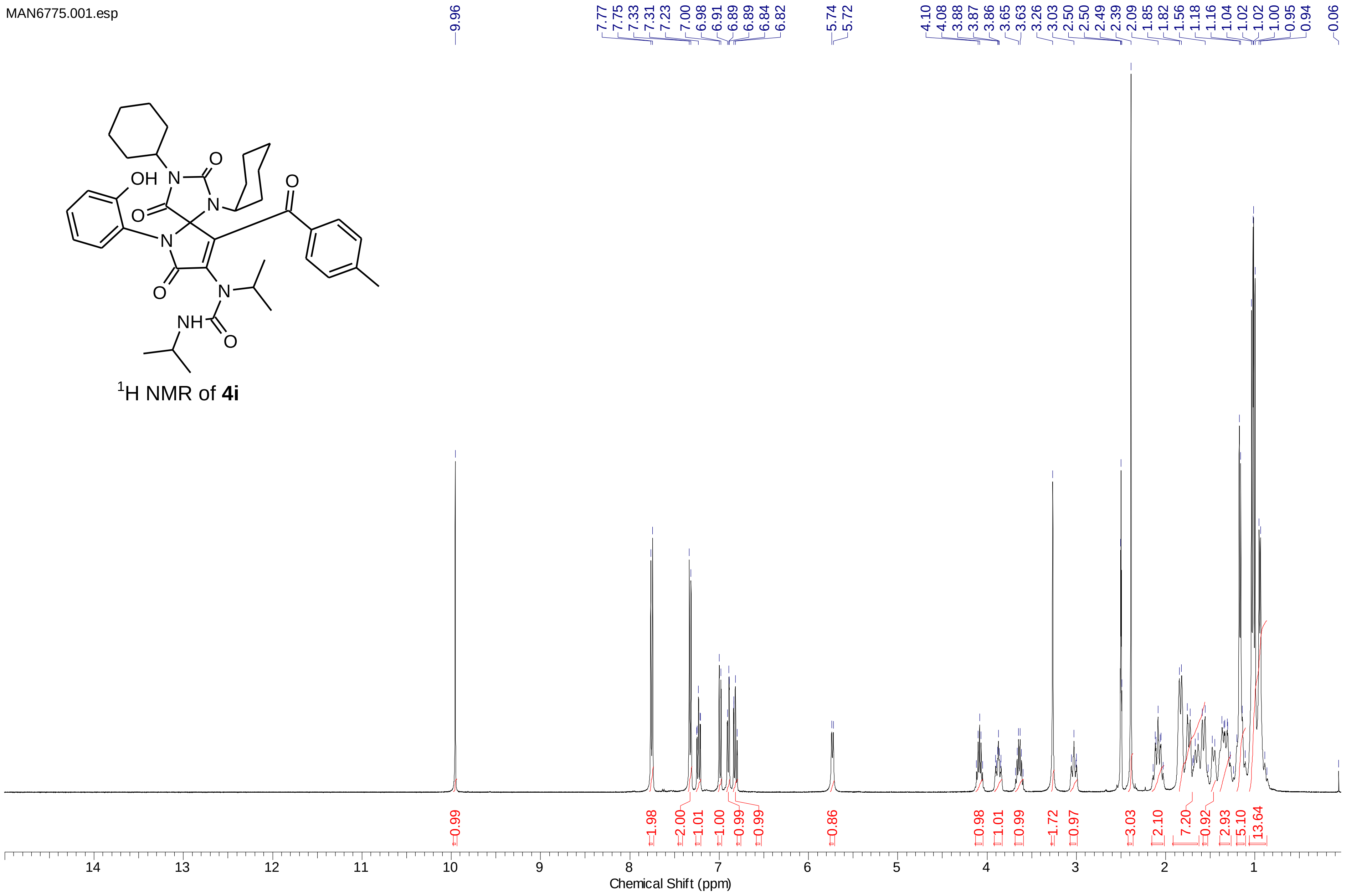
MAN6775.001.esp
9.96
7.77 7.75 7.33 7.31 7.23 7.00 6.98 6.91 6.89 6.89 6.84 6.82
5.74 5.72
4.10 4.08 3.88 3.87 3.86 3.65 3.63 3.26 3.03 2.50 2.50 2.49 2.39 2.09 1.85 1.82 1.56 1.18 1.16 1.04 1.02 1.02 1.00 0.95 0.94
0.06
OH
N
O
NH
0.99 1.98 2.00 1.01 1.00 0.99 0.99 0.86 0.98 1.01 0.99 1.72 0.97 3.03 2.10 7.20 0.92 2.93 5.10 13.64
14 13 12 11 10 9 8 7 6 5 4 3 2 1
Chemical Shift (ppm)

^{1}H NMR of **4i**

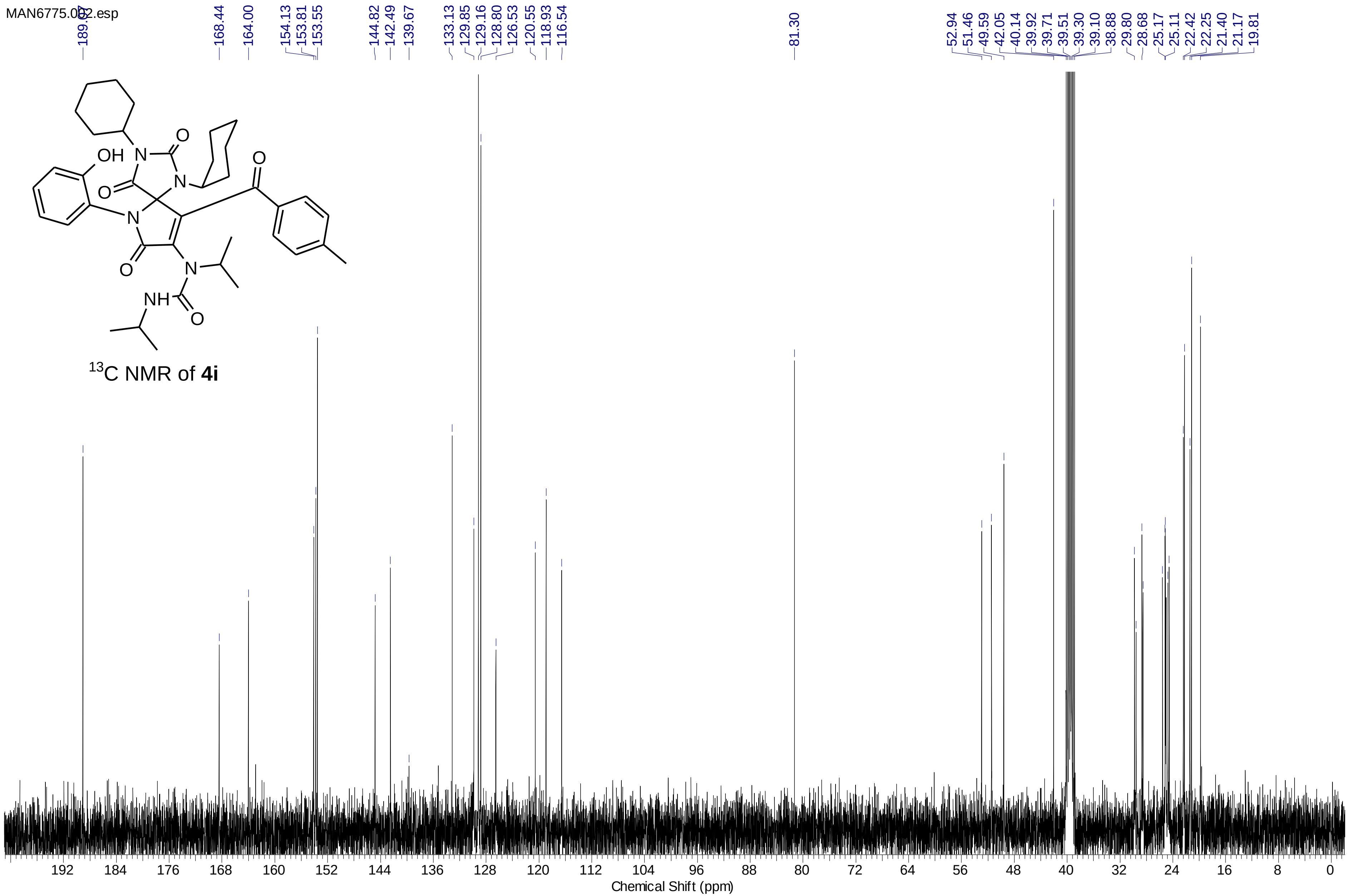
MAN6775.002.esp
189.07
168.44
164.00
154.13
153.81
153.55
144.82
142.49
139.67
133.13
129.85
129.16
128.80
126.53
120.55
118.93
116.54
81.30
52.94
51.46
49.59
42.05
40.14
39.92
39.71
39.51
39.30
39.10
38.88
29.80
28.68
25.17
25.11
22.42
22.25
21.40
21.17
19.81
OH
N
O
N
O
O
N
O
N
NH
O
192 184 176 168 160 152 144 136 128 120 112 104 96 88 80 72 64 56 48 40 32 24 16 8 0
Chemical Shift (ppm)

^13^C NMR of **4i**

MAN6706.001.esp

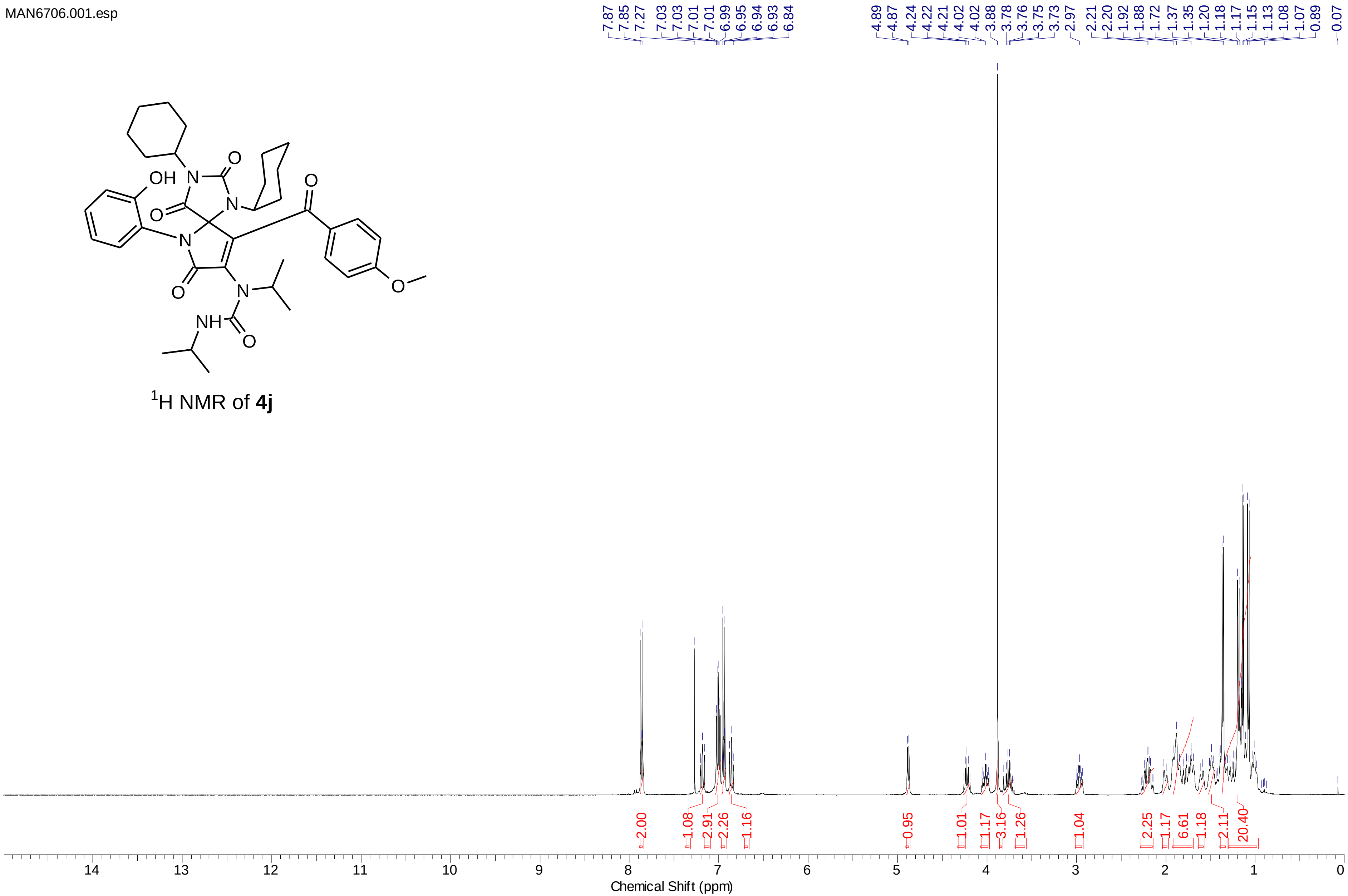

^{1}H NMR of **4j**

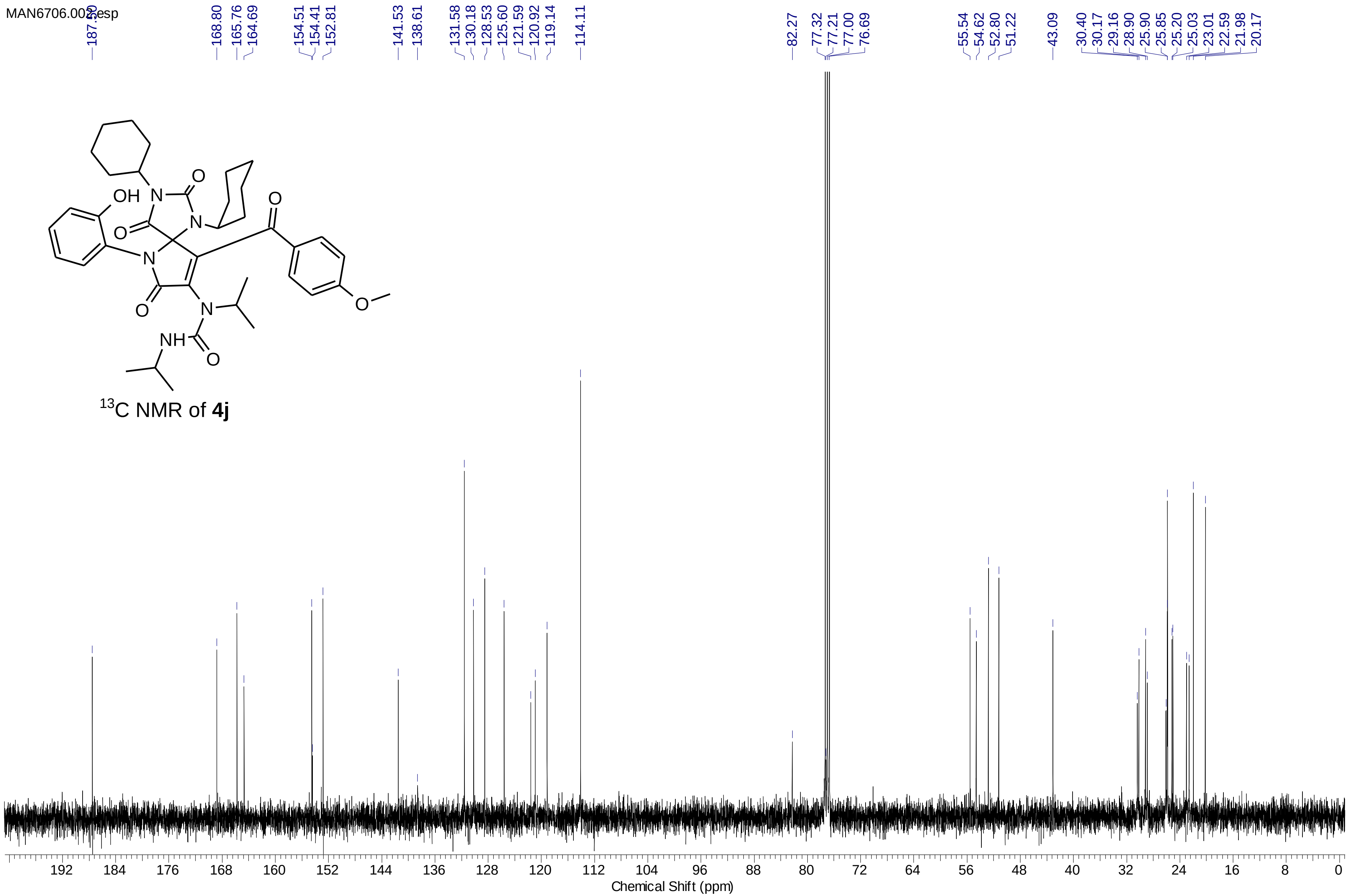
MAN6706.002.esp
187.50
168.80
165.76
164.69
154.51
154.41
152.81
141.53
138.61
131.58
130.18
128.53
125.60
121.59
120.92
119.14
114.11
82.27
77.32
77.21
77.00
76.69
55.54
54.62
52.80
51.22
43.09
30.40
30.17
29.16
28.90
25.90
25.85
25.20
25.03
23.01
22.59
21.98
20.17
OH
N
O
O
N
O
N
O
N
NH
O
O
192 184 176 168 160 152 144 136 128 120 112 104 96 88 80 72 64 56 48 40 32 24 16 8 0
Chemical Shift (ppm)

^{13}C NMR of **4j**

MAN6707.001.esp

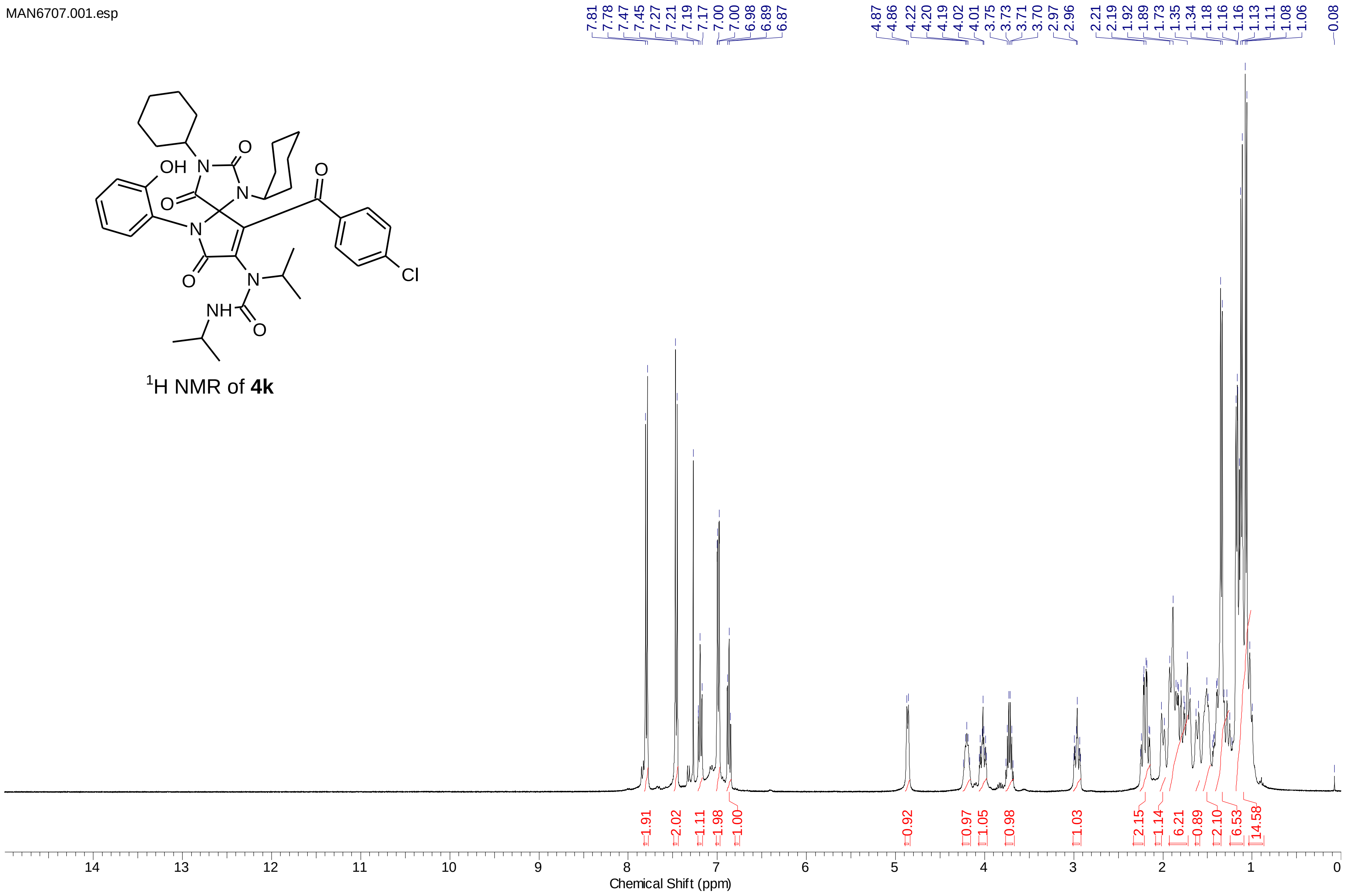

^{1}H NMR of **4k**

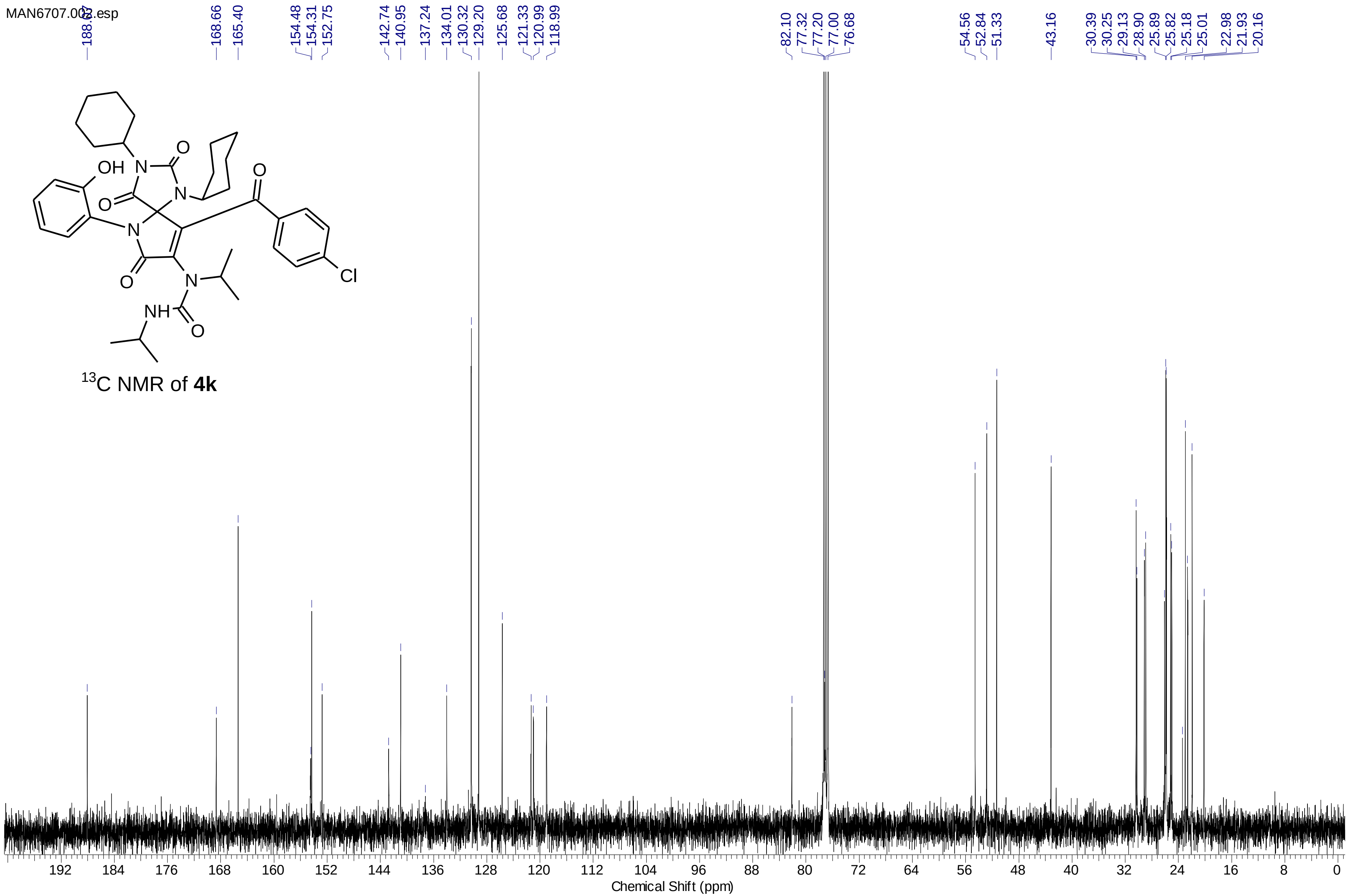
MAN6707.002.esp
188.07
168.66
165.40
154.48
154.31
152.75
142.74
140.95
137.24
134.01
130.32
129.20
125.68
121.33
120.99
118.99
82.10
77.32
77.20
77.00
76.68
54.56
52.84
51.33
43.16
30.39
30.25
29.13
28.90
25.89
25.82
25.18
25.01
22.98
21.93
20.16
OH
N
O
N
O
O
N
O
N
NH
O
Cl
192
184
176
168
160
152
144
136
128
120
112
104
96
88
80
72
64
56
48
40
32
24
16
8
0
Chemical Shift (ppm)

^{13}C NMR of **4k**

MAN7302.001.esp

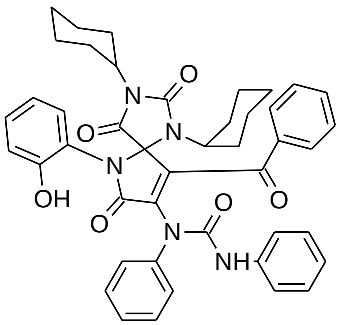

^{1}H NMR of **4l**

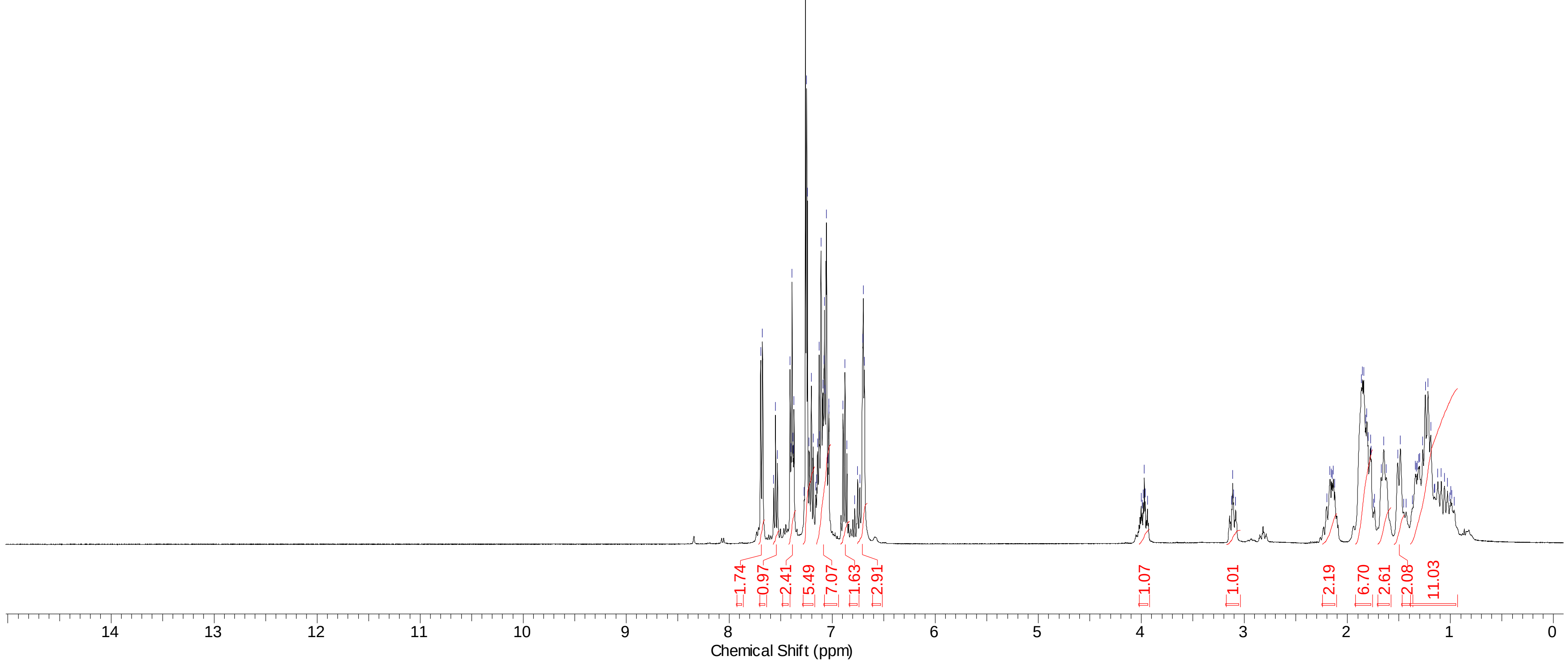

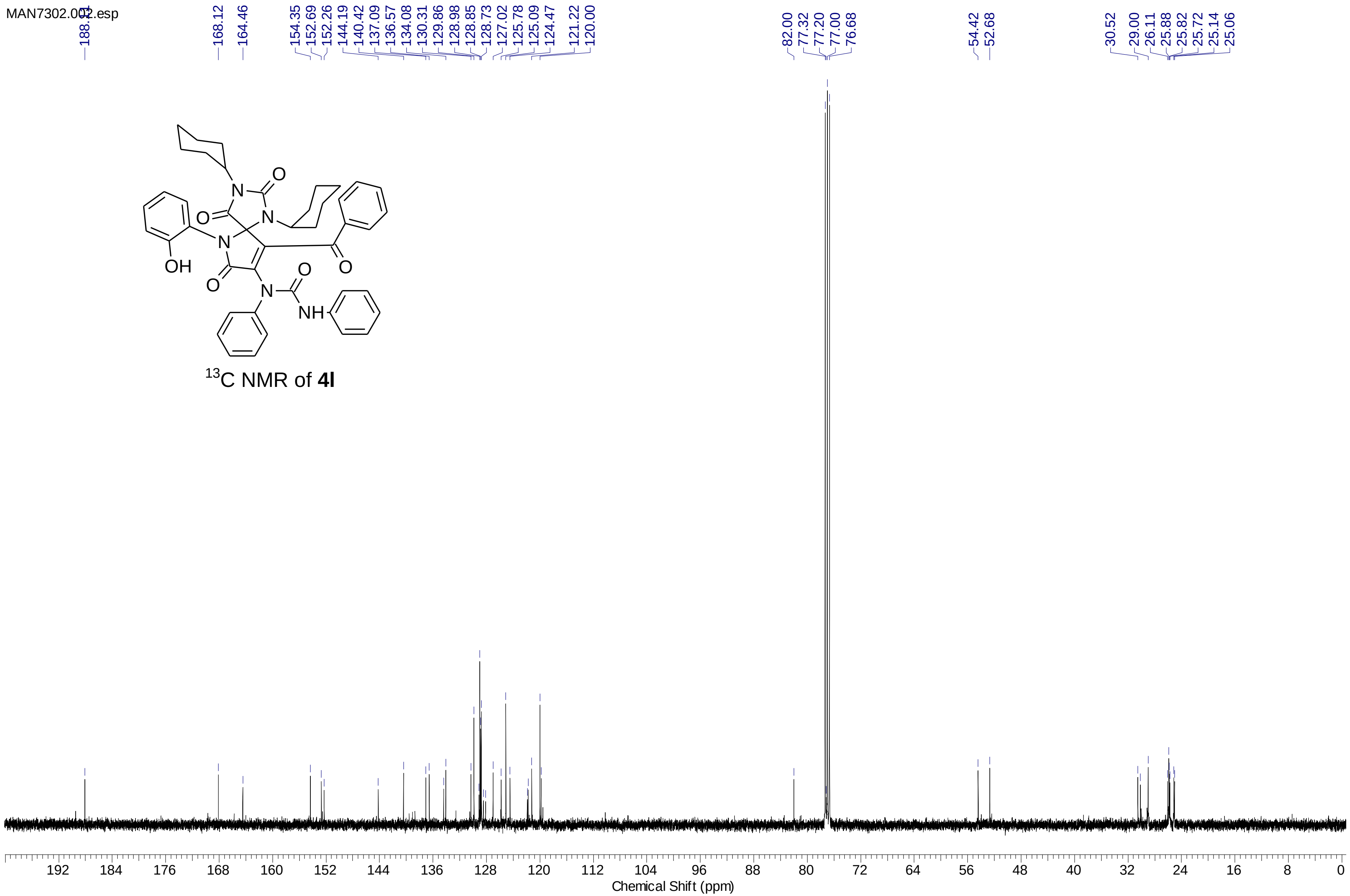

MAN7302.002.esp
188.11
168.12
164.46
154.35
152.69
152.26
144.19
140.42
137.09
136.57
134.08
130.31
129.86
128.98
128.85
128.73
127.02
125.78
125.09
124.47
121.22
120.00
82.00
77.32
77.20
77.00
76.68
54.42
52.68
30.52
29.00
26.11
25.88
25.82
25.72
25.14
25.06
O
N
O
N
O
N
OH
O
N
O
O
NH
192
184
176
168
160
152
144
136
128
120
112
104
96
88
80
72
64
56
48
40
32
24
16
8
0
Chemical Shift (ppm)

^{13}C NMR of **4l**

MAN7303.001.esp

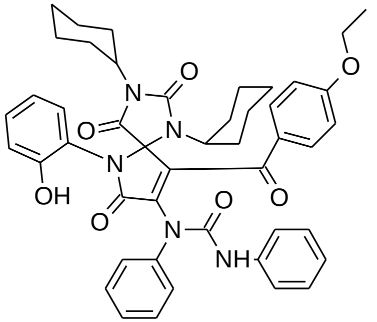

^{1}H NMR of **4m**

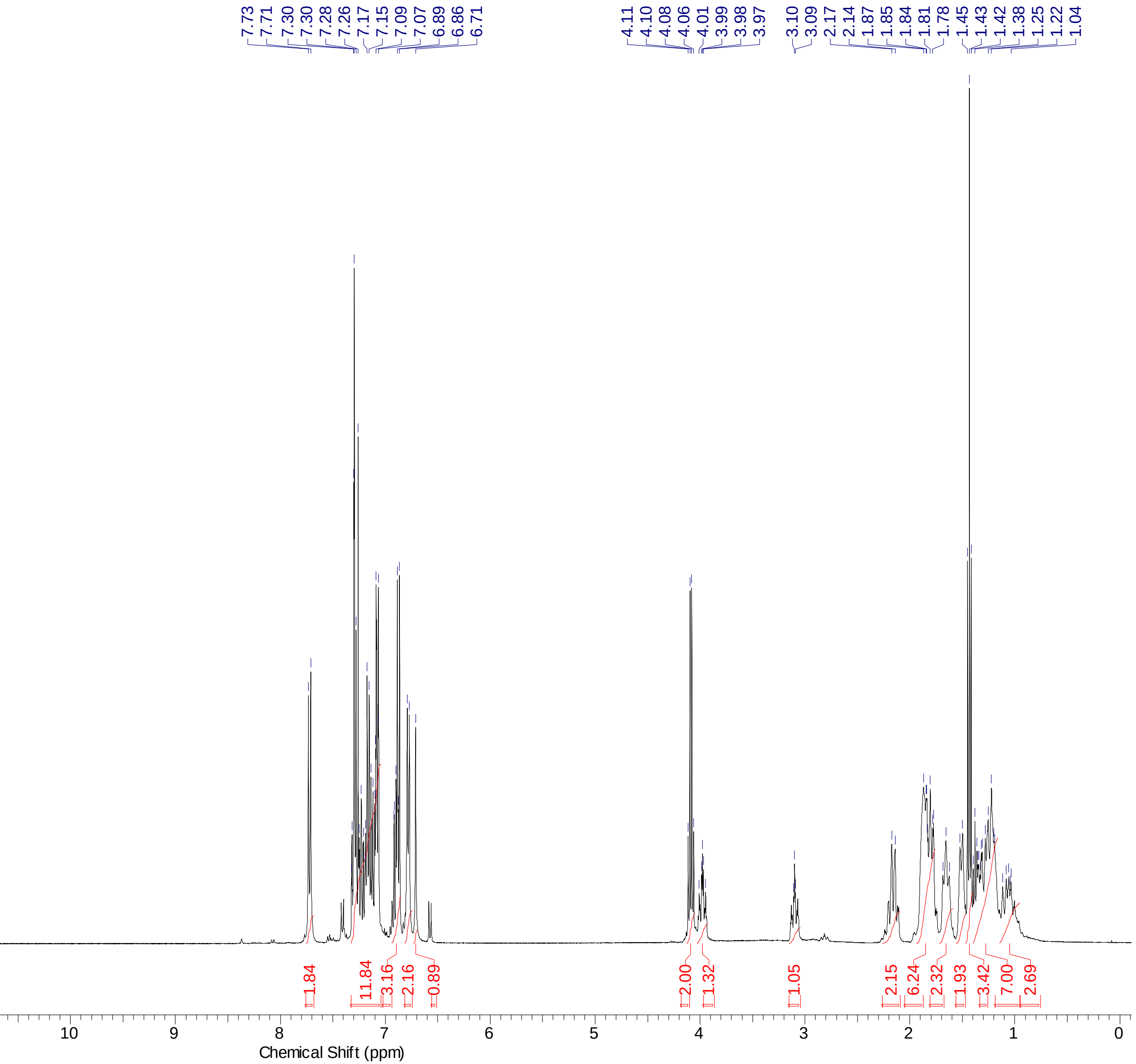

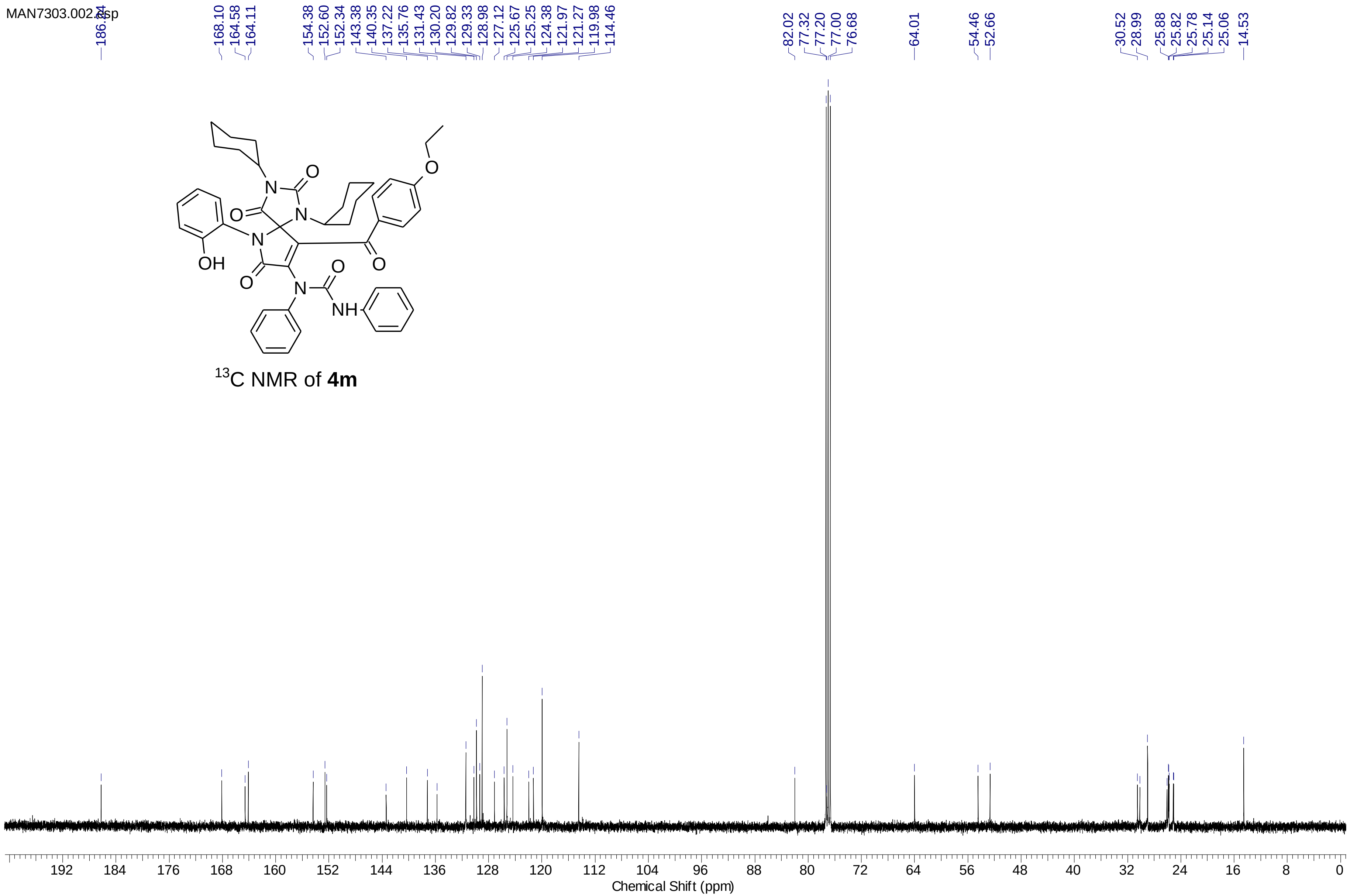
MAN7303.002.esp
186.24
168.10
164.58
164.11
154.38
152.60
152.34
143.38
140.35
137.22
135.76
131.43
130.20
129.82
129.33
128.98
127.12
125.67
125.25
124.38
121.97
121.27
119.98
114.46
82.02
77.32
77.20
77.00
76.68
64.01
54.46
52.66
30.52
28.99
25.88
25.82
25.78
25.14
25.06
14.53
N
O
OH
NH
192 184 176 168 160 152 144 136 128 120 112 104 96 88 80 72 64 56 48 40 32 24 16 8 0
Chemical Shift (ppm)

^{13}C NMR of **4m**

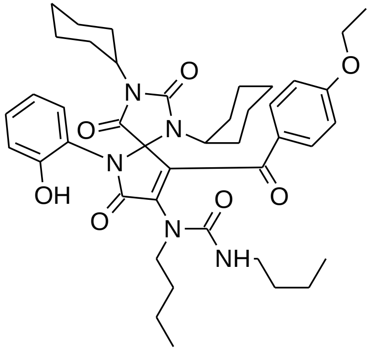

^{1}H NMR of **4n**

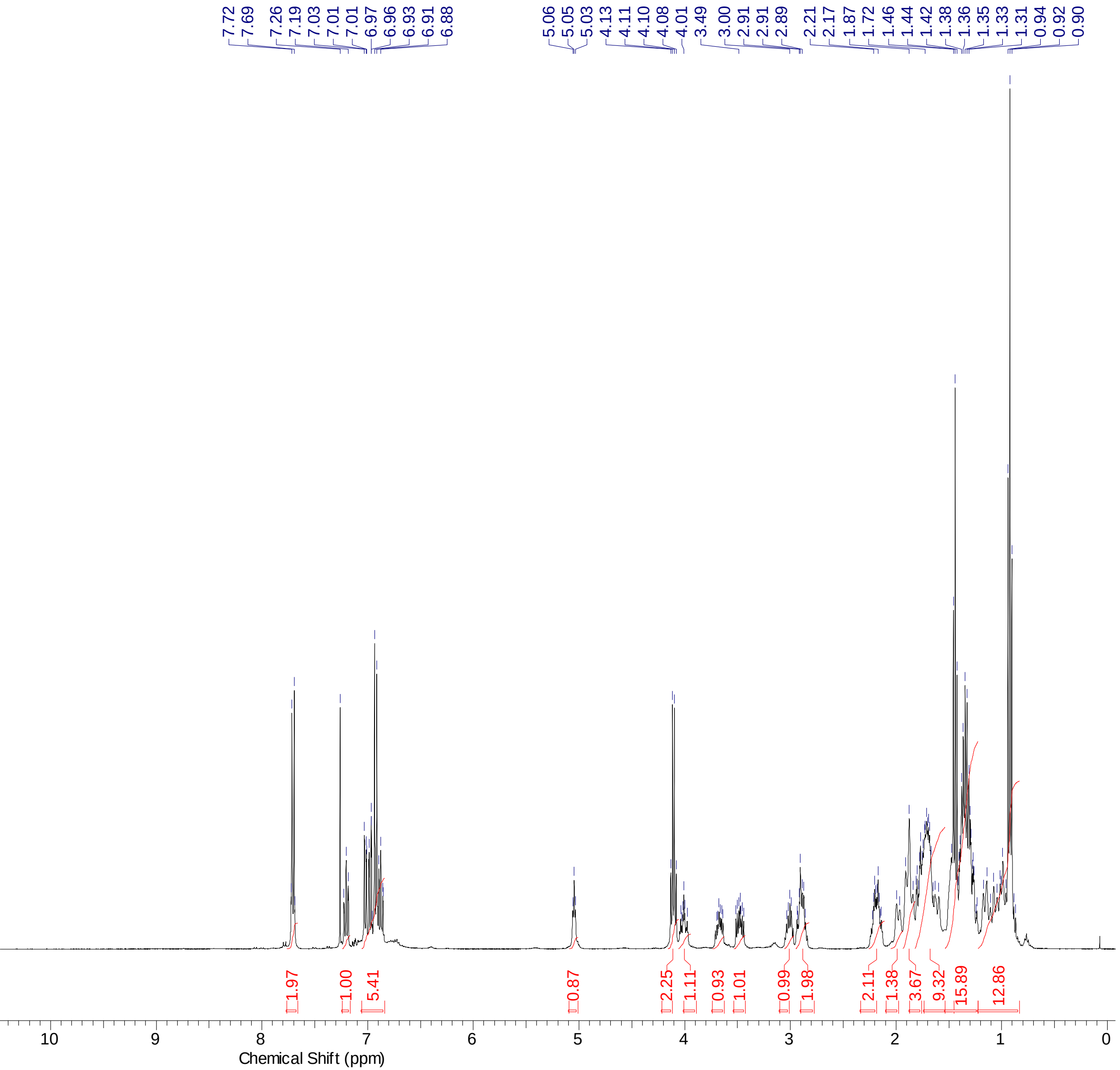

MAN7315.001.esp
7.72
7.69
7.26
7.19
7.03
7.01
7.01
6.97
6.96
6.93
6.91
6.88
5.06
5.05
5.03
4.13
4.11
4.10
4.08
4.01
3.49
3.00
2.91
2.91
2.89
2.21
2.17
1.87
1.72
1.46
1.44
1.42
1.38
1.36
1.35
1.33
1.31
0.94
0.92
0.90
1.97
1.00
5.41
0.87
2.25
1.11
0.93
1.01
0.99
1.98
2.11
1.38
3.67
9.32
15.89
12.86
14
13
12
11
10
9
8
7
6
5
4
3
2
1
0
Chemical Shift (ppm)

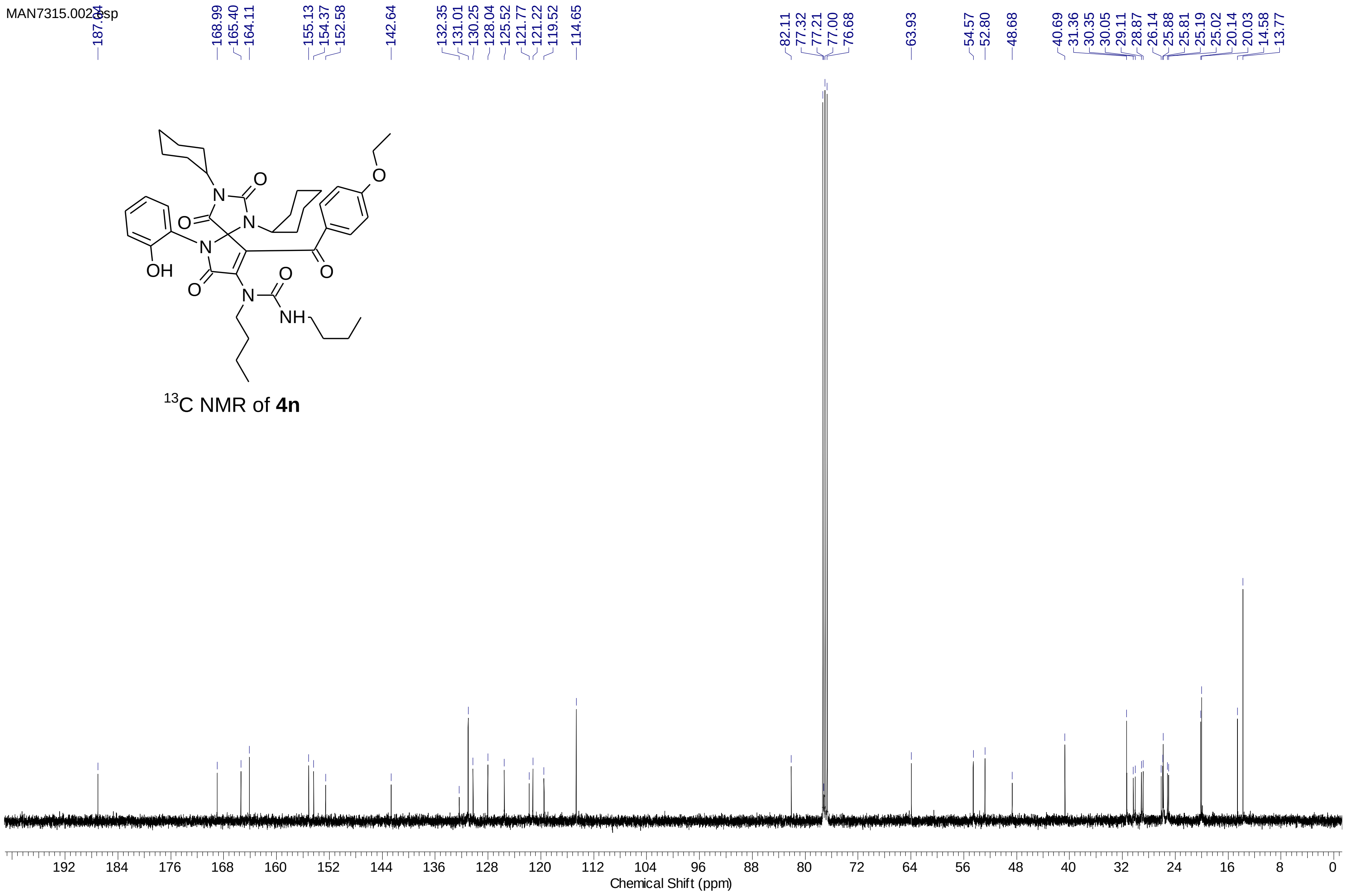
MAN7315.002.esp
187.04
168.99
165.40
164.11
155.13
154.37
152.58
142.64
132.35
131.01
130.25
128.04
125.52
121.77
121.22
119.52
114.65
82.11
77.32
77.21
77.00
76.68
63.93
54.57
52.80
48.68
40.69
31.36
30.35
30.05
29.11
28.87
26.14
25.88
25.81
25.19
25.02
20.14
20.03
14.58
13.77
Chemical Shift (ppm)

^{13}C NMR of **4n**

General procedure to compounds 3a-n

The corresponding compound **4** (0.5 mmol) was put into an oven-dried tube, pressed slightly, and then, it was heated at 160–290 °C (the temperature for each compound is given in the Table 2 of the main manuscript; caution: R′NCO evolves during the reaction). The reaction mixture was cooled to room temperature and scrubbed with hexane (about 10 mL) to give the appropriate compound **3**.

9-Benzoyl-1,3-dicyclohexyl-8-(cyclohexylamino)-6-(2-hydroxyphenyl)-1,3,6-triazaspiro[4.4]non-8-ene-2,4,7-trione (3a)

Yield: 300 mg (96%); white solid; mp 215–217 °C.

^{1}H NMR (400 MHz, $CDCl_3$): δ = 7.69 (m, 2 H), 7.59 (m, 1 H), 7.51 (m, 2 H), 7.24 (m, 1 H), 7.08 (m, 1 H), 7.03 (m, 1 H), 6.91 (m, 1 H), 6.45 (br. s, 1 H), 5.60 (d, 1 H, *J* = 8.0 Hz), 3.99 (m, 1 H), 2.84 (m, 1 H), 2.22-2.10 (m, 2 H), 1.97-1.82 (m, 6 H), 1.68-1.53 (m, 7 H), 1.45-0.75 (m, 16 H) ppm.

^{13}C NMR (100 MHz, $CDCl_3$): δ = 189.7, 170.3, 165.7, 154.7, 152.4, 139.9, 132.7, 130.4, 129.0 (2 C), 128.1, 126.1, 122.1, 121.5 (2 C), 120.0, 107.1, 83.1, 53.8, 52.3, 33.9, 32.9, 32.4, 30.5, 30.0, 29.1, 29.0, 26.4, 26.0, 25.9, 25.9, 25.2, 25.2, 25.1, 24.5, 24.2 ppm.

IR (mineral oil): 3363, 3276, 1767, 1724, 1700 cm^{-1}.

Anal. Calcd (%) for $C_{37}H_{44}N_4O_5$: C 71.13; H 7.10; N 8.97. Found: C 71.20; H 7.17; N 8.93.

9-Benzoyl-1,3-dicyclohexyl-6-(2-hydroxyphenyl)-8-(isopropylamino)-1,3,6-triazaspiro[4.4]non-8-ene-2,4,7-trione (3b)

Yield: 263 mg (90%); white solid; mp 147–149 °C.

^{1}H NMR (400 MHz, $CDCl_3$): δ = 7.70 (m, 2 H), 7.58 (m, 1 H), 7.49 (m, 2 H), 7.23 (m, 1 H), 7.05 (m, 2 H), 6.90 (m, 1 H), 6.49 (m, 1 H), 5.49 (d, 1 H, *J* = 8.0 Hz), 3.98 (m, 1 H), 3.29 (m, 1 H), 2.84 (m, 1 H), 2.22-2.09 (m, 2 H), 1.97-1.76 (m, 5 H), 1.74-1.66 (m, 3 H), 1.58-1.51 (m, 2 H), 1.39-1.04 (m, 8 H), 0.99-0.94 (m, 6 H) ppm.

^{13}C NMR (100 MHz, $CDCl_3$): δ = 189.6, 170.2, 165.6, 154.6, 152.4, 139.6, 132.8, 130.4, 129.0 (2 C), 128.0 (2 C), 126.0, 122.0, 121.5, 119.9, 107.7, 83.0, 53.8, 52.3, 46.9, 30.5, 30.0, 29.0, 29.0, 26.4, 26.0, 25.9, 25.8, 25.2, 25.2, 22.7, 22.4 ppm.

IR (mineral oil): 3354, 3262, 3170, 1726 cm^{-1}.

Anal. Calcd (%) for $C_{34}H_{40}N_4O_5$: C 69.84; H 6.90; N 9.58. Found: C 69.93; H 6.84; N 9.61.

1,3-Dicyclohexyl-8-(cyclohexylamino)-9-(4-ethoxybenzoyl)-6-(2-hydroxyphenyl)-1,3,6-triazaspiro[4.4]non-8-ene-2,4,7-trione (3c)

Yield: 311 mg (93%); white solid; mp 152–154 °C.

^{1}H NMR (400 MHz, $CDCl_3$): δ = 7.70 (m, 2 H), 7.23 (m, 1 H), 7.07 (m, 1 H), 7.02 (m, 1 H), 6.97 (m, 2 H), 6.90 (m, 1 H), 6.45 (s, 1 H), 5.46 (br. s, 1 H), 4.17-4.09 (m, 2 H), 4.00 (m, 1 H), 2.81 (m, 1 H), 2.24-2.12 (m, 2 H), 1.93-1.80 (m, 7 H), 1.68-1.44 (m, 11 H), 1.38-0.76 (m, 14 H) ppm.
^{13}C NMR (100 MHz, $CDCl_3$): δ = 188.6, 170.4, 166.0, 163.0, 154.7, 141.6, 132.2, 130.5 (2 C), 130.3, 125.9, 122.2, 121.5 (2 C), 120.0, 114.7, 107.8, 83.2, 63.9, 53.8, 52.3, 33.1, 32.4, 30.5, 30.0, 29.1, 29.0, 26.4, 26.0, 25.9, 25.9, 25.2, 25.2, 25.2 (2 C), 24.6, 24.3, 14.6 ppm.
IR (mineral oil): 3196, 1760, 1718, 1687, 1662 cm^{-1}.
Anal. Calcd (%) for $C_{39}H_{48}N_4O_6$: C 70.04; H 7.23; N 8.38. Found: C 70.16; H 7.20; N 8.44.

1,3-Dicyclohexyl-8-(cyclohexylamino)-6-(2-hydroxyphenyl)-9-(4-methoxybenzoyl)-1,3,6-triazaspiro[4.4]non-8-ene-2,4,7-trione (3d)
Yield: 291 mg (91%); white solid; mp 275–277 °C.
^{1}H NMR (400 MHz, $CDCl_3$): δ = 7.71 (m, 2 H), 7.23 (m, 1 H), 7.08-6.97 (m, 4 H), 6.90 (m, 1 H), 6.46 (br. s, 1 H), 5.48 (d, 1 H, *J* = 12.0 Hz), 4.00 (m, 1 H), 3.89 (s, 3 H), 2.81 (m, 1 H), 2.18 (m, 2 H), 1.91-1.82 (m, 7 H), 1.68-1.43 (m, 7 H), 1.37-0.75 (m, 15 H) ppm.
^{13}C NMR (100 MHz, $CDCl_3$): δ = 188.5, 170.4, 166.0, 163.6, 154.7, 152.3, 132.4, 130.5 (2 C), 130.3, 125.9, 122.2, 121.5, 120.0, 114.2 (2 C), 107.7, 83.0, 55.6, 53.8, 53.7, 52.3, 33.1, 32.4, 30.5, 30.0, 29.1, 29.0, 26.4, 26.0, 25.9, 25.9, 25.2, 25.2, 25.1, 24.6, 24.3 ppm.
IR (mineral oil): 3376, 3282, 1764, 1723, 1700 cm^{-1}.
Anal. Calcd (%) for $C_{38}H_{46}N_4O_5$: C 69.70; H 7.08; N 8.56. Found: C 69.79; H 7.03; N 8.61.

9-Benzoyl-6-(5-chloro-2-hydroxyphenyl)-1,3-dicyclohexyl-8-(cyclohexylamino)-1,3,6-triazaspiro[4.4]non-8-ene-2,4,7-trione (3e)
Yield: 297 mg (90%); white solid; mp 240–242 °C.
^{1}H NMR (400 MHz, DMSO-d_6): δ = 10.23 (s, 1 H), 7.66 (m, 3 H), 7.57 (m, 2 H), 7.30 (m, 1 H), 7.00 (m, 1 H), 6.90 (m, 1 H), 3.85 (m, 1 H), 3.01 (m, 1 H), 2.16-2.02 (m, 2 H), 1.84-1.72 (m, 5 H), 1.66-1.13 (m, 17 H), 1.07-0.83 (m, 6 H), 0.69 (m, 1 H), 0.35 (m, 1 H) ppm.
^{13}C NMR (100 MHz, DMSO-d_6): δ = 189.6, 170.1, 163.5, 153.9, 153.2, 142.9, 139.8, 132.7, 129.7, 128.9 (2 C), 127.9, 125.8, 121.8 (2 C), 121.5, 118.2, 104.4, 82.3, 54.4, 51.9, 50.9, 31.4, 30.8, 30.2, 29.4, 28.9, 28.7, 25.7, 25.2, 25.1, 24.9, 24.8 (2 C), 24.4, 24.3, 24.2 ppm.
IR (mineral oil): 3362, 3280, 1767, 1723, 1700 cm^{-1}.
Anal. Calcd (%) for $C_{37}H_{43}ClN_4O_5$: C 67.41; H 6.58; N 8.50. Found: C 67.53; H 6.62; N 8.54.

1,3-Dicyclohexyl-8-(cyclohexylamino)-6-(2-hydroxyphenyl)-9-(4-methylbenzoyl)-1,3,6-triazaspiro[4.4]non-8-ene-2,4,7-trione (3f)

Yield: 313 mg (98%); white solid; mp 159–190 °C.

^{1}H NMR (400 MHz, DMSO-d_6): δ = 9.82 (s, 1 H), 7.58 (m, 2 H), 7.38 (m, 2 H), 7.22 (m, 1 H), 6.98 (m, 1 H), 6.88 (m, 1 H), 6.80 (m, 1 H), 6.76 (m, 1 H), 3.83 (m, 1 H), 3.01 (m, 1 H), 2.39 (s, 3 H), 2.15-1.97 (m, 2 H), 1.83-1.72 (m, 5 H), 1.66-1.41 (m, 7 H), 1.35-0.99 (m, 13 H), 0.96-0.84 (m, 2 H), 0.68 (m, 1 H), 0.39 (m, 1 H) ppm.

^{13}C NMR (100 MHz, DMSO-d_6): δ = 189.4, 170.2, 163.6, 154.0 (2 C), 143.0 (2 C), 137.4, 129.8, 129.3 (2 C), 127.9 (2 C), 126.2, 120.5, 118.8, 116.6, 104.6, 82.4, 54.1, 51.9, 50.8, 31.4, 31.1, 30.0, 29.4, 28.7 (2 C), 25.7, 25.3, 25.2, 25.1, 24.8 (2 C), 24.5, 24.3, 24.2, 21.0 ppm.

IR (mineral oil): 3354, 3277, 1787, 1724, 1700 cm^{-1}.

Anal. Calcd (%) for $C_{38}H_{46}N_4O_5$: C 71.45; H 7.26; N 8.77. Found: C 71.37; H 7.23; N 8.85.

1,3-Dicyclohexyl-8-(cyclohexylamino)-6-(2-hydroxyphenyl)-9-(4-nitrobenzoyl)-1,3,6-triazaspiro[4.4]non-8-ene-2,4,7-trione (3g)

Yield: 301 mg (90%); white solid; mp 164–166 °C.

^{1}H NMR (400 MHz, DMSO-d_6): δ = 9.89 (s, 1 H), 8.40 (m, 2 H), 7.86 (m, 2 H), 7.32 (m, 1 H), 7.24 (m, 1 H), 6.99 (m, 1 H), 6.88 (m, 1 H), 6.81 (m, 1 H), 3.83 (m, 1 H), 3.04 (m, 1 H), 2.16-1.95 (m, 2 H), 1.81-1.70 (m, 5 H), 1.65-1.09 (m, 19 H), 1.01-0.74 (m, 4 H), 0.34 (br. s, 1 H) ppm.

^{13}C NMR (100 MHz, DMSO-d_6): δ = 187.5, 170.1, 163.0, 154.0, 153.9, 149.5, 144.7, 144.3, 130.0, 129.4 (2 C), 126.2, 124.2 (2 C), 120.3, 118.9, 116.7, 82.2, 55.6, 51.84, 50.89, 31.53, 30.1, 29.5, 28.7 (2 C), 25.8, 25.3, 25.2, 24.9, 24.9, 24.8, 24.4 (2 C), 24.4, 24.3 ppm.

IR (mineral oil): 3313, 3270, 1782, 1727, 1700 cm^{-1}.

Anal. Calcd (%) for $C_{37}H_{43}N_5O_7$: C 66.35; H 6.47; N 10.46. Found: C 66.44; H 6.53; N 10.58.

9-(4-Chlorobenzoyl)-1,3-dicyclohexyl-8-(cyclohexylamino)-6-(2-hydroxyphenyl)-1,3,6-triazaspiro[4.4]non-8-ene-2,4,7-trione (3h)

Yield: 297 mg (90%); white solid; mp 173–175 °C.

^{1}H NMR (400 MHz, $CDCl_3$): δ = 7.65 (m, 2 H), 7.49 (m, 2 H), 7.24 (m, 1 H), 7.04 (m, 2 H), 6.92 (m, 1 H), 6.38 (s, 1 H), 5.77 (br. s, 1 H), 3.97 (m, 1 H), 2.81 (m, 1 H), 2.21-2.09 (m, 2 H), 1.95-1.80 (m, 7 H), 1.68-1.46 (m, 9 H), 1.36-0.85 (m, 13 H) ppm.

^{13}C NMR (100 MHz, $CDCl_3$): δ = 188.4, 170.1, 165.5, 154.6, 152.3, 139.2, 138.1, 130.5, 129.5 (2 C), 129.3 (2 C), 126.0, 122.0, 121.6, 120.0, 106.8, 83.1, 53.8, 52.3, 32.9, 32.4, 32.4, 30.5, 30.1, 29.1, 29.0, 26.4, 26.0, 25.9, 25.9, 25.2, 25.2, 25.1, 24.5, 24.3 ppm.
IR (mineral oil): 3379, 3278, 1767, 1724, 1701, 1633 cm^{-1}.
Anal. Calcd (%) for $C_{37}H_{43}ClN_4O_5$: C 67.41; H 6.58; N 8.50. Found: C 67.53; H 6.61; N 8.47.

1,3-Dicyclohexyl-6-(2-hydroxyphenyl)-8-(isopropylamino)-9-(4-methylbenzoyl)-1,3,6-triazaspiro[4.4]non-8-ene-2,4,7-trione (3i)
Yield: 281 mg (94%); white solid; mp 146–148 °C.
^{1}H NMR (400 MHz, $CDCl_3$): δ = 7.62 (m, 2 H), 7.28 (m, 1 H), 7.21 (m, 1 H), 7.07 (m, 1 H), 7.00 (m, 1 H), 6.89 (m, 1 H), 6.58 (br. s, 1 H), 5.43 (d, 1 H, *J* = 12.0 Hz), 3.98 (m, 1 H), 3.30 (m, 1 H), 2.83 (m, 1 H), 2.43 (s, 3 H), 2.22-2.10 (m, 2 H), 1.95-1.65 (m, 8 H), 1.56-1.50 (m, 2 H), 1.40-0.88 (m, 15 H) ppm.
^{13}C NMR (100 MHz, $CDCl_3$): δ = 189.4, 170.2, 165.8, 154.6, 152.4, 143.7, 136.9, 130.3, 129.6 (2 C), 128.3 (2 C), 126.0, 122.0, 121.4, 119.8, 108.0, 83.1, 52.3, 46.8, 30.5, 30.0, 29.0 (2 C), 26.4, 26.0, 25.9 (2 C), 25.9, 25.2, 25.2, 22.7, 22.3, 21.6 ppm.
IR (mineral oil): 3197, 1760, 1718, 1687 cm^{-1}.
Anal. Calcd (%) for $C_{35}H_{42}N_4O_5$: C 70.21; H 7.07; N 9.36. Found: C 70.45; H 7.10; N 9.30.

1,3-Dicyclohexyl-6-(2-hydroxyphenyl)-8-(isopropylamino)-9-(4-methoxybenzoyl)-1,3,6-triazaspiro[4.4]non-8-ene-2,4,7-trione (3j)
Yield: 280 mg (91%); white solid; mp 148–150 °C.
^{1}H NMR (400 MHz, $CDCl_3$): δ = 7.74 (m, 2 H), 7.23 (m, 1 H), 7.09 (m, 2 H), 7.00 (m, 2 H), 6.90 (m, 1 H), 6.47 (br. s, 1 H), 5.34 (d, 1 H, *J* = 8.0 Hz), 3.99 (m, 1 H), 3.83 (s, 3 H), 3.27 (m, 1 H), 2.80 (m, 1 H), 2.22-2.12 (m, 2 H), 1.92-1.65 (m, 8 H), 1.54-1.49 (m, 2 H), 1.40-0.83 (m, 14 H) ppm.
^{13}C NMR (100 MHz, $CDCl_3$): δ = 188.4, 170.3, 166.0, 163.6, 154.7, 152.3, 142.0, 132.0, 130.6 (2 C), 130.3, 125.9, 122.2, 121.5, 120.0, 114.2 (2 C), 108.5, 83.2, 55.5, 53.8, 52.3, 47.0, 30.5, 30.0, 29.0 (2 C), 26.4, 26.0, 25.9, 25.9, 25.2, 25.2, 22.8, 22.3 ppm.
IR (mineral oil): 3183, 3060, 1783, 1760, 1739, 1698 cm^{-1}.
Anal. Calcd (%) for $C_{35}H_{42}N_4O_6$: C 68.38; H 6.89; N 9.11. Found: C 68.46; H 6.92; N 9.08.

9-(4-Chlorobenzoyl)-1,3-dicyclohexyl-6-(2-hydroxyphenyl)-8-(isopropylamino)-1,3,6-triazaspiro[4.4]non-8-ene-2,4,7-trione (3k)
Yield: 285 mg (92%); white solid; mp 151–153 °C.

^{1}H NMR (400 MHz, $CDCl_3$): δ = 7.67 (m, 2 H), 7.47 (m, 2 H), 7.23 (m, 1 H), 7.06 (m, 1 H), 7.01 (m, 1 H), 6.90 (m, 1 H), 6.45 (br. s, 1 H), 5.67 (d, 1 H, *J* = 4.0 Hz), 3.96 (m, 1 H), 3.29 (m, 1 H), 2.80 (m, 1 H), 2.21-2.07 (m, 2 H), 1.95-1.66 (m, 7 H), 1.59-1.51 (m, 2 H), 1.39-0.85 (m, 15 H) ppm.
^{13}C NMR (100 MHz, $CDCl_3$): δ = 188.3, 170.0, 165.4, 154.5, 152.3, 139.3, 137.8, 130.5, 129.5 (2 C), 129.3 (2 C), 125.9, 122.0, 121.5, 119.9, 107.4, 83.0, 53.8, 52.3, 47.22, 30.5, 30.1, 29.0, 29.0, 26.4, 26.0, 25.9, 25.8, 25.2, 25.2, 22.7, 22.3 ppm.
IR (mineral oil): 3281, 3080, 1774, 1721, 1704 cm^{-1}.
Anal. Calcd (%) for $C_{34}H_{39}ClN_4O_5$: C 65.96; H 6.35; N 9.05. Found: C 66.04; H 6.30; N 9.11.

9-Benzoyl-1,3-dicyclohexyl-6-(2-hydroxyphenyl)-8-(phenylamino)-1,3,6-triazaspiro[4.4]non-8-ene-2,4,7-trione (3l)
Yield: 285 mg (92%); yellow solid; mp 187–189 °C.
^{1}H NMR (400 MHz, DMSO-d_6): δ = 9.86 (s, 1 H), 9.31 (s, 1 H), 7.35 (m, 2 H), 7.31–7.23 (m, 2 H), 7.17 (m, 2 H), 7.02 (m, 1 H), 6.97–6.89 (m, 3 H), 6.84 (m, 1 H), 6.74 (m, 3 H), 3.89 (m, 1 H), 3.08 (m, 1 H), 2.20-2.02 (m, 2 H), 1.88–0.85 (m, 18 H) ppm.
^{13}C NMR (100 MHz, DMSO-d_6): δ = 189.7, 169.8, 163.8, 154.1, 153.8, 141.8, 140.7, 138.3, 132.1, 129.9, 128.5 (2 C), 128.0, 127.8 (2 C), 126.2, 123.6, 121.3 (2 C), 120.4, 119.0, 116.8, 113.8, 108.5, 82.3, 52.0, 51.0, 30.0, 29.4, 28.8, 28.7, 25.7, 25.3, 25.2, 24.9, 24.8 (2 C) ppm.
IR (mineral oil): 3398, 3247, 1768, 1723, 1704 cm^{-1}.
Anal. Calcd (%) for $C_{37}H_{38}N_4O_5$: C 71.83; H 6.19; N 9.06. Found: C 72.15; H 6.07; N 9.10.

1,3-Dicyclohexyl-9-(4-ethoxybenzoyl)-6-(2-hydroxyphenyl)-8-(phenylamino)-1,3,6-triazaspiro[4.4]non-8-ene-2,4,7-trione (3m)
Yield: 312 mg (94%); yellow solid; mp 187–189 °C.
^{1}H NMR (400 MHz, DMSO-d_6): δ = 9.85 (s, 1 H), 9.20 (s, 1 H), 7.34 (m, 2 H), 7.24 (m, 1 H), 7.01 (m, 1 H), 6.93 (m, 3 H), 6.83 (m, 1 H), 6.75 (m, 3 H), 6.66 (m, 2 H), 4.09 (q, *J* 6.8 Hz, 2 H), 3.88 (m, 1 H), 3.05 (m, 1 H), 2.20-2.02 (m, 2 H), 1.88–0.85 (m, 21 H) ppm.
^{13}C NMR (100 MHz, DMSO-d_6): δ = 188.2, 169.9, 163.9, 161.7, 154.1, 153.8, 140.7, 140.5, 131.1, 130.3 (2 C), 129.9, 128.2 (2 C), 126.2, 123.4, 121.2 (2 C), 120.5, 118.9, 116.7, 113.7 (2 C), 109.2, 82.3, 63.2, 52.0, 51.0, 30.0, 29.5, 28.8, 28.7, 25.7, 25.3, 25.2, 25.0, 24.8, 24.8, 14.3 ppm.
IR (mineral oil): 3354, 3250, 1771, 1720, 1705 cm^{-1}.
Anal. Calcd (%) for $C_{39}H_{42}N_4O_6$: C 70.68; H 6.39; N 8.45. Found: C 70.89; H 6.43; N 9.01.

8-(Butylamino)-1,3-dicyclohexyl-9-(4-ethoxybenzoyl)-6-(2-hydroxyphenyl)-1,3,6-triazaspiro[4.4]non-8-ene-2,4,7-trione (3n)

Yield: 270 mg (84%) (purity 90%); white solid; mp 130–132 °C.

^{1}H NMR (400 MHz, DMSO-d_6): δ = 9.81 (s, 1 H), 7.64 (m, 2 H), 7.22 (m, 2 H), 7.13–6.77 (m, 10 H), 4.16–4.06 (m, 3 H), 3.83 (m, 1 H), 3.15 (m, 1 H), 2.98 (m, 2 H), 2.55 (m, 1 H), 2.40 (m, 1 H), 2.15-1.96 (m, 3 H), 1.87–0.67 (m, 18 H) ppm.

^{13}C NMR (100 MHz, DMSO-d_6): δ = 188.4, 170.3, 163.7, 162.1, 154.1, 153.8, 143.9, 132.5, 130.4 (2 C), 129.9, 126.2, 120.6, 118.9, 116.7, 114.3 (2 C), 105.0, 82.4, 63.5, 51.9, 50.8, 45.5, 40.6, 32.2, 30.5, 30.2, 29.5, 28.8, 25.8, 25.4, 24.9, 19.2, 19.1, 14.4, 13.6, 13.3 ppm.

IR (mineral oil): 3325, 3175, 1773, 1718, 1642 cm^{-1}.

Anal. Calcd (%) for $C_{37}H_{46}N_4O_6$: C 69.14; H 7.21; N 8.72. Found: C 68.89; H 7.25; N 8.71.

NMR charts of compounds 3a-n

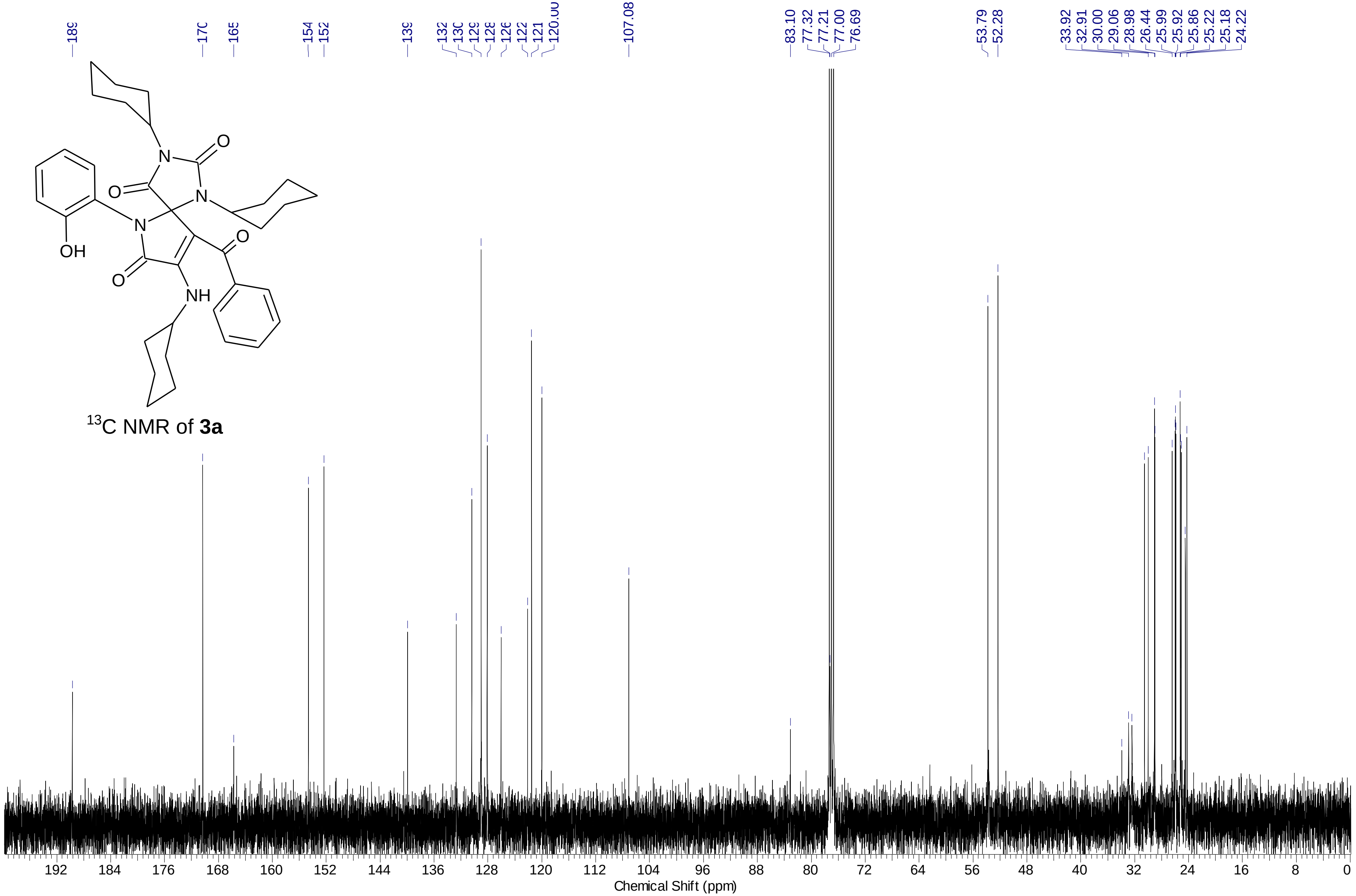

^{13}C NMR of **3a**

MAN6727.001.esp

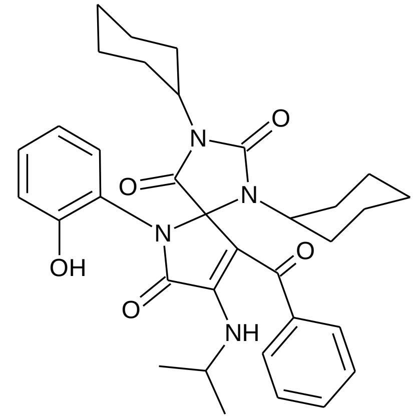

^{1}H NMR of **3b**

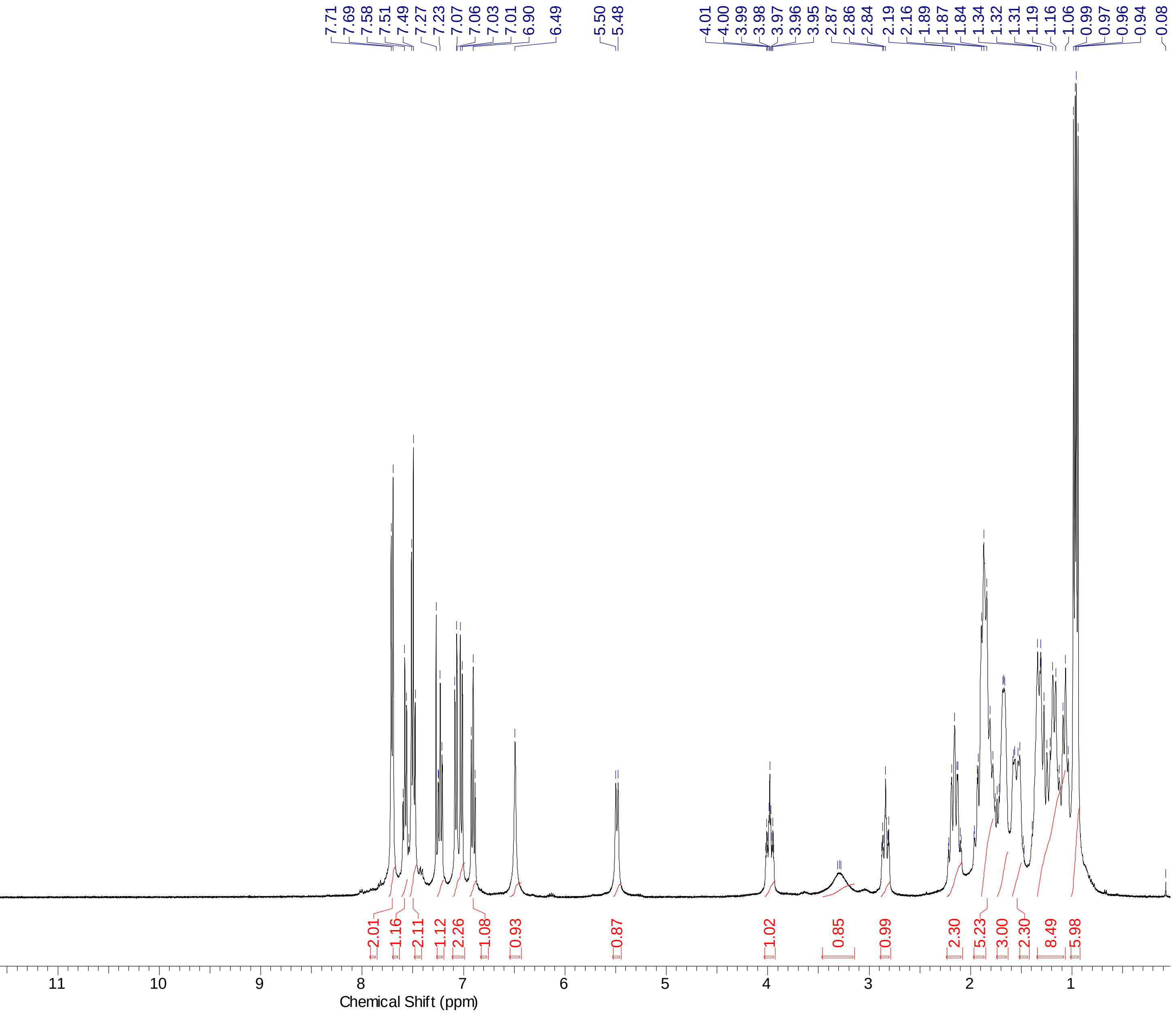

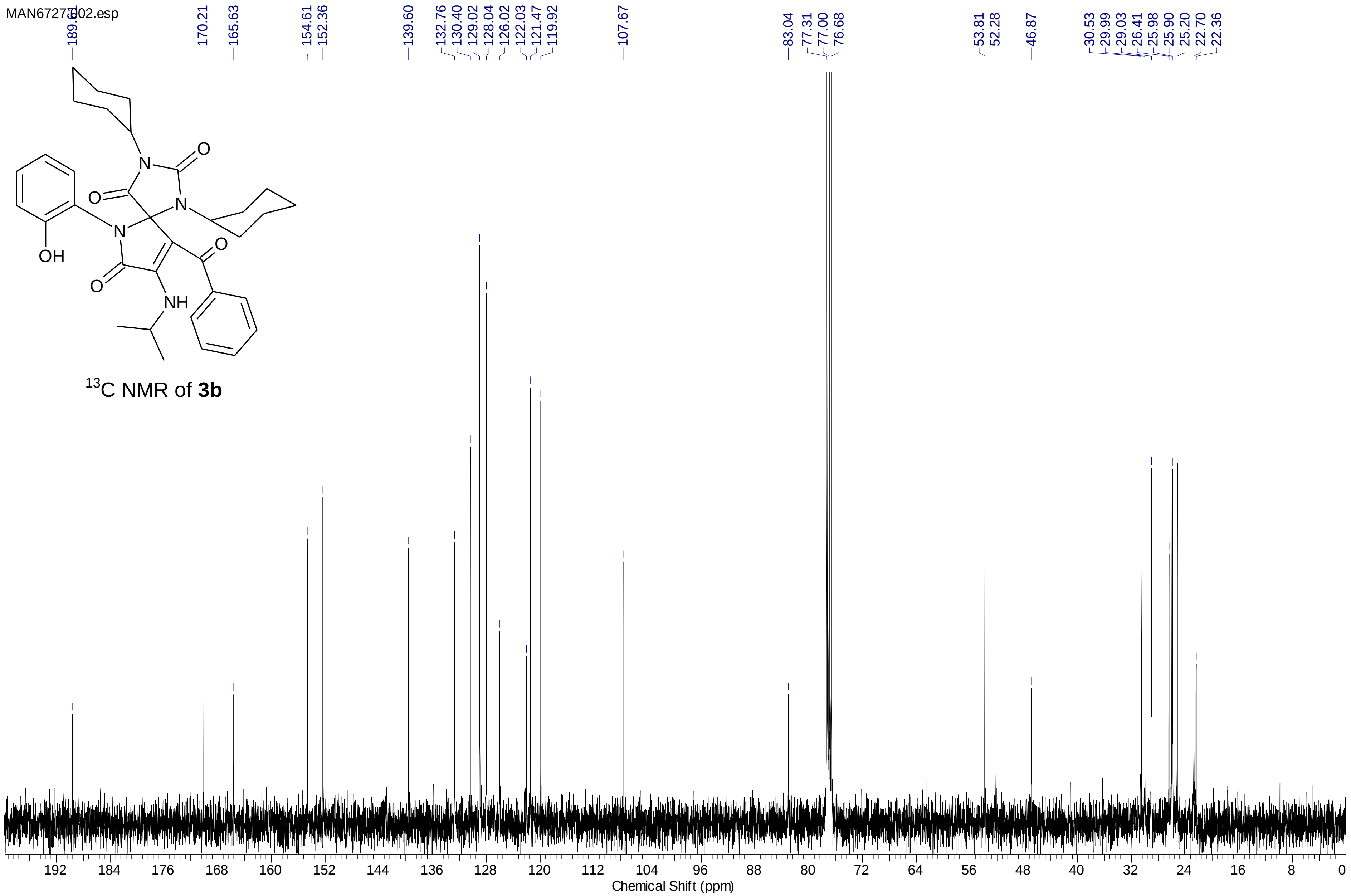
189.61
170.21
165.63
154.61
152.36
139.60
132.76
130.40
129.02
128.04
126.02
122.03
121.47
119.92
107.67
83.04
77.31
77.00
76.68
53.81
52.28
46.87
30.53
29.99
29.03
26.41
25.98
25.90
25.20
22.70
22.36
N
O
OH
NH
Chemical Shift (ppm)

^{13}C NMR of **3b**

MAN6698.001.esp

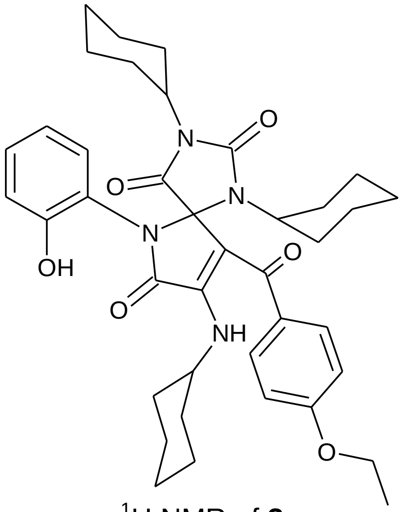

^{1}H NMR of **3c**

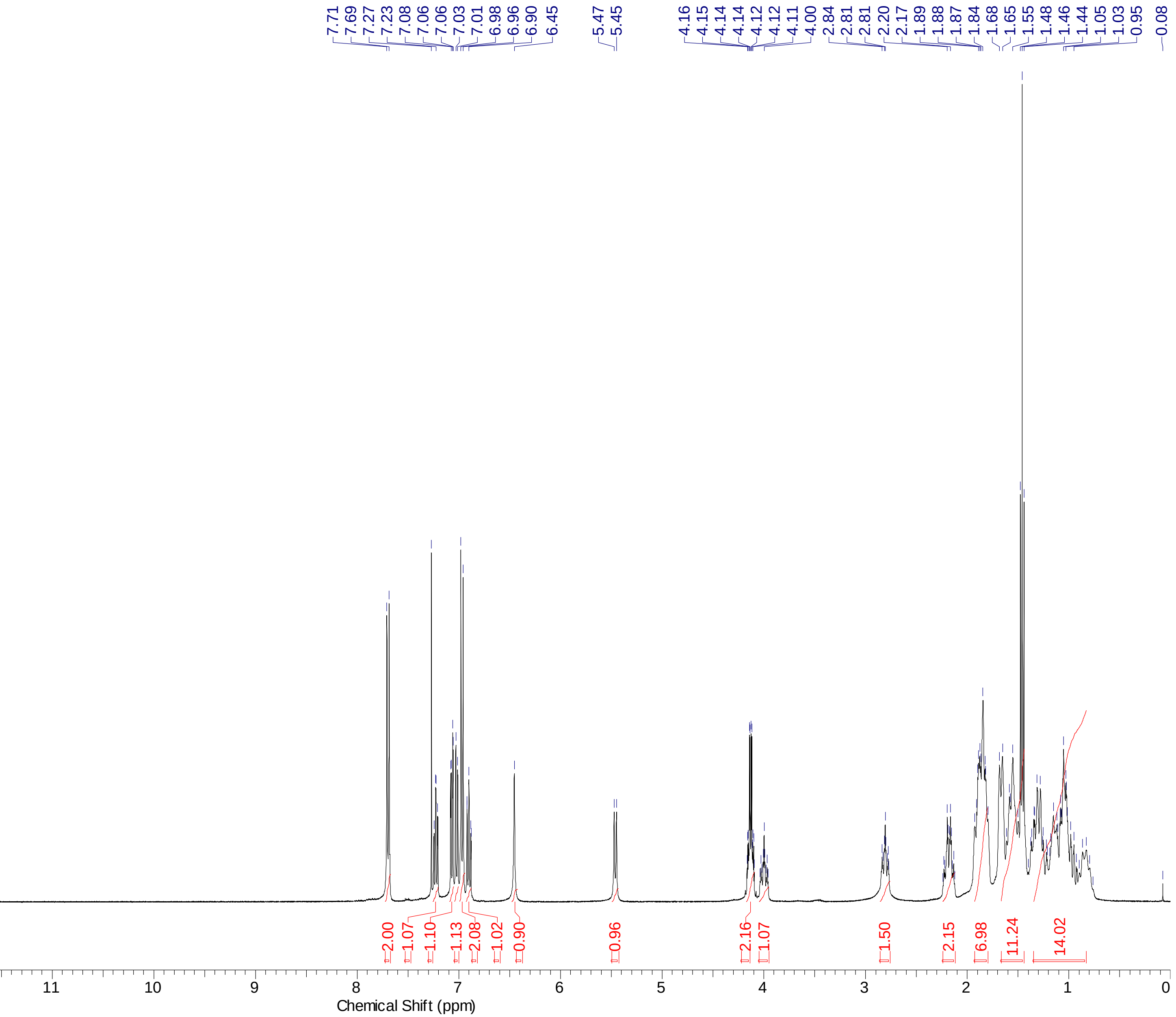

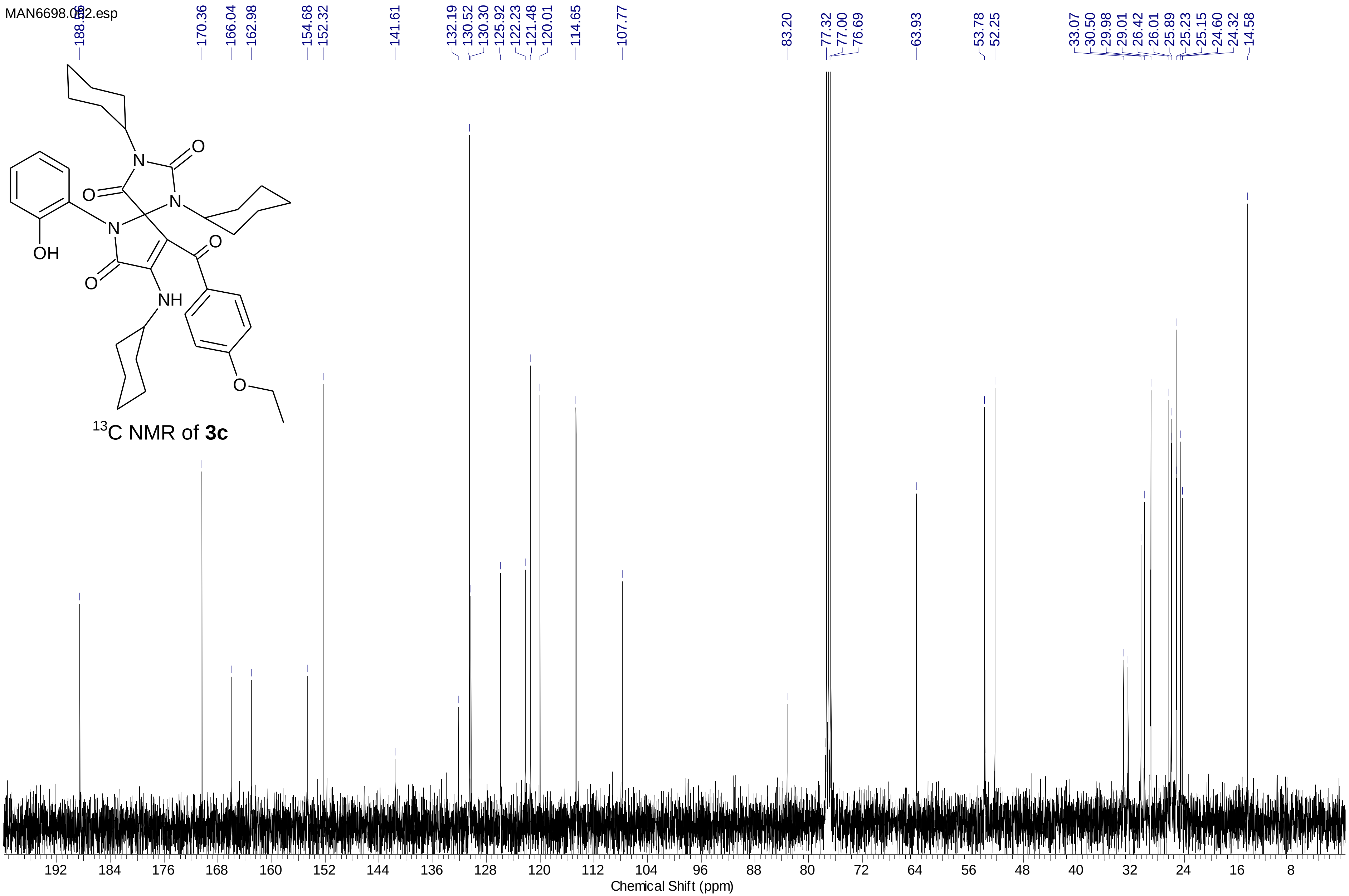

MAN6698.062.esp
188.36
170.36
166.04
162.98
154.68
152.32
141.61
132.19
130.52
130.30
125.92
122.23
121.48
120.01
114.65
107.77
83.20
77.32
77.00
76.69
63.93
53.78
52.25
33.07
30.50
29.98
29.01
26.42
26.01
25.89
25.23
25.15
24.60
24.32
14.58
N
O
N
O
N
OH
O
O
NH
O
192
184
176
168
160
152
144
136
128
120
112
104
96
88
80
72
64
56
48
40
32
24
16
8
Chemical Shift (ppm)

^{13}C NMR of **3c**

MAN5993.001.esp

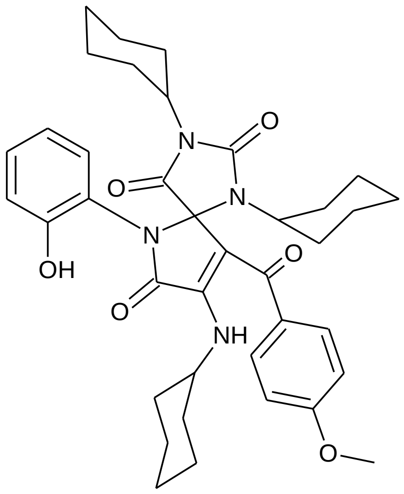

^{1}H NMR of **3d**

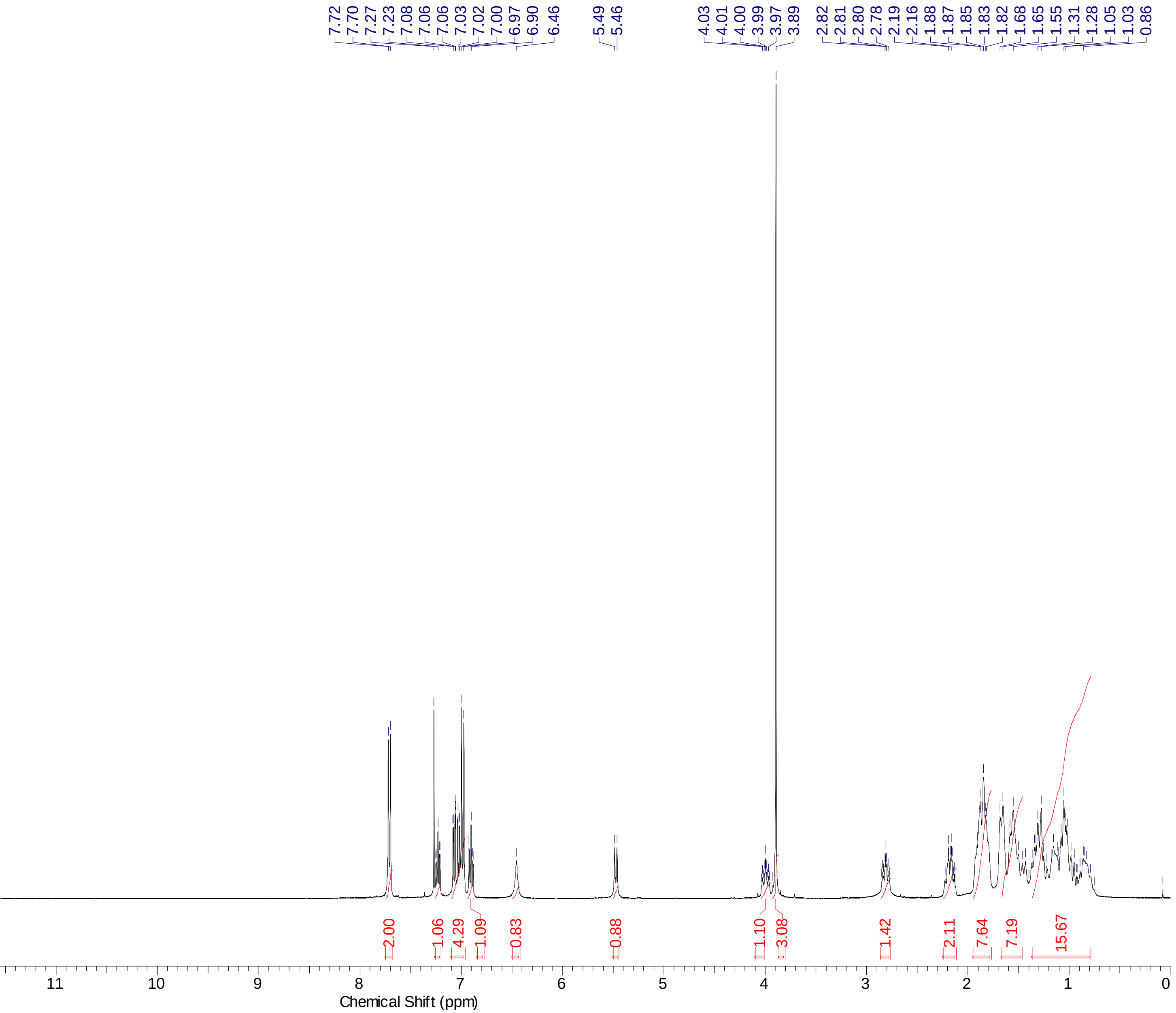

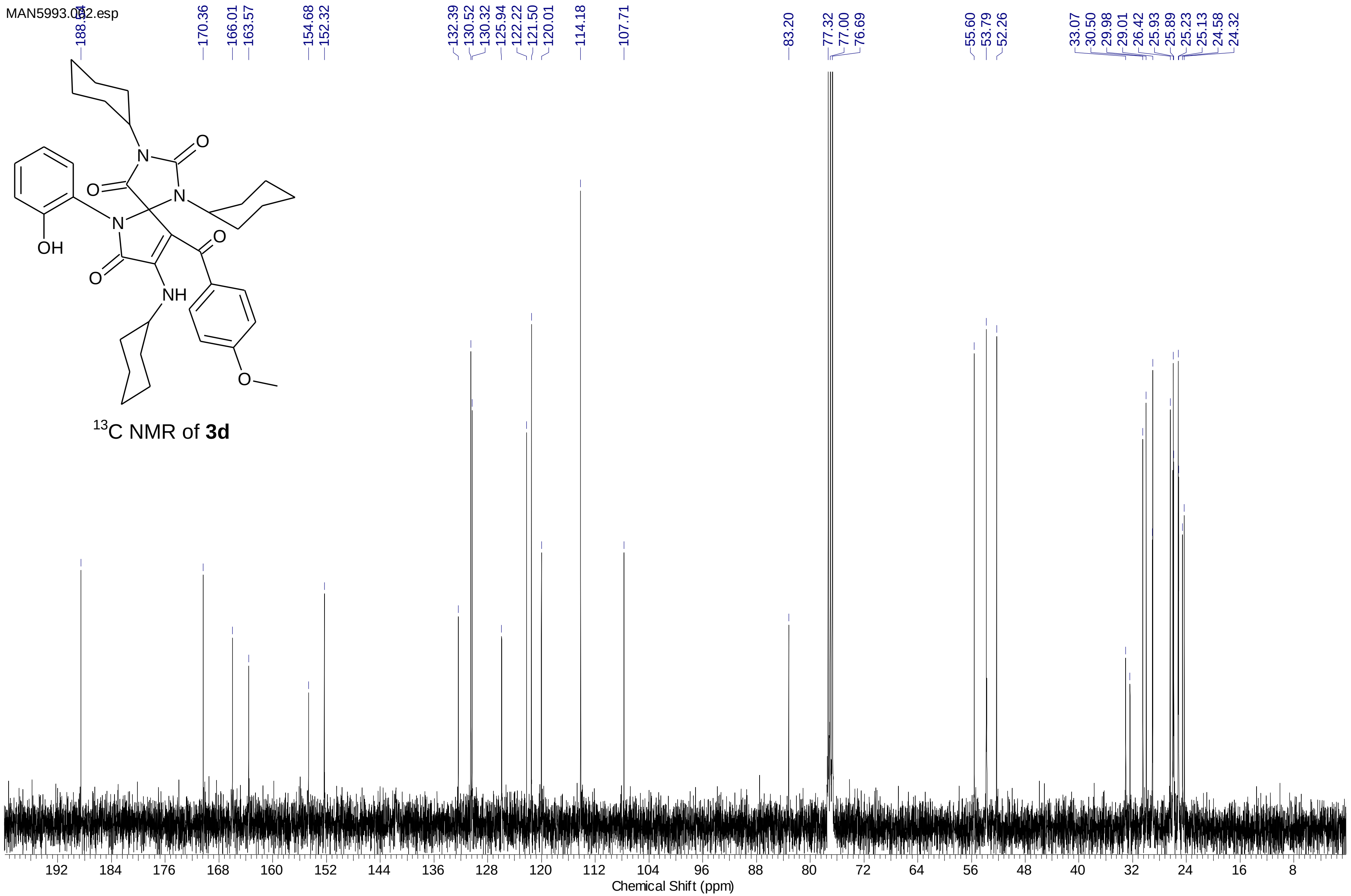
MAN5993.002.esp
188.54
170.36
166.01
163.57
154.68
152.32
132.39
130.52
130.32
125.94
122.22
121.50
120.01
114.18
107.71
83.20
77.32
77.00
76.69
55.60
53.79
52.26
33.07
30.50
29.98
29.01
26.42
25.93
25.89
25.23
25.13
24.58
24.32
O
N
O
N
OH
N
O
O
NH
O
192
184
176
168
160
152
144
136
128
120
112
104
96
88
80
72
64
56
48
40
32
24
16
8
Chemical Shift (ppm)

^{13}C NMR of **3d**

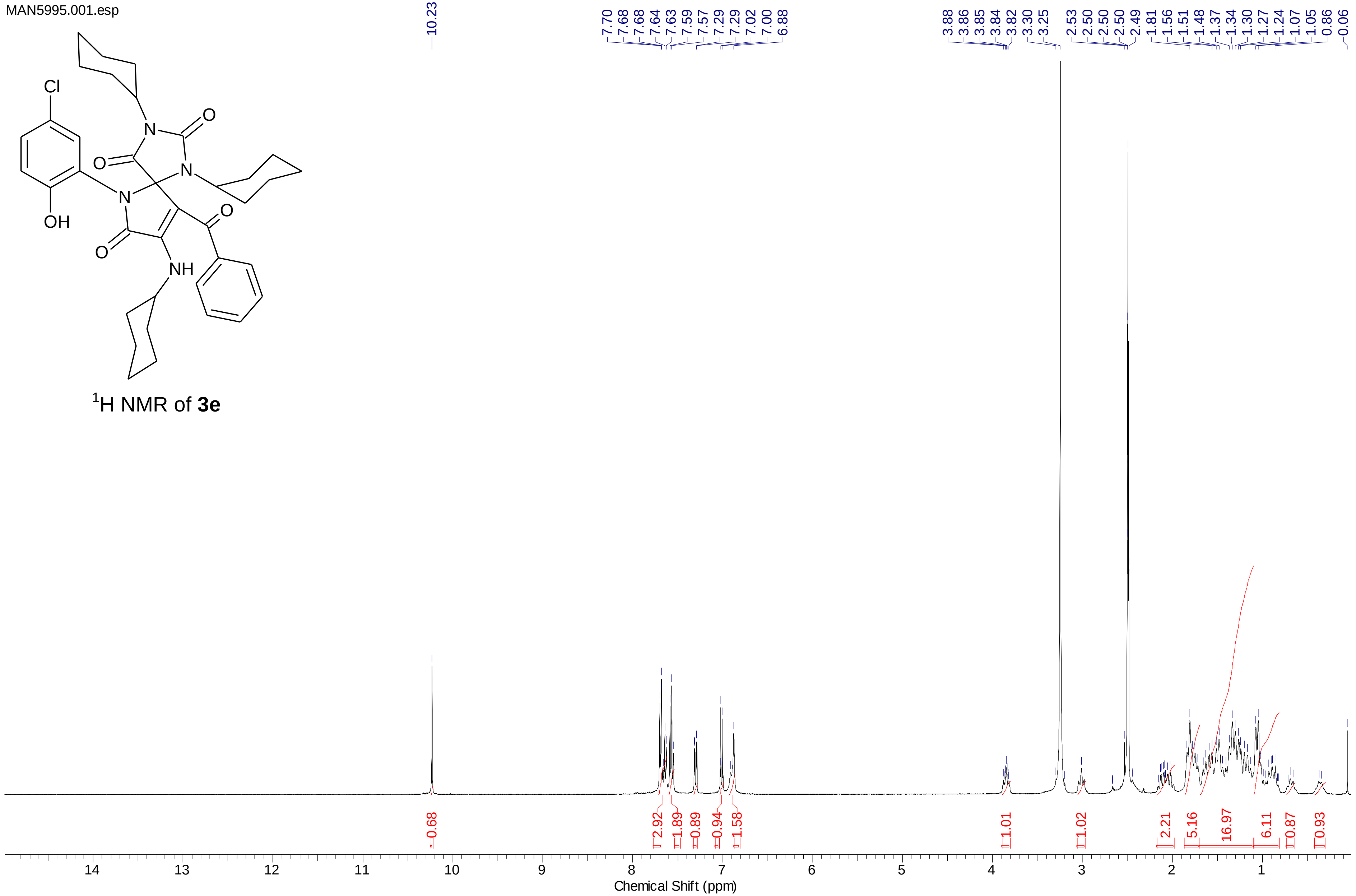
MAN5995.001.esp
Cl
O
N
O
N
N
O
OH
O
NH
10.23
7.70
7.68
7.68
7.64
7.63
7.59
7.57
7.29
7.29
7.02
7.00
6.88
3.88
3.86
3.85
3.84
3.82
3.30
3.25
2.53
2.50
2.50
2.50
2.49
1.81
1.56
1.51
1.48
1.37
1.34
1.30
1.27
1.24
1.07
1.05
0.86
0.06
0.68
2.92
1.89
0.89
0.94
1.58
1.01
1.02
2.21
5.16
16.97
6.11
0.87
0.93
14
13
12
11
10
9
8
7
6
5
4
3
2
1
Chemical Shift (ppm)

^{1}H NMR of **3e**

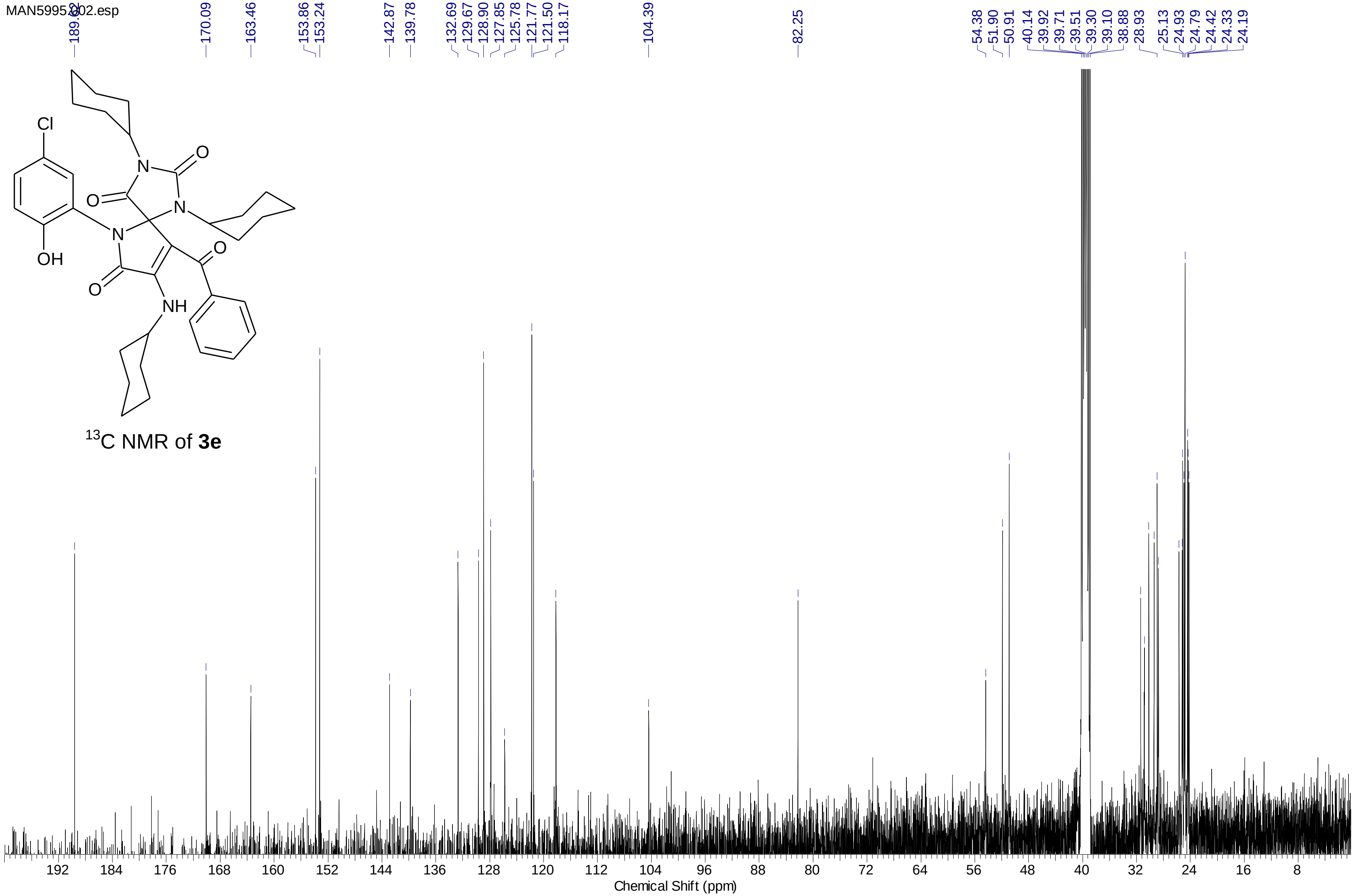
MAN5995.002.esp
189.02
170.09
163.46
153.86
153.24
142.87
139.78
132.69
129.67
128.90
127.85
125.78
121.77
121.50
118.17
104.39
82.25
54.38
51.90
50.91
40.14
39.92
39.71
39.51
39.30
39.10
38.88
28.93
25.13
24.93
24.79
24.42
24.33
24.19
Cl
O
N
O
N
N
OH
O
O
NH
Chemical Shift (ppm)

^{13}C NMR of **3e**

MAN5996.001.esp

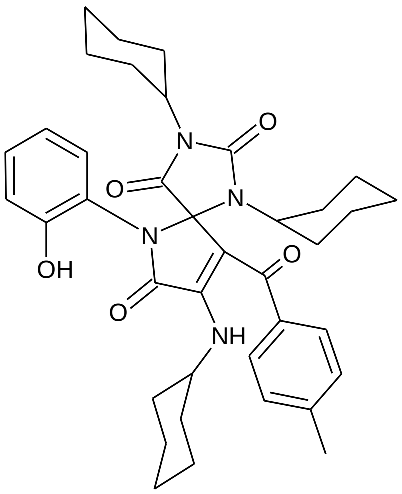

^{1}H NMR of **3f**

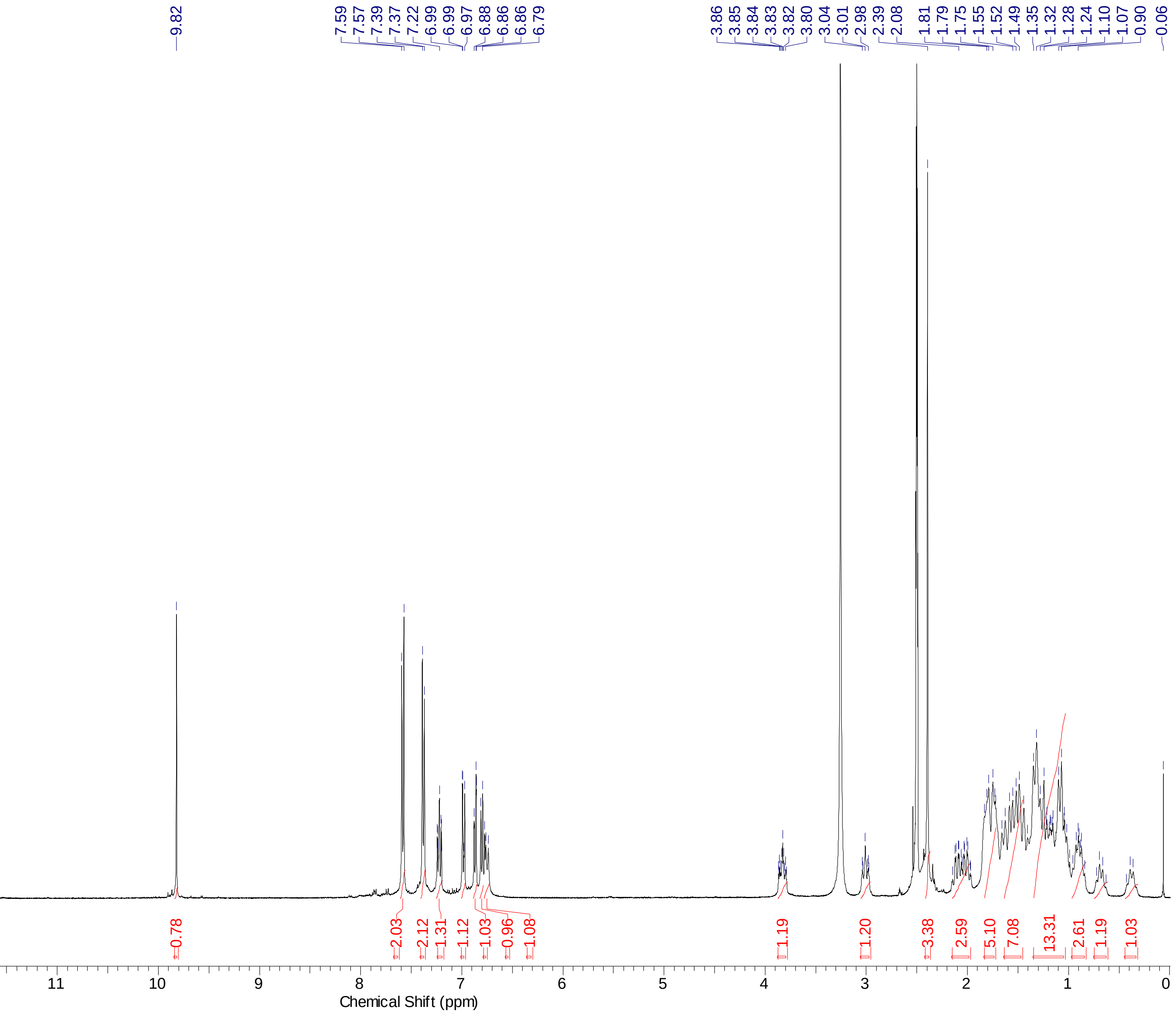

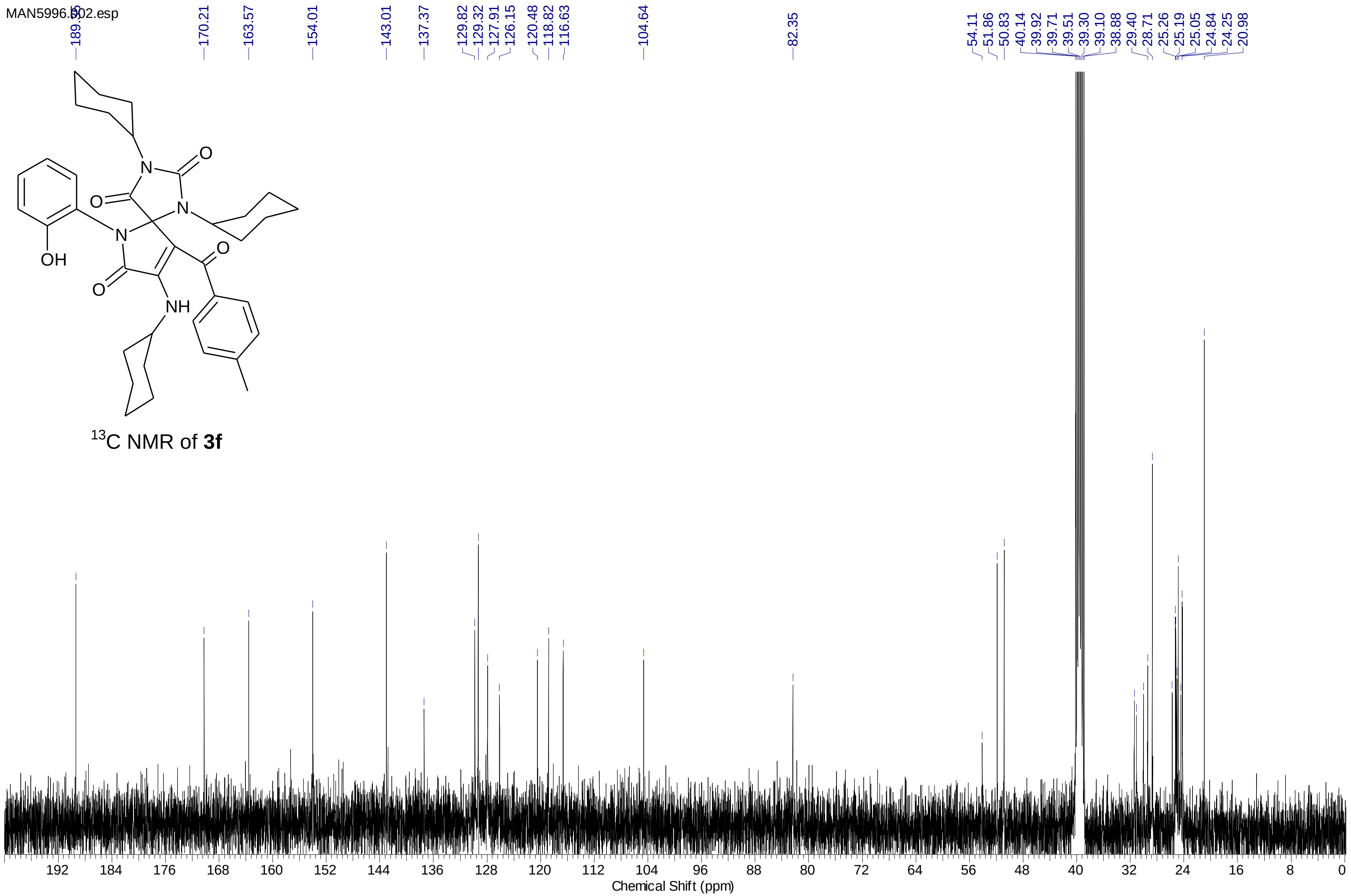
189.35
170.21
163.57
154.01
143.01
137.37
129.82
129.32
127.91
126.15
120.48
118.82
116.63
104.64
82.35
54.11
51.86
50.83
40.14
39.92
39.71
39.51
39.30
39.10
38.88
29.40
28.71
25.26
25.19
25.05
24.84
24.25
20.98
O
N
O
N
OH
N
O
O
NH
^{13}C NMR of **3f**
192
184
176
168
160
152
144
136
128
120
112
104
96
88
80
72
64
56
48
40
32
24
16
8
0
Chemical Shift (ppm)

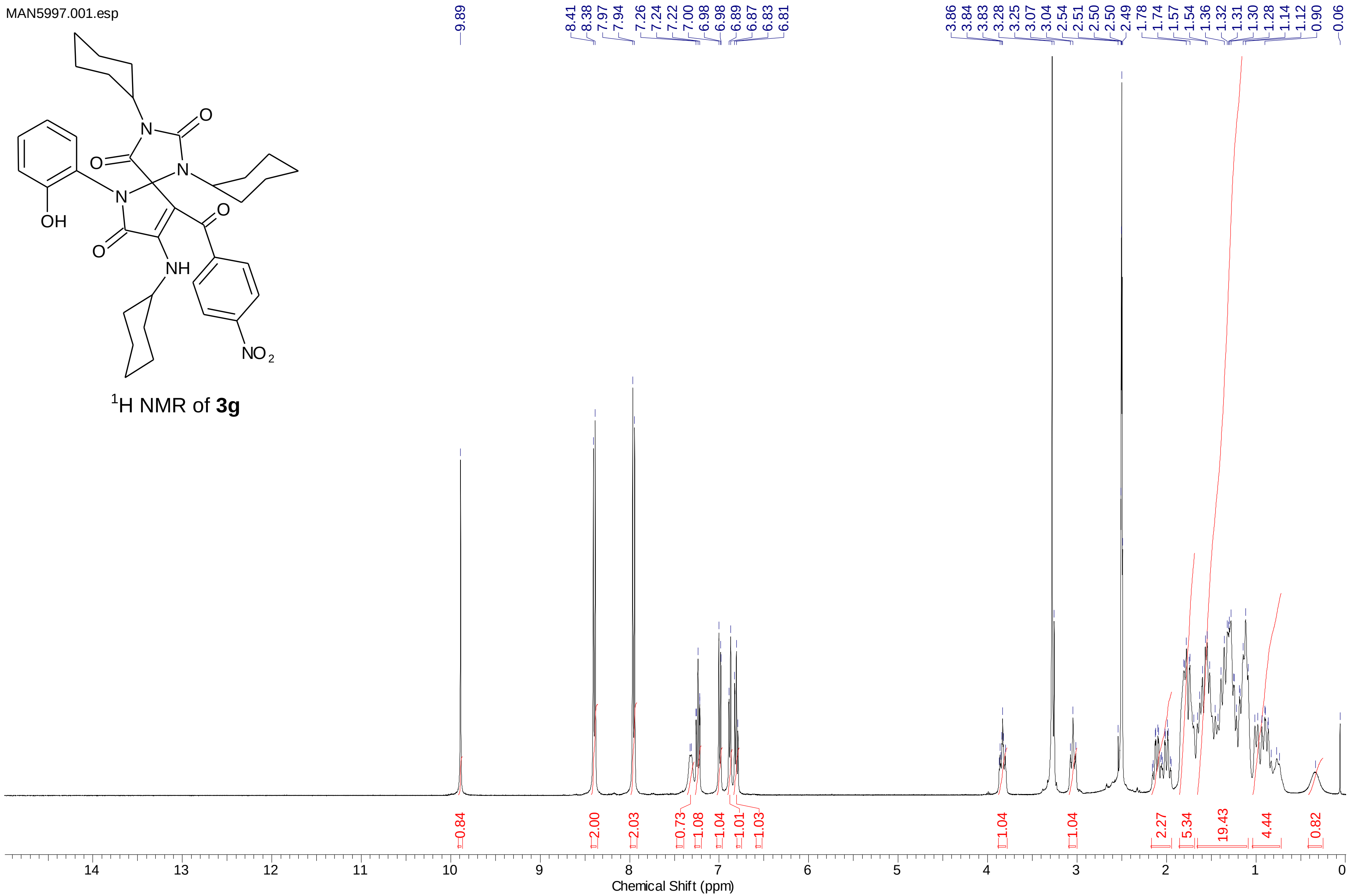

MAN5997.001.esp
9.89
8.41
8.38
7.97
7.94
7.26
7.24
7.22
7.00
6.98
6.98
6.89
6.87
6.83
6.81
3.86
3.84
3.83
3.28
3.25
3.07
3.04
2.54
2.51
2.50
2.50
2.49
1.78
1.74
1.57
1.54
1.36
1.32
1.31
1.30
1.28
1.14
1.12
0.90
0.06
O
N
O
N
N
OH
O
O
NH
NO2
0.84
2.00
2.03
0.73
1.08
1.04
1.01
1.03
1.04
1.04
2.27
5.34
19.43
4.44
0.82
14
13
12
11
10
9
8
7
6
5
4
3
2
1
0
Chemical Shift (ppm)

^{1}H NMR of **3g**

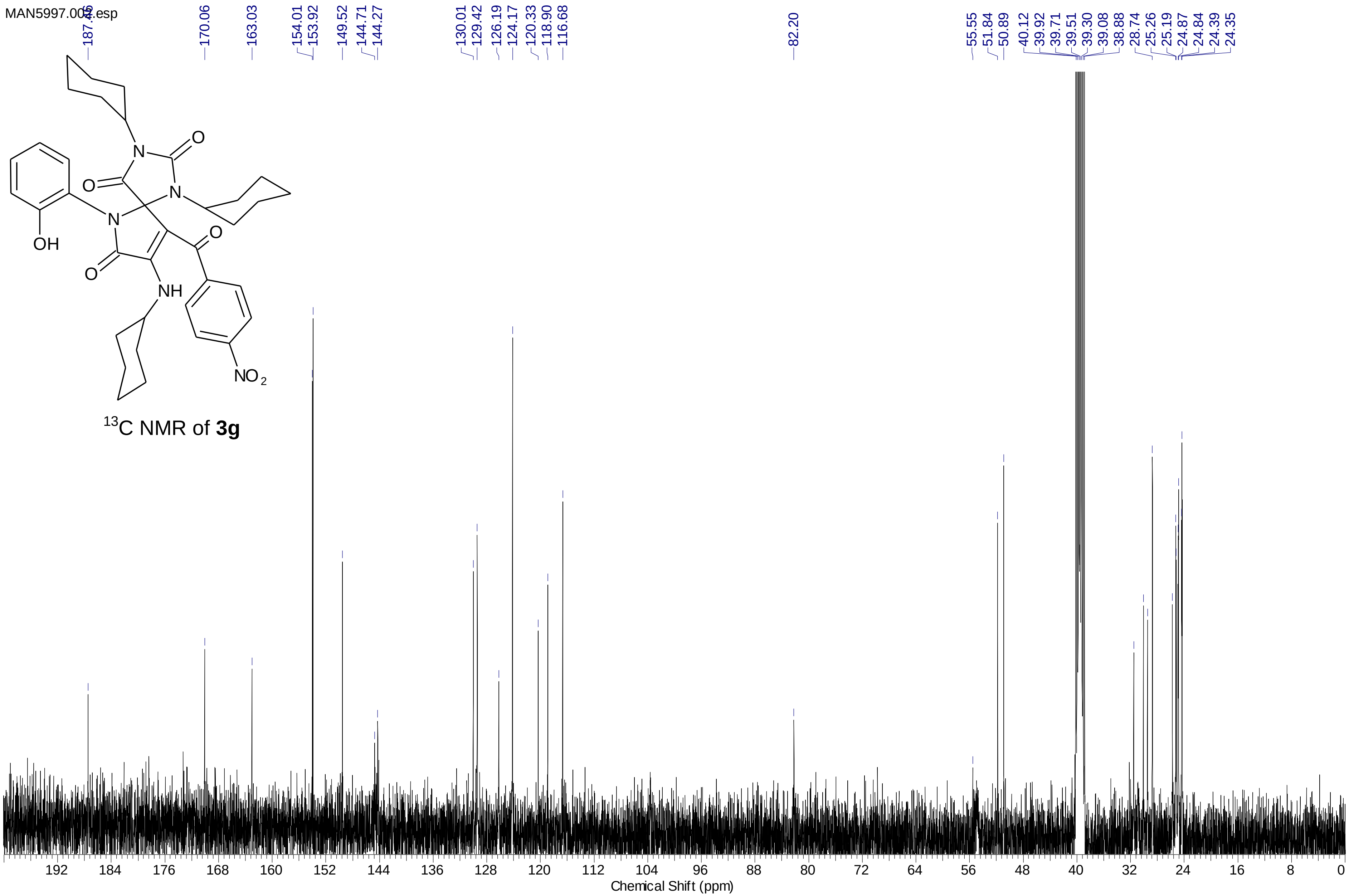
187.46
170.06
163.03
154.01
153.92
149.52
144.71
144.27
130.01
129.42
126.19
124.17
120.33
118.90
116.68
82.20
55.55
51.84
50.89
40.12
39.92
39.71
39.51
39.30
39.08
38.88
28.74
25.26
25.19
24.87
24.84
24.39
24.35
O
N
N
O
O
N
OH
O
O
NH
NO_2
192 184 176 168 160 152 144 136 128 120 112 104 96 88 80 72 64 56 48 40 32 24 16 8 0
Chemical Shift (ppm)

^{13}C NMR of **3g**

MAN3877.001.esp

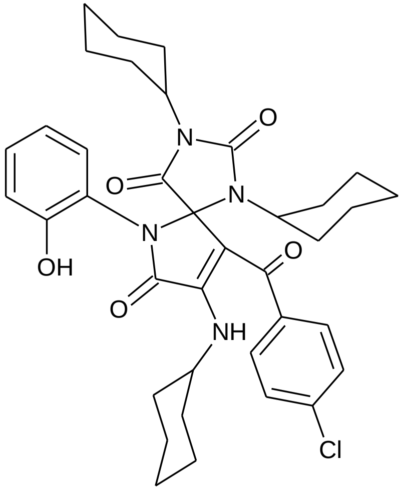

^{1}H NMR of **3h**

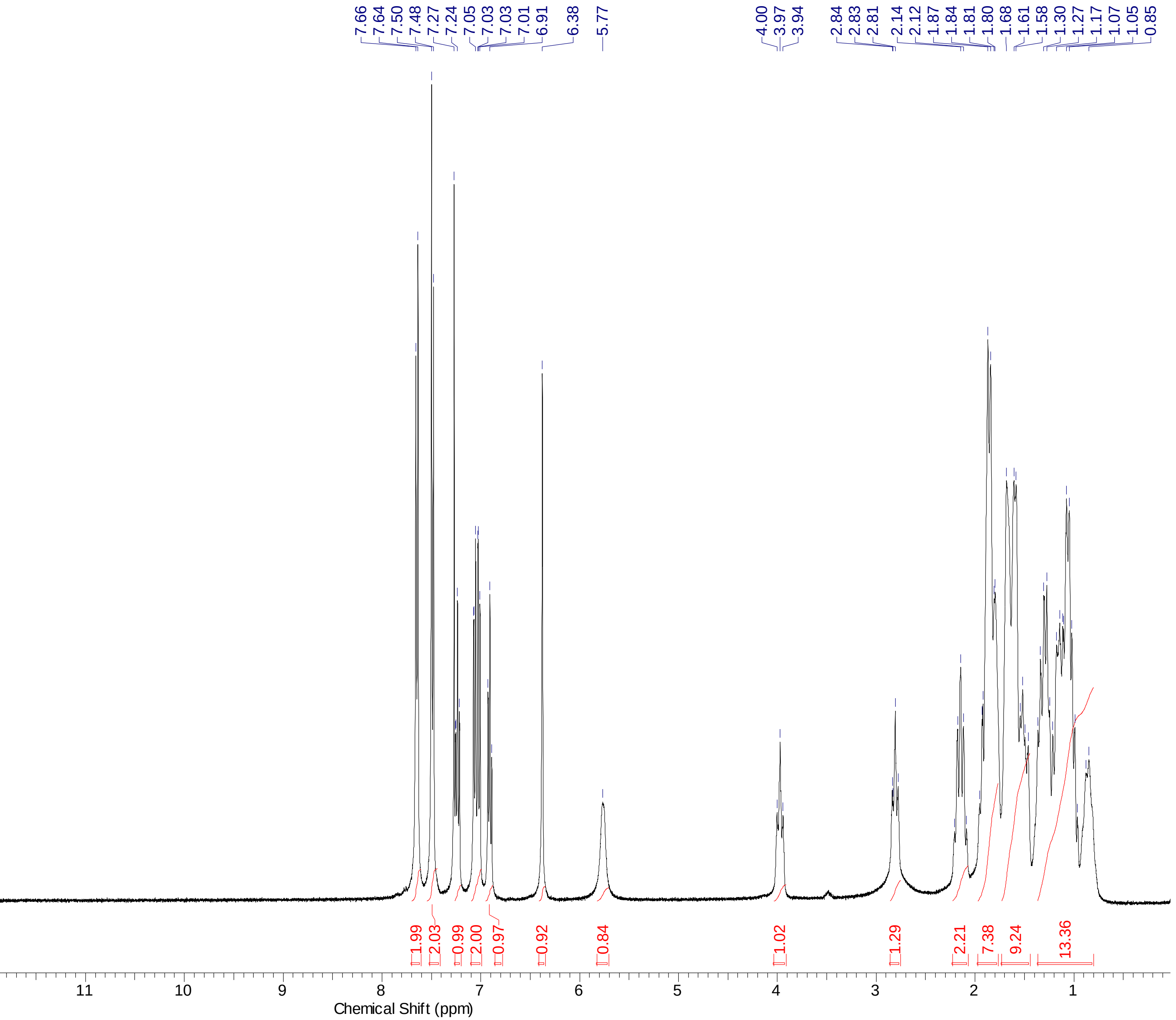

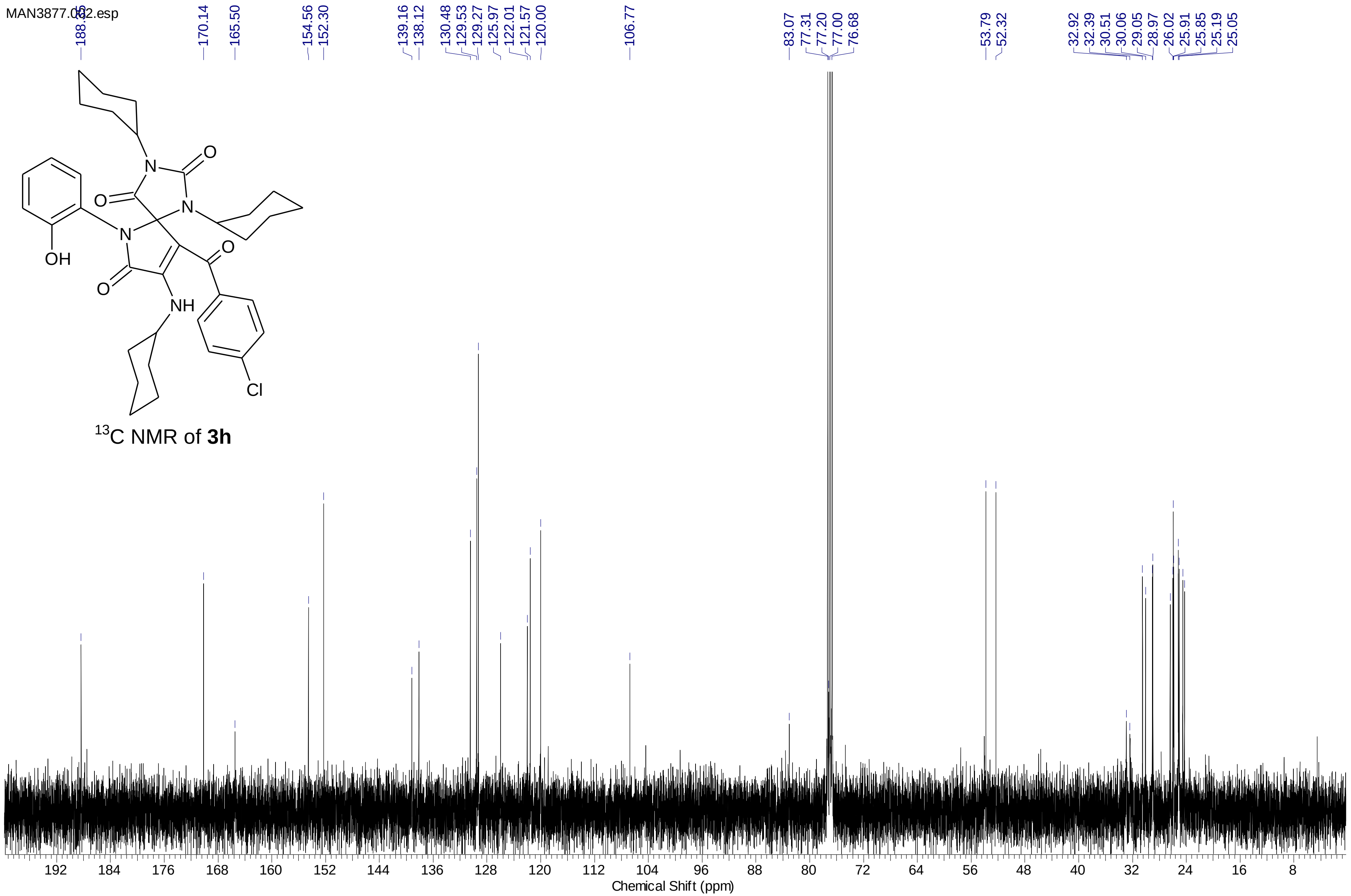
MAN3877.062.esp
170.14
165.50
154.56
152.30
139.16
138.12
130.48
129.53
129.27
125.97
122.01
121.57
120.00
106.77
83.07
77.31
77.20
77.00
76.68
53.79
52.32
32.92
32.39
30.51
30.06
29.05
28.97
26.02
25.91
25.85
25.19
25.05
O
N
N
N
OH
NH
Cl
192
184
176
168
160
152
144
136
128
120
112
104
96
88
80
72
64
56
48
40
32
24
16
8
Chemical Shift (ppm)

^{13}C NMR of **3h**

MAN6728.001.esp

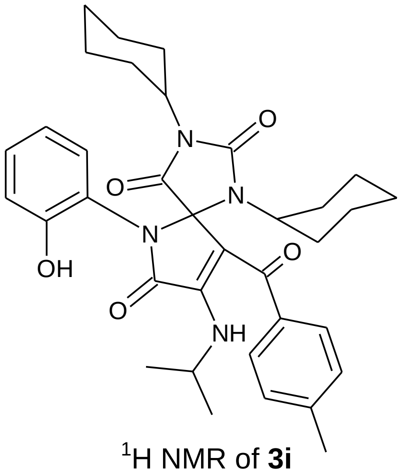

^{1}H NMR of **3i**

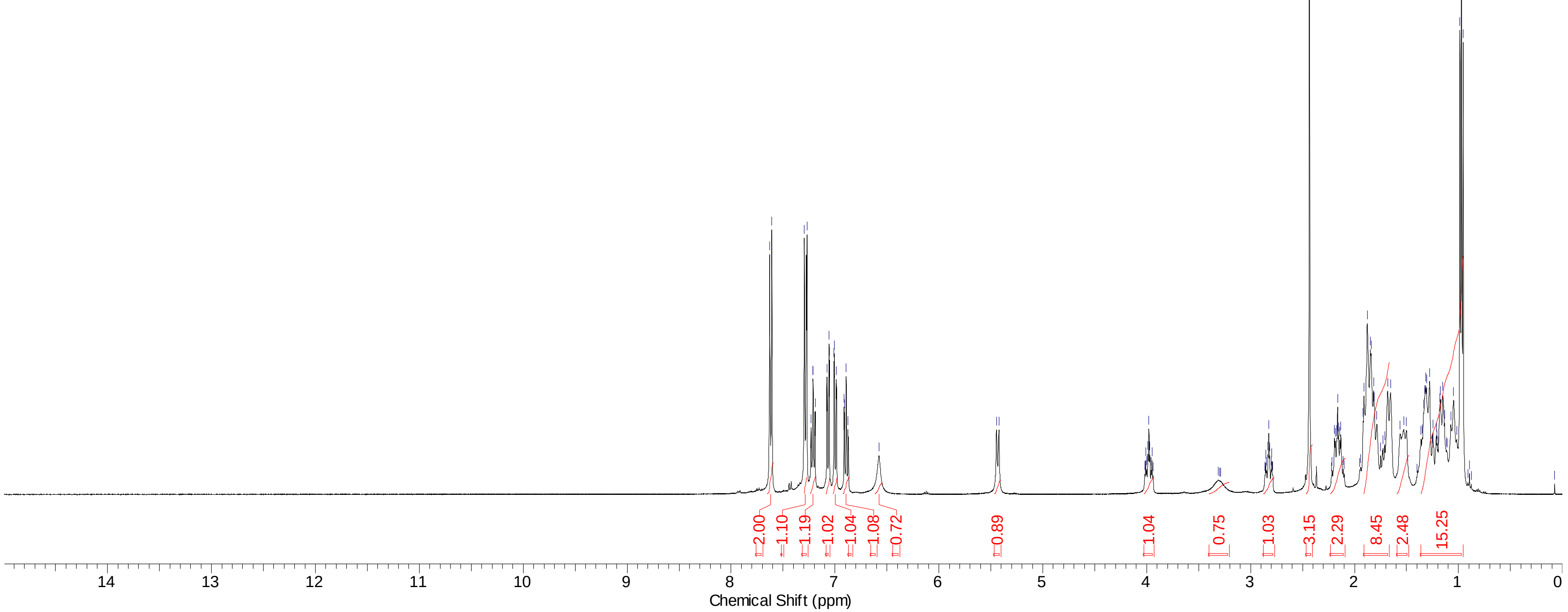

MAN6728.002.esp

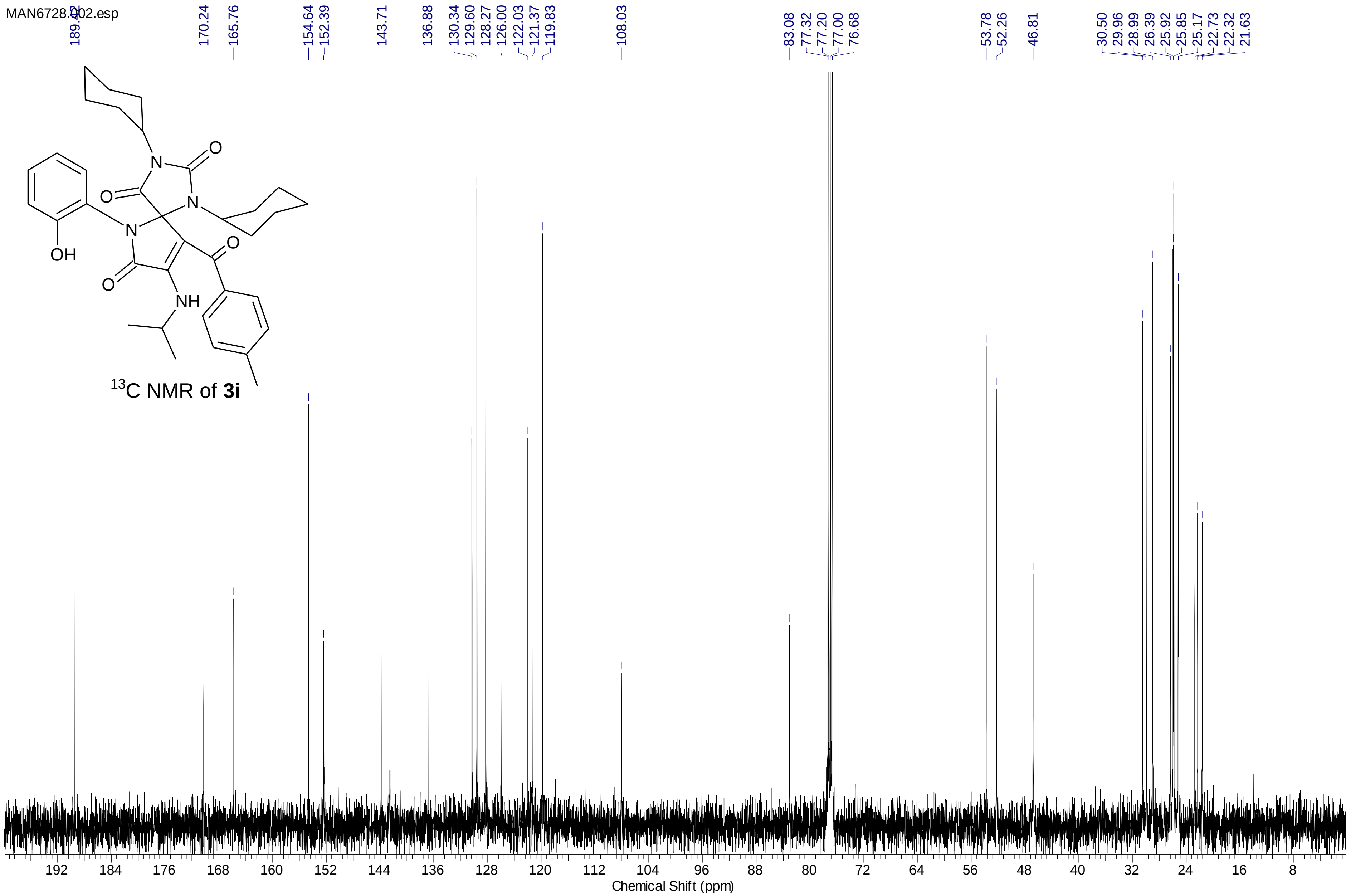

^{13}C NMR of **3i**

MAN6729.001.esp

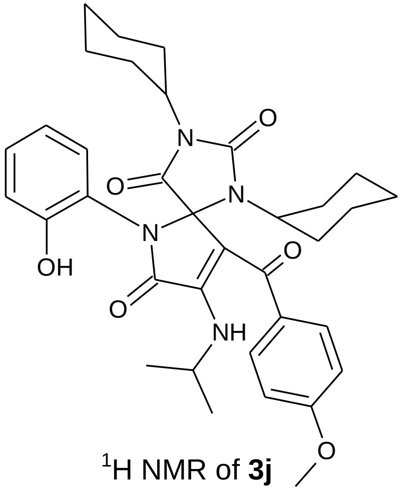

^{1}H NMR of **3j**

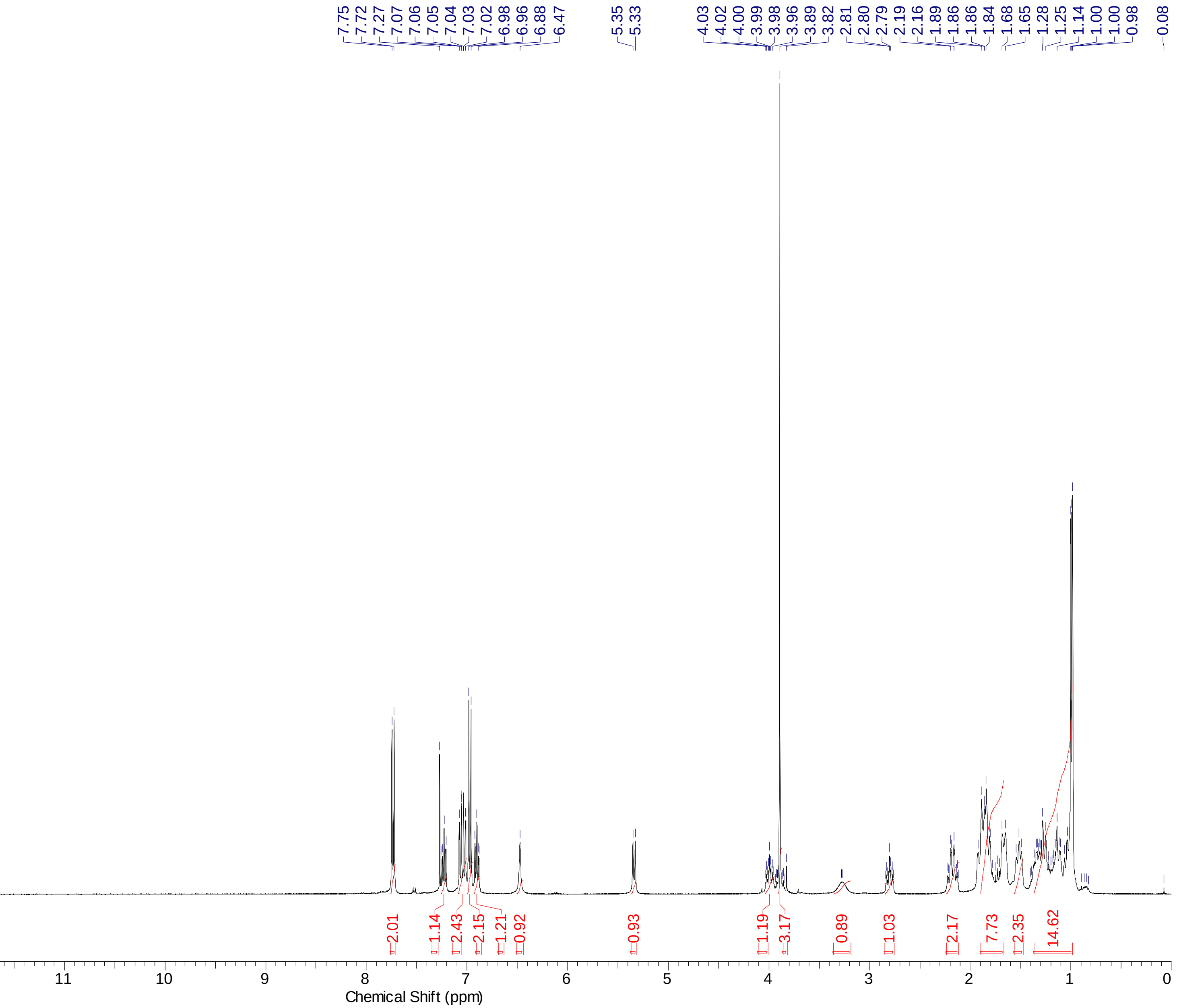

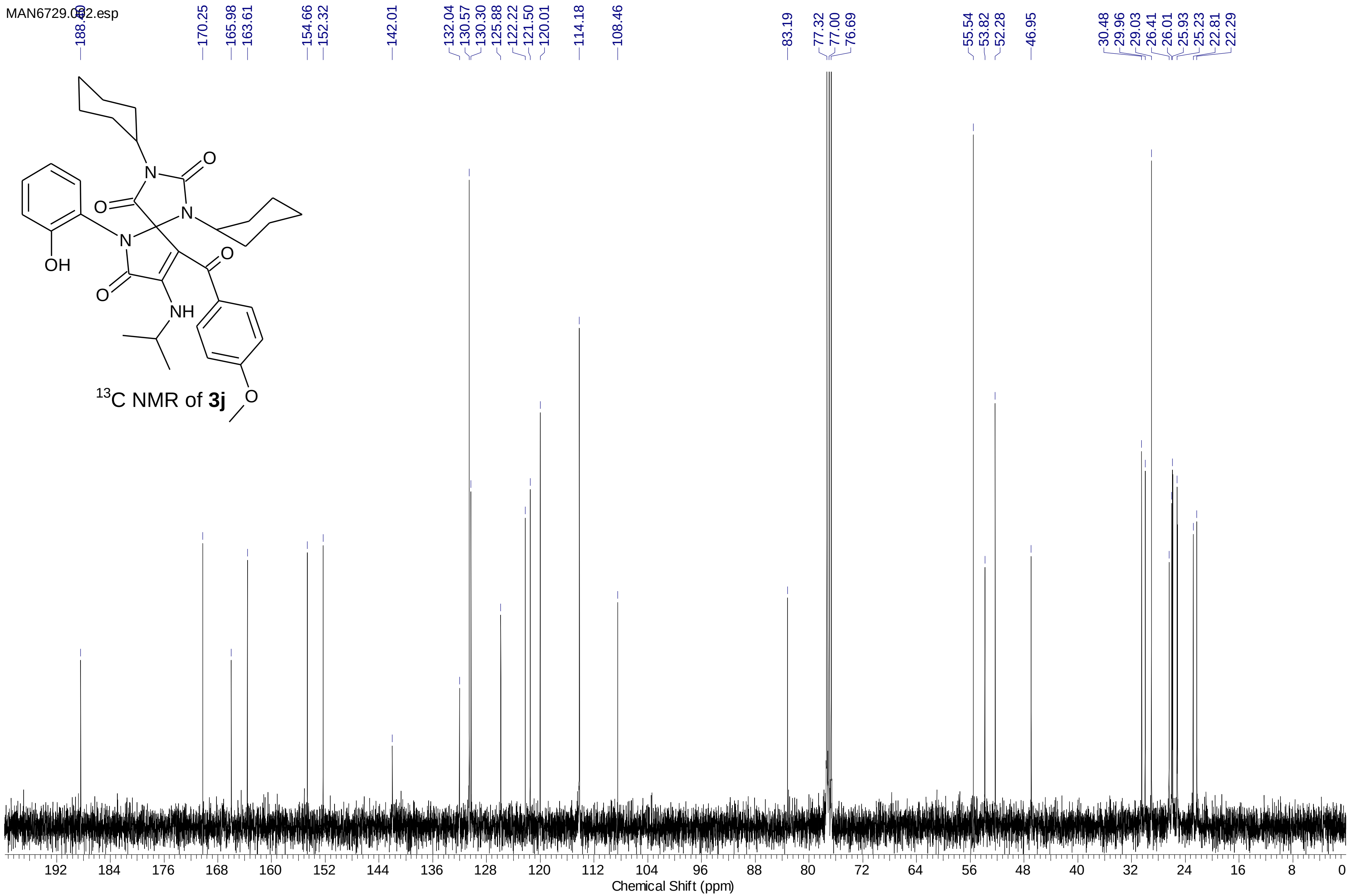
MAN6729.062.esp
170.25
165.98
163.61
154.66
152.32
142.01
132.04
130.57
130.30
125.88
122.22
121.50
120.01
114.18
108.46
83.19
77.32
77.00
76.69
55.54
53.82
52.28
46.95
30.48
29.96
29.03
26.41
26.01
25.93
25.23
22.81
22.29
O
N
N
N
OH
NH
O
^{13}C NMR of **3j**
192 184 176 168 160 152 144 136 128 120 112 104 96 88 80 72 64 56 48 40 32 24 16 8 0
Chemical Shift (ppm)

MAN6730.001.esp

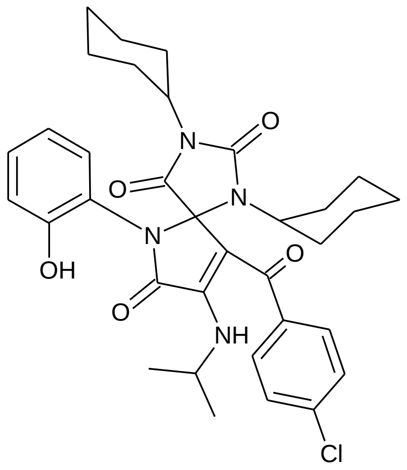

^{1}H NMR of **3k**

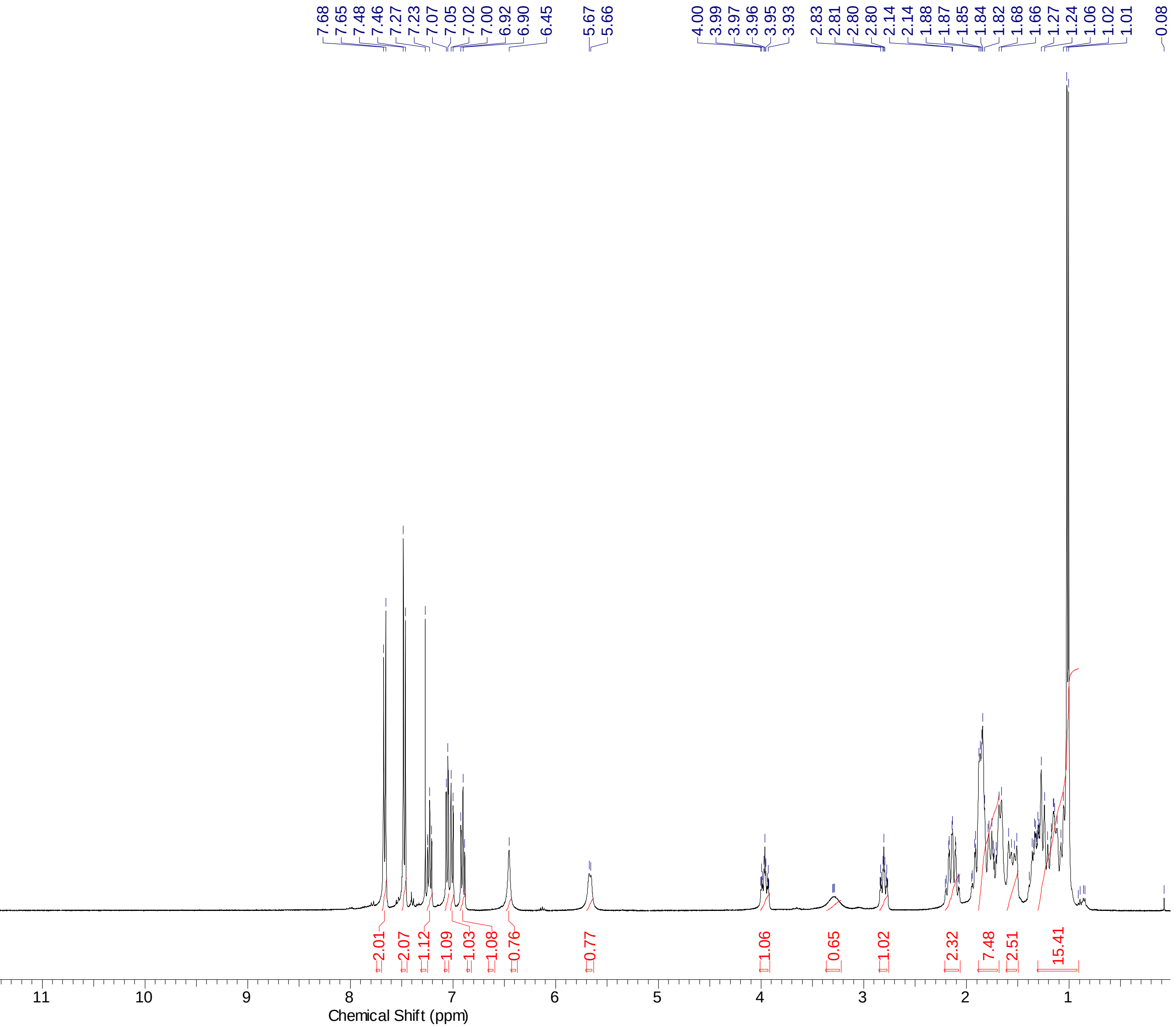

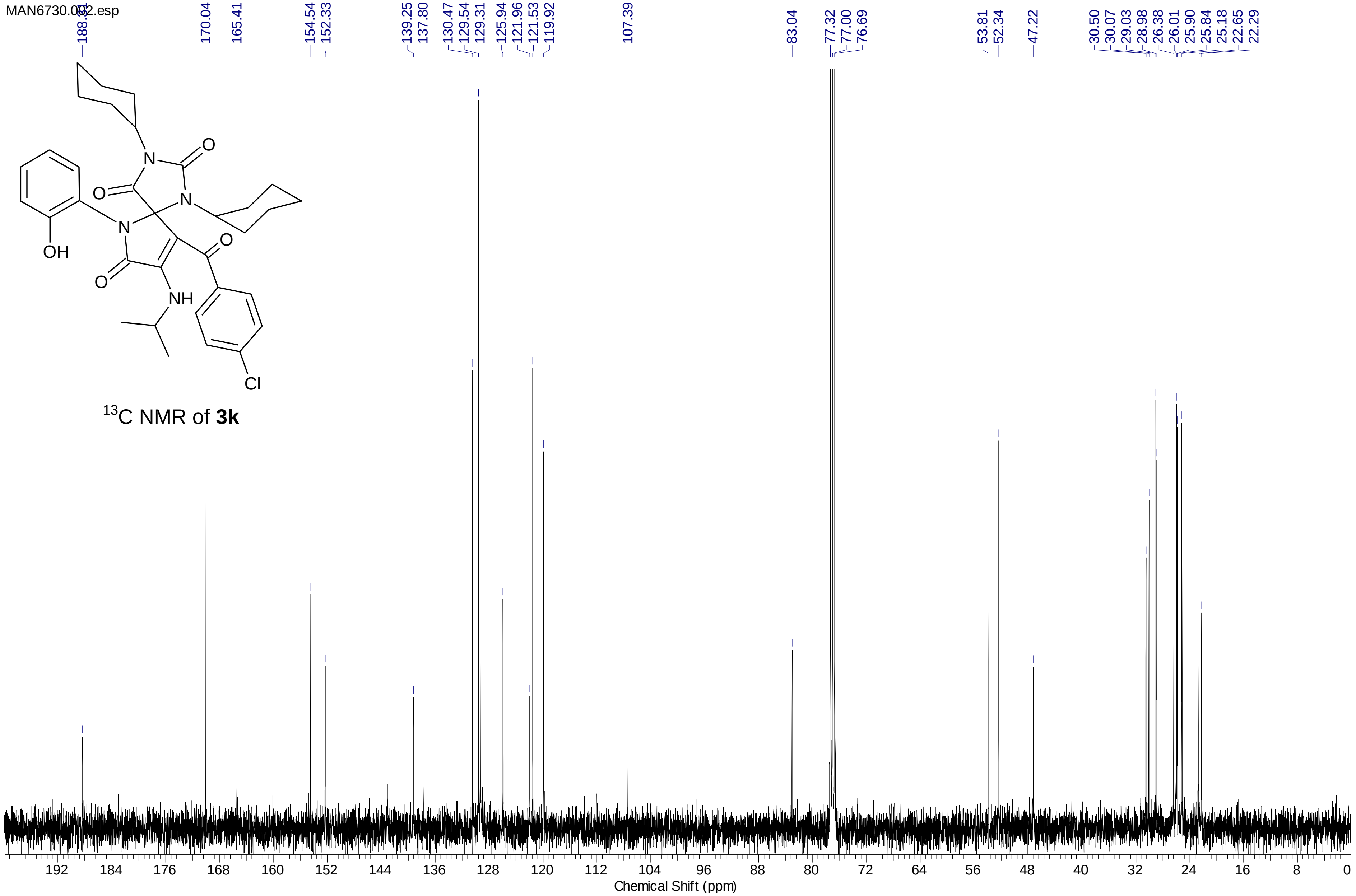
MAN6730.002.esp
188.81
170.04
165.41
154.54
152.33
139.25
137.80
130.47
129.54
129.31
125.94
121.96
121.53
119.92
107.39
83.04
77.32
77.00
76.69
53.81
52.34
47.22
30.50
30.07
29.03
28.98
26.38
26.01
25.90
25.84
25.18
22.65
22.29
192
184
176
168
160
152
144
136
128
120
112
104
96
88
80
72
64
56
48
40
32
24
16
8
0
Chemical Shift (ppm)

^{13}C NMR of **3k**

MAN7304.011.esp

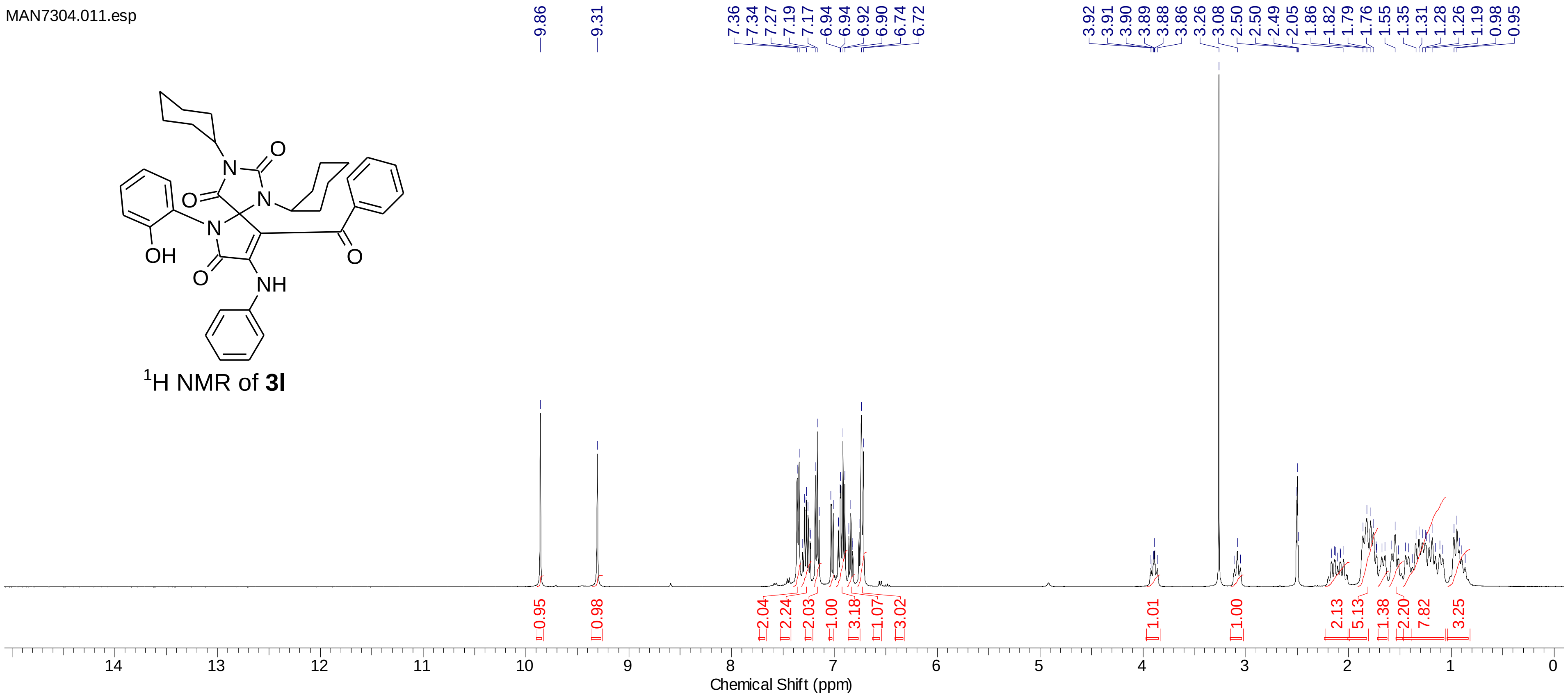

^{1}H NMR of **3l**

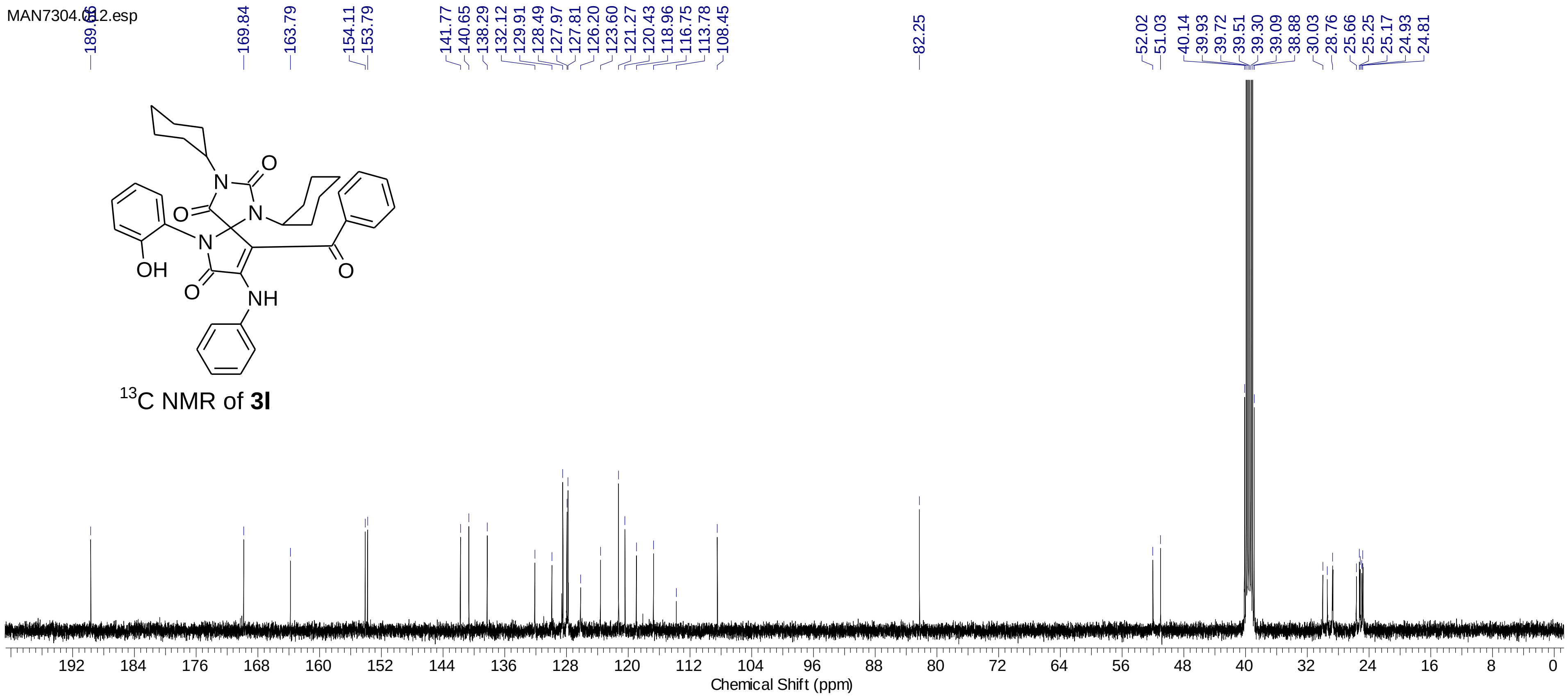
MAN7304.012.esp
189.66
169.84
163.79
154.11
153.79
141.77
140.65
138.29
132.12
129.91
128.49
127.97
127.81
126.20
123.60
121.27
120.43
118.96
116.75
113.78
108.45
82.25
52.02
51.03
40.14
39.93
39.72
39.51
39.30
39.09
38.88
30.03
28.76
25.66
25.25
25.17
24.93
24.81
O
N
O
N
N
OH
O
NH
O
192
184
176
168
160
152
144
136
128
120
112
104
96
88
80
72
64
56
48
40
32
24
16
8
0
Chemical Shift (ppm)
^{13}C NMR of **3l**

MAN7305.001.esp

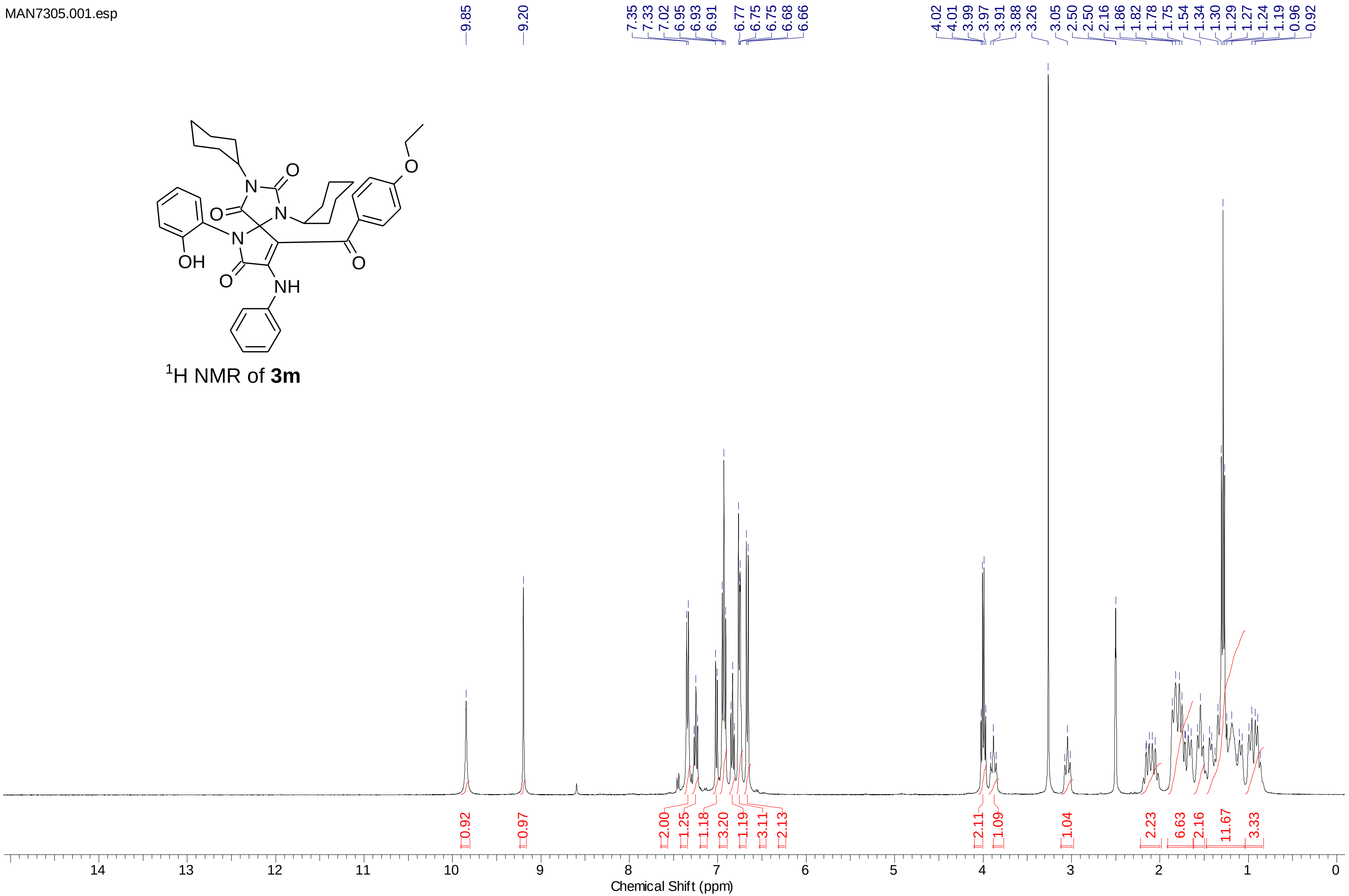

^{1}H NMR of **3m**

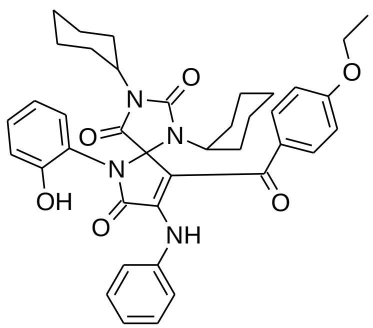

^{13}C NMR of **3m**

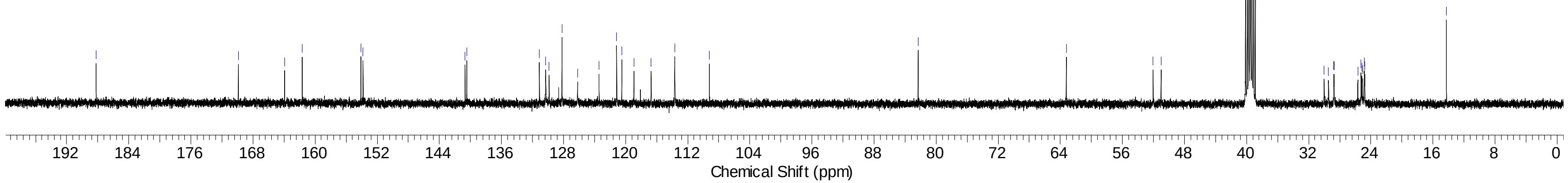

MAN7305.002.esp
188.02
169.89
163.94
161.66
154.11
153.82
140.69
140.46
131.11
130.31
129.85
128.20
126.19
123.42
121.15
120.51
118.93
116.71
113.66
109.20
82.32
63.22
52.04
51.01
40.14
39.93
39.72
39.51
39.30
39.09
38.88
28.76
28.72
25.26
25.18
25.01
24.82
24.77
14.25
Chemical Shift (ppm)

MAN7316.001.esp

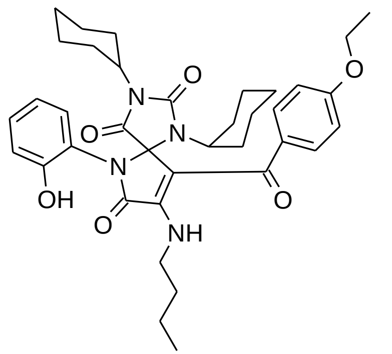

^{1}H NMR of **3n**

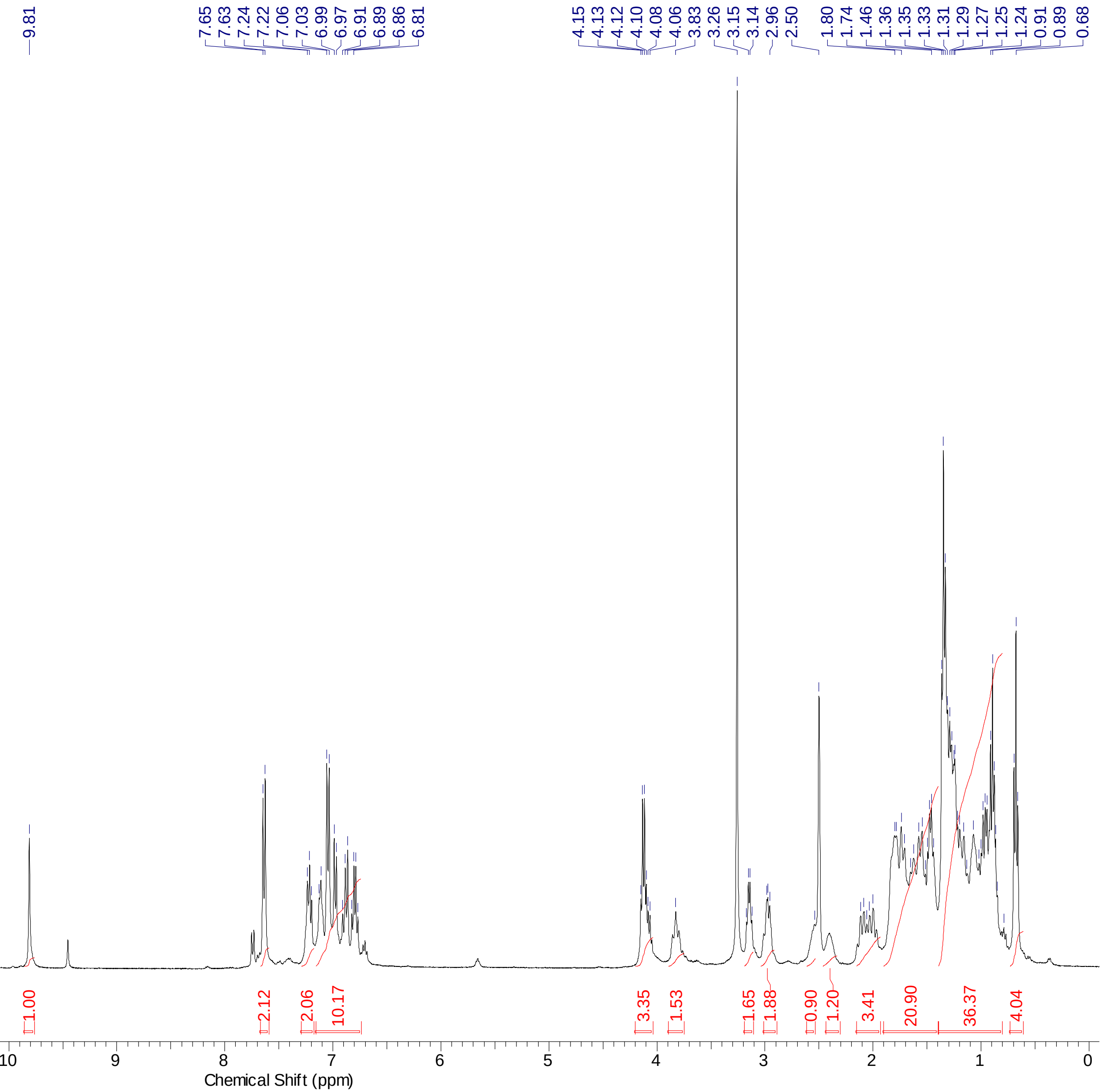

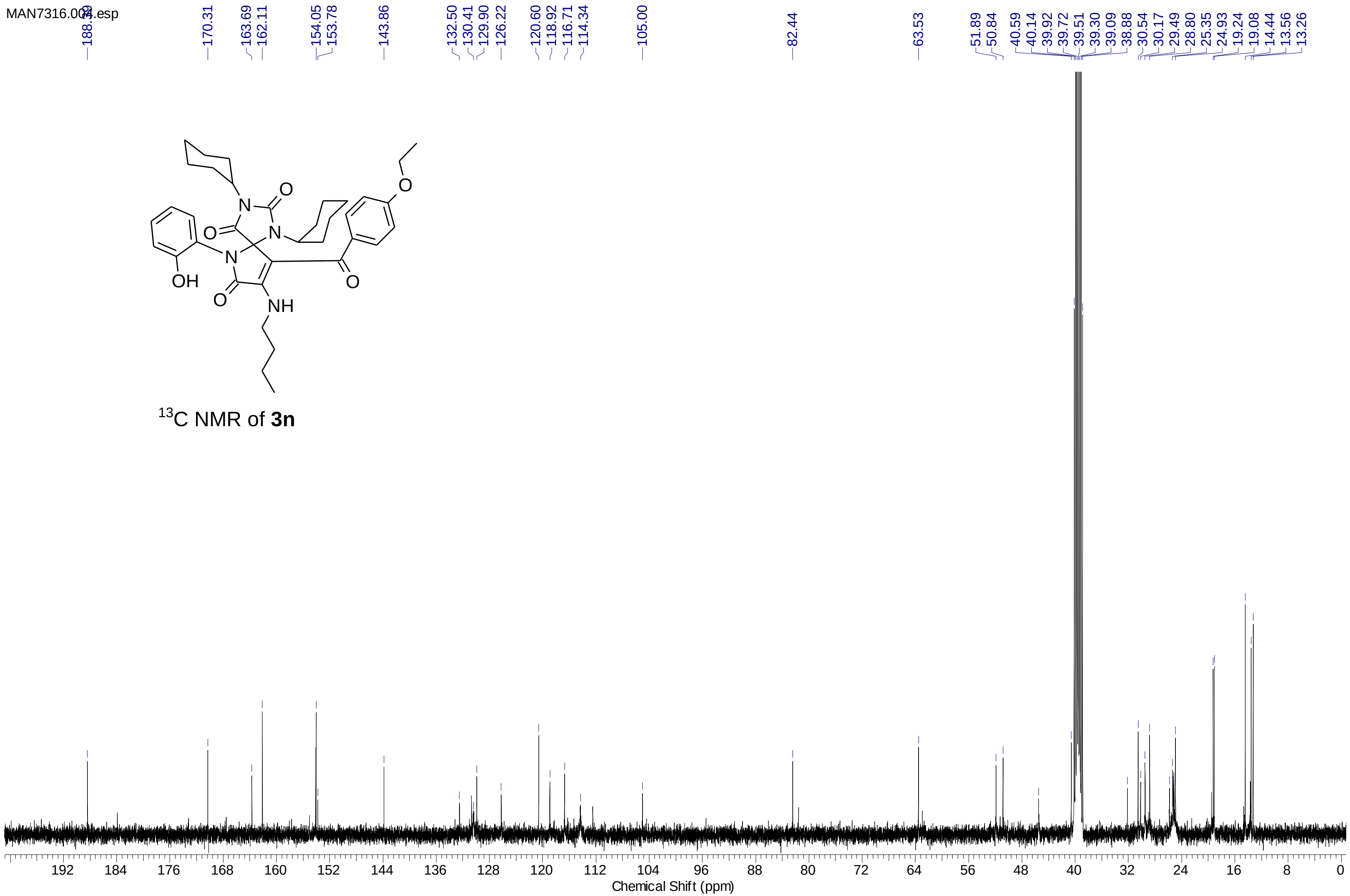
MAN7316.004.esp
188.90
170.31
163.69
162.11
154.05
153.78
143.86
132.50
130.41
129.90
126.22
120.60
118.92
116.71
114.34
105.00
82.44
63.53
51.89
50.84
40.59
40.14
39.92
39.72
39.51
39.30
39.09
38.88
30.54
30.17
29.49
28.80
25.35
24.93
19.24
19.08
14.44
13.56
13.26
Chemical Shift (ppm)

^{13}C NMR of **3n**

Antimicrobial assay by CO-ADD (ESKAPE pathogens)

Table S1. Results of antimicrobial assay

CompoundID	CompoundName	ProjectID	RunID	Sel	Act	Sa	Ec	Kp	Pa	Ab	Ca	Cn	Conc
C0104150	**4f**	P0795	PSR00168	0	0	-1,39	-3,02	12,92	-2,62	30,82	3,87	-4,31	32 ug/mL
C0104151	**4g**	P0795	PSR00168	0	0	9,9	3,02	4,69	1,77	17,58	5	0,46	32 ug/mL
C0104147	**4d**	P0795	PSR00168	0	0	-2,6	-5,81	23,53	-2,06	30,26	1,1	-4,56	32 ug/mL
C0104149	**4e**	P0795	PSR00168	0	0	12,12	3,83	10,17	1,92	12,75	2,25	1,15	32 ug/mL
C0104144	**4a**	P0795	PSR00168	0	0	4,44	-0,97	-2,12	11,09	7,35	3,08	27,12	32 ug/mL
C0104145	**4h**	P0795	PSR00168	0	0	12,75	3,02	-0,22	16,5	11,79	4,57	21,91	32 ug/mL
C0104146	**4c**	P0795	PSR00168	0	0	19,36	1,82	17,67	10,65	21,7	3,31	7,84	32 ug/mL
C0104136	**1f**	P0795	PSR00168	0	0	9,78	6,51	18,57	0,56	21,94	9,92	9,46	32 ug/mL
C0104137	**1b**	P0795	PSR00168	0	0	14,73	7,28	12,78	2,57	13,75	9,15	3,23	32 ug/mL
C0104132	**1c**	P0795	PSR00168	0	0	6,3	0,21	14,32	7,29	9,61	15,34	25,35	32 ug/mL
C0104134	**1d**	P0795	PSR00168	0	0	24,4	6,89	15,4	1,71	21,83	9,71	6,23	32 ug/mL
C0104135	**1e**	P0795	PSR00168	0	0	14,52	4,02	22,39	4,57	19,3	32,79	3,23	32 ug/mL
C0082953	**1g**	P0712	PSR00164	0	0	8,47	4,16	10,52	0,39	29,47	4,58	-7,88	32 ug/mL
C0082950	**1a**	P0712	PSR00164	0	0	7,03	0,66	5,68	-5,94	23,84	3,84	-8,56	32 ug/mL

Table S2. Tested microorganisms' strains

Abbreviation	Code	Name	Description	Strain	Organsim	Type
Sa	GP_020	*Staphylococcus aureus*	MRSA	ATCC 43300	Bacteria	G+ve
Ec	GN_001	*Escherichia coli*	FDA control	ATCC 25922	Bacteria	G-ve
Kp	GN_003	*Klebsiella pneumoniae*	MDR	ATCC 700603	Bacteria	G-ve
Ab	GN_034	*Acinetobacter baumannii*	Type strain	ATCC 19606	Bacteria	G-ve
Pa	GN_042	*Pseudomonas aeruginosa*	Type strain	ATCC 27853	Bacteria	G-ve

Ca	FG_001	*Candida albicans*	CLSI reference	ATCC 90028	Fungi	Yeast
Cn	FG_002	*Cryptococcus neoformans var. grubii*	Type strain	H99; ATCC 208821	Fungi	Yeast

Antibacterial data collection

Inhibition of bacterial growth was determined measuring absorbance at 600 nm (OD_{600}), using a Tecan M1000 Pro monochromator plate reader. The percentage of growth inhibition was calculated for each well, using the negative control (media only) and positive control (bacteria without inhibitors) on the same plate as references.

Antifungal data collection

Growth inhibition of *C. albicans* was determined measuring absorbance at 530 nm (OD_{530}), while the growth inhibition of *C. neoformans* was determined measuring the difference in absorbance between 600 and 570 nm ($OD_{600\text{-}570}$), after the addition of resazurin (0.001% final concentration) and incubation at 35 °C for additional 2 h. The absorbance was measured using a Biotek Synergy HTX plate reader. The percentage of growth inhibition was calculated for each well, using the negative control (media only) and positive control (bacteria without inhibitors) on the same plate as references.

Inhibition (Table 1)

Percentage growth inhibition of an individual sample is calculated based on Negative controls (media only) and Positive Controls (bacterial/fungal media without inhibitors). Please note negative inhibition values indicate that the growth rate (or OD600) is higher compared to the Negative Control (Bacteria/fungi only, set to 0% inhibition). The growth rates for all bacteria and fungi has a variation of -/+ 10%, which is within the reported normal distribution of bacterial/fungal growth. Any significant variation (or outliers/hits) is identified by the modified Z-Score, and actives are selected by a combination of inhibition value and Z-Score.

Z-Score

Z-Score analysis is done to investigate outliers or hits among the samples. The Z-Score is calculated based on the sample population using a modified Z-Score method which accounts for possible skewed sample population. The modified method uses median and MAD (median average derviation) instead of average and sd, and a scaling factor [Iglewicz, B. & Hoaglin, D. C. Volume 16: How to Detect and Handle Outliers. The ASQC Basic Reference in Quality Control: Statistical Techniques, 1993]: $M(i) = 0.6745 * (x(i) - median(x))/MAD$. M(i) values of > |2.5| (absolute) label outliers or hits.

Quality Control

All screening is performed as two replica (n=2), with both replicas on different assay plates, but from single plating and performed in a single screening experiment (microbial incubation). Each individual value is reported in the table (see ..1 and ..2). In addition, two values are used as quality controls for individual plates: Z'-Factor [1 - (3 * (sd(NegCtrl)+sd(PosCtrl))/(average(PosCtrl)-average(NegCtrl)))] and Standard Antibiotic controls at different concentrations (>MIC and < MIC). The plate passes the quality control if Z'-Factor >0.4 and Standards are active and inactive at highest and lowest concentrations, respectively. Data not supplied.

Selection of Actives

A - [Active] Samples with inhibition values equal to or above 80% and abs(Z-Score) above |2.5| for either replicate (n=2 on different plates) were classed as active.

P - [Partial Active] compounds with inhibition values between 50.9% - 79.9% or abs(Z-Sore) below |2.5|.

I - Inactive compounds with inhibition values below 50% and/or abs(Z-Sore) below |2.5|.

Act_XX

Act_XX: Indicates if a compound is active in any of the assays against a specific organism (Sa, Ec, Kp, Pa, Kp, Ca or Cn), or organism classses (GN: Gram-negative, GP: Gram-positive). Please note that the flag indicates single activities even if the average Inhibition values suggests otherwise, in which case a manual adjustment of the flag might be appropiate.

Act

Act: Indicates the number of organism-classes (GN,GP and FG) the compound has been found active against, 0 = no activity.

Sel

Sel: Indicates compounds that have been selected for further dose response studies, Hit-Confirmation. The selection includes all active as well as compounds with ambiguous results requiring confirmation of activity or inactivity.
